# Effects of Inappropriate Administration of Empirical Antibiotics on Mortality in Adults With Bacteraemia: Systematic Review and Meta-Analysis

**DOI:** 10.3389/fmed.2022.869822

**Published:** 2022-05-30

**Authors:** Yuan-Pin Hung, Ching-Chi Lee, Wen-Chien Ko

**Affiliations:** ^1^Department of Internal Medicine, Tainan Hospital, Ministry of Health and Welfare, Tainan City, Taiwan; ^2^Department of Internal Medicine, College of Medicine, National Cheng Kung University Hospital, National Cheng Kung University, Tainan City, Taiwan; ^3^Department of Medicine, College of Medicine, National Cheng Kung University, Tainan City, Taiwan; ^4^Clinical Medicine Research Centre, College of Medicine, National Cheng Kung University Hospital, National Cheng Kung University, Tainan City, Taiwan

**Keywords:** empirical antibiotic, bacteraemia, mortality, systematic review, meta-analysis

## Abstract

**Introduction:**

Bloodstream infections are associated with high mortality rates and contribute substantially to healthcare costs, but a consensus on the prognostic benefits of appropriate empirical antimicrobial therapy (EAT) for bacteraemia is lacking.

**Methods:**

We performed a systematic search of the PubMed, Cochrane Library, and Embase databases through July 2021. Studies comparing the mortality rates of patients receiving appropriate and inappropriate EAT were considered eligible. The quality of the included studies was assessed using Joanna Briggs Institute checklists.

**Results:**

We ultimately assessed 198 studies of 89,962 total patients. The pooled odds ratio (OR) for the prognostic impacts of inappropriate EAT was 2.06 (*P* < 0.001), and the funnel plot was symmetrically distributed. Among subgroups without between-study heterogeneity (*I*^2^ = 0%), those of patients with severe sepsis and septic shock (OR, 2.14), Pitt bacteraemia scores of ≥4 (OR, 1.88), cirrhosis (OR, 2.56), older age (OR, 1.78), and community-onset/acquired Enterobacteriaceae bacteraemia infection (OR, 2.53) indicated a significant effect of inappropriate EAT on mortality. The pooled adjusted OR of 125 studies using multivariable analyses for the effects of inappropriate EAT on mortality was 2.02 (*P* < 0.001), and the subgroups with low heterogeneity (*I*^2^ < 25%) exhibiting significant effects of inappropriate EAT were those of patients with vascular catheter infections (adjusted OR, 2.40), pneumonia (adjusted OR, 2.72), or Enterobacteriaceae bacteraemia (adjusted OR, 4.35). Notably, the pooled univariable and multivariable analyses were consistent in revealing the negligible impacts of inappropriate EAT on the subgroups of patients with urinary tract infections and *Enterobacter* bacteraemia.

**Conclusion:**

Although the current evidence is insufficient to demonstrate the benefits of prompt EAT in specific bacteraemic populations, we indicated that inappropriate EAT is associated with unfavorable mortality outcomes overall and in numerous subgroups. Prospective studies designed to test these specific populations are needed to ensure reliable conclusions.

**Systematic Review Registration:**

https://www.crd.york.ac.uk/prospero/, identifier: CRD42021270274.

## Introduction

Bacteraemia is associated with a high mortality rate and contribute substantially to healthcare costs (1). Antibiotic therapy, both empirical and definitive, is the mainstay of treatment for such systemic infections. Although studies have extensively researched the association between the appropriate administration of empirical antimicrobial therapy (EAT) and short-term mortality outcomes in sepsis (2), the potential benefits of appropriate EAT in bloodstream infections remain unknown. Numerous studies have reported EAT to have trivial effects (3–6), whereas others have reported a beneficial fatality rate reduction (7–10). We believe that these variations in results are due to differences among studies in the bacteraemia severity, host immunity status, target patient populations, causative microorganisms, and bacteraemia source. Therefore, we conducted a systematic review and meta-analysis to assess the effects of appropriate EAT on mortality in specific clinical scenarios, and such an assessment is essential for the optimal antimicrobial stewardship.

## Methods

### Study Selection

#### Study Design

This analysis followed the updated Preferred Reporting Items for Systematic Reviews and Meta-Analyses (PRISMA) guidelines (11), and our protocol was registered at the International Prospective Register of Systematic Reviews under the registration number CRD42021270274. We considered case–control studies, cohort studies, and clinical trials on adult patients with bacteraemia, without restrictions regarding onset location, causative microorganisms, antimicrobial-resistant microorganisms, sources of bacteraemia, illness severity, or comorbidities. Reviews, guideline articles, case reports, duplicate studies, studies with patient overlap, and studies on patients without bacteraemia were excluded. In the full-text assessment of eligible articles, articles were excluded if they (i) included participants aged <18 years, (ii) did not assess EAT or distinguish it from definitive treatment, (iii) did not assess the EAT–mortality association, (iv) did not define EAT, or (v) were published in a language other than English.

#### Patients

The patients included were adults with microbiologically documented bloodstream infections.

#### Intervention

The intervention was inappropriate (vs. appropriate) administration of empirical antimicrobials. An antimicrobial *in vitro* active against the causative microorganism was regarded as “appropriate” treatment. “Empirical” therapy was defined as that administered at initial sampling of blood cultures, within 24, 48, and 72 h, or 5 days after culture sampling, or prior to culture result.

#### Outcome

The primary outcomes in this meta-analysis included short-term (i.e., 7-day, 14-day, 21-day, 28- or 30-day), in-hospital, and long-term mortality.

### Literature Search

The PubMed, Cochrane Library, and Embase databases were searched from their inception to July 2021 for articles on appropriate or inappropriate antimicrobial administration in adults with bacteraemia. The following terms for searches were applied: (antibiotic OR antimicrobial) AND (inappropriate OR appropriate) AND (empirical or initial) AND (bacteremia OR bacteraemia OR bloodstream) AND (mortality OR fatality OR death OR dead OR alive OR survival) NOT (children OR neonate OR adolescent OR infant OR pediatric). Details regarding the search strategy are presented in Supplementary Table 1. Additional studies were identified by perusing reference lists of systematic reviews.

### Study Selection, Data Extraction, and Quality Assessment

Firstly, the results of the literature searches were screened based on study eligibility criteria and discrepancies were periodically resolved by consensus in the team conference. Focusing on the included studies, the extracted data included the study design, types and severity of patient comorbidities, sources of bacteraemia, causative microorganism, the type and proportion of antibiotic-resistant isolates, inclusion and exclusion criteria, EAT definition, the percentage of patients receiving appropriate EAT, mortality, results of unadjusted and adjusted analyses, and covariates adjusted for in multivariable analyses.

Because none of randomized clinical trials or studies was recognized in our systematic review, the quality of the included studies was evaluated using the Newcastle-Ottawa Quality (NOQ) assessment for all the included cohort or case-control studies (12). This assessment includes the appropriateness of the cohort selection and comparison between case and control groups, outcome evaluation, and patient follow-up. The maximum score of the NOQ assessment is 9 (the highest quality), and the scores of 7–9, 4–6, and 0–3 were regarded as the high, moderate, and low quality of studies, respectively (12).

Two investigators (C.-C.L. and Y.-P. H.) independently screened the search results according to exclusion criteria, recorded the clinical information, and assessed study quality from each study; and a third investigator (W.-C.K.) was consulted to resolve any disagreements during periodic meetings.

### Data Synthesis and Analysis

#### Unadjusted Analysis

We computed the numbers of assessed and fatal patients for individual studies and pooled them for the meta-analysis. We investigated heterogeneity through subgroup analyses based on the following: infection acquisition (community-onset/acquired, hospital-onset/acquired, or healthcare-associated), comorbidity types (liver cirrhosis or haemato-oncological), age (≥65 years), neutropenia status, bacteraemia sources (vascular catheter, pneumonia, or biliary tract, or urinary tract), bacteraemia severity (intensive care unit [ICU] admission, severe sepsis and septic shock, Pitt bacteraemia score of ≥4 at onset, or non-ICU admission), microbial groups (Enterobacteriaceae or glucose non-fermentative rods), specific microorganisms (*Staphylococcus aureus, Escherichia coli, Klebsiella pneumoniae, Enterobacter* spp., *Pseudomonas* spp., or *Acinetobacter* spp.), and antibiotic-resistant microorganisms (methicillin-resistant *S. aureus*, extended-spectrum beta-lactamase [ESBL]-producing Enterobacteriaceae, multidrug-resistant [MDR] Enterobacteriaceae, or carbapenem-resistant Enterobacteriaceae). Moreover, because of the different EAT cut-offs and mortality assessments of each study, the subgroup analyses included subgroups of various EAT delays (0, 24, 48, and 72 h, 5 days, or prior to culture results) and different (7-, 14-, 21-, 28-, or 30-day; in-hospital; or long-term) mortality measures after the initial culture sampling.

#### Adjusted Analysis

Of the 198 studies initially included, 73 did not report an adjusted analysis and were therefore excluded because we could not input adjusted odds ratios (ORs) for our analyses. Among the 125 studies that used multivariate analyses, 16 reported nonsignificant results but no numerical data; another reported a significant multivariable result but no numerical data; and the remaining 108 studies provided their multivariate results in terms of adjusted ORs and 96% confidence intervals (CIs). For these 108 studies, the adjusted ORs for mortality of inappropriate EAT were inputted for 84 studies that directly reported the effects of inappropriate EAT. For the remaining 24 studies that only demonstrated the effect of appropriate EAT on mortality, the adjusted ORs and 95% CIs were inversely inputted. Moreover, for the 16 studies that reported nonsignificant effects of inappropriate EAT without providing any numerical data, an adjusted OR of 1 and the 95% CI from the univariable analysis were inputted as a dispersion measure. For one study reporting a significant effect of inappropriate EAT but no numerical data from multivariable analysis, the OR and 95% CI were inputted from univariable analyses. Accordingly, our main adjusted analyses included all studies that assessed the effects of inappropriate EAT on mortality through multivariable regression. As in the univariable analysis, further subgroup analyses were performed to minimize the effects of between-study heterogeneity.

### Statistical Methods

Consistent with the previously established methods (13, 14), irrespective of unadjusted and adjusted ORs, the meta-analysis was conducted to recognize the pooled effects of inappropriate ETA on patient outcomes, and adopted a random-effects model under the assumption that the considerably heterogeneity in study results is due to the diverse study populations and multivariate regression models used for adjusting for confounding factors. Between-study heterogeneity and consistency were assessed using Cochran's *Q* test and *I*^2^; we aimed to eliminate this heterogeneity through the subgroup analyses. To assess the effect of small-population studies, a funnel plot of standard errors against ORs was constructed for the univariate and multivariate results of all included studies. All analyses were performed using Review Manager (version 5.3).

## Results

**Figure 1:**
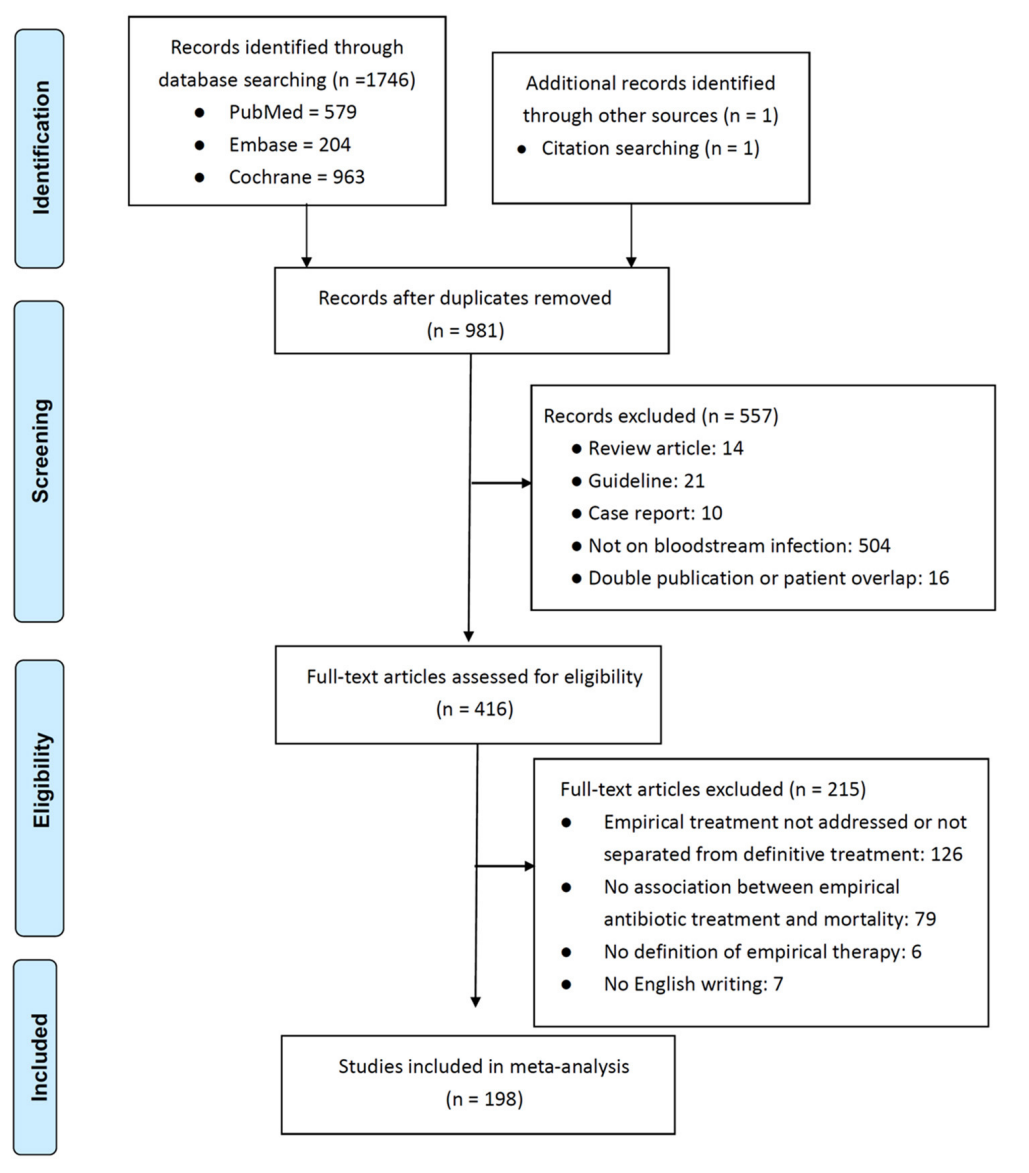
Study flow.

In total, 198 studies (3–10, 15–204), were selected from 981 potentially relevant studies on the basis of the inclusion and exclusion criteria (Figure 1). Total 89,962 patients were assessed; the majority (*n* = 64,008, 71.2%) received appropriate EAT, and the overall mortality rate was 21.3% (*n* = 19,181). The publication year, geographic location, design, acquisition sources, bacteraemia sources, target patient population, causative microorganisms, mortality rate, numbers of patients receiving appropriate EAT, ORs and adjusted ORs of inappropriate EAT, and study quality of each study are presented in Supplementary Table 2. The majority (190 studies) of the included studies were cohort studies and the remaining eight studies were case–control studies, which had a median (interquartile range [IQR]) NOQ score of 7 (6–7).

### Univariate (Unadjusted) Analysis for Mortality

For the 89,962 patients assessed in 198 studies, the pooled OR for prognostic impacts of inappropriate EAT was 2.06 (95% CI, 1.88–2.25; *P* < 0.001), as shown in Supplementary Figure 1A; and these studies were symmetrically distributed around the pooled OR in the funnel plot (Supplementary Figure 1B). Because considerable between-study heterogeneity was observed (*P* < 0.001, *I*^2^ = 72%), subgroup meta-analyses based on the acquisition sources, bacteraemia sources, target patient populations, causative microorganisms, differential timeline cutoffs (after initial sampling of blood cultures) for EAT definition, and varied timeline cutoffs assessed for mortality outcomes were conducted (Table 1). The detailed forest plots for these subgroup analyses are presented in Supplementary Figure 2. The effects of inappropriate EAT remained significant in nearly all subgroups (Table 1); however, the between-study heterogeneity remained significant in most of the subgroups. Among the eight subgroups without between-study heterogeneity (*I*^2^ = 0%), inappropriate EAT was significantly associated with mortality in the following: patients experiencing severe sepsis and septic shock (OR, 2.14; 95% CI, 1.81–2.53), patients with Pitt bacteraemia scores of ≥4 (OR, 1.88; 95% CI, 1.33–2.67), patients with cirrhosis (OR, 2.56; 95% CI, 2.02–3.26), patients aged >65 years (OR, 1.78; 95% CI, 1.38–2.31), patients with community-onset/acquired Enterobacteriaceae bacteraemia (OR, 2.53; 95% CI, 1.63–3.92), and studies with an EAT assessment period of <5 days (OR, 3.00; 95% CI, 1.92–4.69). Conversely, the effect of inappropriate EAT on mortality was negative among non–ICU patients (OR, 0.90; 95% CI, 0.59–1.36) and patients with urinary tract infection–induced bacteraemia (OR, 1.31; 95% CI, 0.86–1.98). Notably, between-study heterogeneity remained significant in each subgroup detailing the varied assessment periods for EAT or mortality, but the association of inappropriate EAT with mortality was consistently significant.

**Table 1:** Unadjusted analyses in overall and subgroup patients.

**Characteristics/subgroups**	**Study No**.	**Patients No**.	**Unadjusted OR of inappropriate EAT (95% CI)**	***I^**2**^ (%)***	***P*-value**
All patients	198	89,926	2.06 (1.88–2.25)	78	<0.001
Location of onset					
Community	18	12,766	2.49 (1.90–3.27)	72	<0.001
Hospital	28	6,508	2.33 (1.76–3.07)	76	<0.001
Healthcare-associated	4	2,442	2.32 (1.13–4.78)	92	<0.001
Bacteraemia severity					
ICU patients	16	6,356	2.58 (1.88–3.54)	82	<0.001
Severe sepsis and septic shock	5	2,793	2.14 (1.81–2.53)	0	<0.001
Pitt bacteraemia score ≥4 at onset	2	776	1.88 (1.33–2.67)	0	<0.001
Non-ICU patients	3	827	0.90 (0.59–1.36)	0	0.61
Specific population					
Comorbid haemato-oncology	11	5,822	3.10 (1.85–5.19)	90	<0.001
Comorbid liver cirrhosis	5	1,674	2.56 (2.02–3.26)	0	<0.001
Older patients (≥65 years)	4	2,955	1.78 (1.38–2.31)	0	<0.001
Neutropenia	4	1,789	2.48 (0.85–7.30)	72	0.10
Bacteraemia source					
Vascular catheter	5	1,493	1.46 (0.92–2.32)	61	0.11
Pneumonia	9	1,987	2.02 (1.28–3.20)	61	0.002
Biliary tract	4	2,675	1.71 (1.11–2.64)	46	0.02
Urinary tract	4	1,763	1.31 (0.86–1.98)	0	0.21
Causative microorganism					
*Staphylococcus aureus*					
Overall	24	7,228	1.71 (1.36–2.15)	68	<0.001
Hospital-onset/acquired	4	792	1.31 (0.54–3.19)	86	0.55
Community-acquired	1	86	2.85 (0.91–8.92)	-	0.07
Enterobacteriaceae					
Overall	45	13,760	2.01 (1.55–2.61)	81	<0.001
Hospital-onset/acquired	5	806	2.87 (1.62–5.05)	62	<0.001
Community-onset/acquired	4	1,077	2.53 (1.63–3.92)	0	<0.001
Glucose non-fermentative rods	34	6,961	2.20 (1.73–2.79)	68	<0.001
Specific species					
*Escherichia coli*	14	7,371	2.77 (1.62–4.72)	80	<0.001
*Klebsiella pneumonia*	13	3,557	2.02 (1.46–2.80)	59	<0.001
*Enterobacter* spp.	5	921	1.56 (0.93–2.62)	48	0.09
*Pseudomonas* spp.	17	4,866	1.74 (1.45–2.10)	25	<0.001
*Acinetobacter* spp.	16	1,919	2.55 (1.53–4.27)	77	<0.001
Antibiotic-resistant microorganism					
MRSA	9	2,061	1.70 (1.10–2.63)	81	0.02
ESBL-producing Enterobacteriaceae	13	1,795	1.39 (0.85–2.28)	77	0.19
MDR Enterobacteriaceae	2	366	1.80 (0.62–5.21)	77	0.28
Carbapenem-resistant Enterobacteriaceae	8	845	2.45 (1.16–5.17)	80	0.02
EAT timeliness regard to initial culture					
0 h	32	13,288	2.15 (1.63–2.85)	86	<0.001
<24 h	69	33,554	1.95 (1.72–2.22)	73	<0.001
<48 h	43	17,029	1.92 (1.59–2.32)	76	<0.001
<72 h	9	909	2.49 (1.34–4.62)	64	0.004
<5 days	3	383	3.00 (1.92–4.69)	0	<0.001
Prior to culture result	49	28,684	2.02 (1.66–2.46)	79	<0.001
Mortality timeline regard to initial culture					
≤ 7 days	10	3,408	6.83 (3.40–13.73)	85	<0.001
≤ 14 days	20	4,118	2.12 (1.63–2.77)	54	<0.001
≤ 21 days	8	1,049	4.41 (2.11–9.19)	81	<0.001
≤ 28 or 30 days	114	59,868	1.79 (1.59–2.00)	77	<0.001
In-hospital	59	23,013	2.36 (2.01–2.78)	78	<0.001
Long-term	4	7,426	1.56 (1.21–2.00)	62	<0.001

*CI, confidence interval; EAT, empirical antimicrobial therapy; ESBL, extended-spectrum beta-lactamase; ICU, intensive care unit; MDR, multi-drug resistant; MRSA, Methicillin-resistant Staphylococcus aureus; OR, odds ratio*.

### Multivariate (Adjusted) Analysis for Mortality

Of the 125 studies reporting adjusted multivariable results for mortality risk, the pooled adjusted OR for prognostic impacts of inappropriate EAT was 2.02 (95% CI, 1.86–2.49), with an asymmetrical distribution of studies around the pooled OR in the funnel plot (Supplementary Figure 3). Because considerable heterogeneity was observed (*I*^2^ = 92%, *P* < 0.001), we conducted further subgroup analyses (Table 2). The forest plots of these subgroup analyses are presented in Supplementary Figure 4. The effects of inappropriate EAT remained significant in nearly all subgroups, and the between-study heterogeneity remained considerable in most subgroups. Among the subgroups without heterogeneity, inappropriate EAT remained significant impacts in the subgroups of patients acquiring bacteraemia from a vascular catheter (adjusted OR, 2.40; 95% CI, 1.63–3.53) or pneumonia (adjusted OR, 2.72; 95% CI, 2.07–3.57), those with mixed Enterobacteriaceae bacteraemia (adjusted OR, 4.35; 95% CI, 1.28–14.76), and those in studies measuring mortality within 7 (adjusted OR, 3.08; 95% CI, 1.98–4.79) or 14 (adjusted OR, 2.31; 95% CI, 1.72–3.09) days after the initial culture sampling. However, the prognostic impact of inappropriate EAT was nonsignificant in patients with *Enterobacter* bacteraemia (OR, 1.00; 95% CI, 0.89–1.12). As in the univariable analyses, considerable heterogeneity remined in all subgroups with different assessment periods of EAT and mortality outcomes, and the predictive ability of inappropriate EAT was significant in all these subgroups.

**Table 2:** Adjusted analyses in overall and subgroup patients.

**Characteristics/ subgroups**	**Study No**.	**Adjusted OR (95% CI) of inappropriate EAT**	***I^**2**^ (%)***	***P*-value**
Overall	125	2.02 (1.86–2.20)	92	<0.001
Location of onset				
Community	12	1.95 (1.58–2.41)	50	<0.001
Hospital	23	1.92 (1.55–2.39)	92	<0.001
Healthcare-associated	3	2.07 (0.97–4.46)	77	0.06
Bacteraemia severity				
ICU patients	14	2.26 (1.56–3.28)	91	<0.001
Severe sepsis and septic shock	2	2.76 (1.47–5.20)	90	<0.001
Pitt bacteraemia score ≥ 4	2	2.02 (1.07–3.81)	58	<0.001
Specific population				
Comorbid haemato-oncology	7	2.50 (1.41–4.43)	57	0.002
Comorbid liver cirrhosis	5	3.70 (1.90–7.20)	90	<0.001
Older patients (≥ 65 years)	3	1.36 (0.86–2.15)	78	0.20
Bacteraemia source				
Vascular catheter	4	2.40 (1.63–3.53)	0	<0.001
Pneumonia	4	2.72 (2.07–3.57)	0	<0.001
Biliary tract	3	1.82 (1.17–2.83)	49	0.007
Urinary tract	3	1.40 (0.82–2.38)	65	0.22
Causative microorganism				
Gram-positive cocci				
*Staphylococcus aureus*	15	2.12 (1.55–2.92)	78	<0.001
*Enterococcus* spp.	1	5.00 (2.50–10.00)	-	<0.001
*Streptococcus* spp.	1	10.60 (1.20–93.63)	-	<0.001
Enterobacteriaceae	20	1.07 (1.021.11)	83	<0.001
*Escherichia coli*	6	3.00 (2.00–4.50)	73	<0.001
*Klebsiella pneumonia*	9	1.05 (1.00–1.10)	81	0.04
*Enterobacter* spp.	2	1.00 (0.89–1.12)	8	1.00
*Proteus* spp.	1	9.85 (2.67–36.34)	-	<0.001
Mixed	2	3.47 (1.74–6.93)	2	<0.001
Glucose non-fermentative rod	27	1.09 (1.04–1.14)	85	<0.001
*Pseudomonas* spp.	12	1.05 (0.99–1.10)	82	0.08
*Acinetobacter* spp.	13	1.28 (1.15–1.43)	87	<0.001
*Burkholderia* spp.	1	23.92 (1.31–435.86)	-	0.03
Mixed	1	4.35 (1.28–14.76)	-	0.02
Antibiotic-resistant microorganism				
MRSA	6	2.34 (1.30–4.21)	89	0.004
ESBL-producing Enterobacteriaceae	6	2.03 (1.05–3.93)	71	0.04
Carbapenem-resistant Enterobacteriaceae	4	2.40 (1.21–4.74)	77	0.01
EAT timeliness regard to initial culture				
0 h	14	1.76 (1.35–2.30)	72	<0.001
<24 h	46	2.08 (1.77–2.44)	91	<0.001
<48 h	30	2.45 (1.95–3.08)	75	<0.001
<72 h	6	1.70 (1.15–2.51)	81	0.007
<5 days	3	2.76 (1.27–5.09)	56	0.01
Prior to culture result	29	1.85 (1.59–2.16)	93	<0.001
Mortality timeline regard to initial culture				
≤ 7 days	5	3.08 (1.98–4.79)	11	<0.001
≤ 14 days	10	2.31 (1.72–3.09)	19	<0.001
≤ 21 days	5	3.78 (2.06–6.96)	55	<0.001
≤ 28 or 30 days	69	2.07 (1.82–2.35)	90	<0.001
In-hospital	40	1.81 (1.56–2.10)	93	<0.001
Long-term	4	1.68 (1.10–2.54)	74	0.02

*CI, confidence interval; EAT, empirical antimicrobial therapy; ESBL, extended-spectrum beta-lactamase; ICU, intensive care unit; MRSA, methicillin-resistant Staphylococcus aureus; OR, odds ratio*.

Confounding factors for mortality outcomes for potential enrolment in multivariable analyses were calculated in each individual study (Table 3). Nearly all studies (89.6%) assessed bacteraemia severity, using scores such as the Acute Physiology Age and Chronic Health Evaluation; Acute Physiology Score; Sepsis-related (Sequential) Organ Failure Assessment score; and Pitt bacteraemia score. Formal scores for comorbidity severity (i.e., Charlson comorbidity index or McCabe classification) were used in approximately 50% of the studies. Other confounding factors assessed in more than half of the studies were the bacteraemia source, acquisition source, patient age, and the presence of any comorbidity. The median (IQR) number of confounding factors included in the multivariable models was 5 (4–7), and the median (IQR) of the ratio of confounding factors to deaths was 13.0 (7.6–28.8).

**Table 3:** The distribution of confounding factors adjusted for multivariate analyses.

**Confounding factors**	**The number of studies adopted for adjusting**	**Frequencies (the numbers of studies should be analyzed by the multivariate model)**
Bacteraemia severity	118	89.6 (125)
Comorbidity severity	64	51.2 (125)
Bacteraemia source	79	66.4 (119)
Acquisition place	54	53.5 (101)
Patient demographics		
Age	63	52.1 (121)
Gender	26	20.8 (125)
Functional capacity	11	8.8 (125)
Laboratory data		
Albumin	10	8.0 (125)
C-reactive protein or procalcitonin	6	4.8 (125)
Comorbidity		
Any	78	62.4 (125)
Hemato-oncology	42	36.8 (114)
Liver disease	29	24.2 (120)
Renal disease	29	23.2 (125)
Cardiovascular disease	19	15.2 (125)
Diabetes mellitus	15	12.0 (125)
Pulmonary disease	15	12.0 (125)
Neutropenia	26	21.5 (121)
Immunosuppressive agent	35	28.0 (125)
Source control	11	9.2 (120)

### Non-specific Comorbid Patients With Overall Bacteraemia

**Figure 2:**
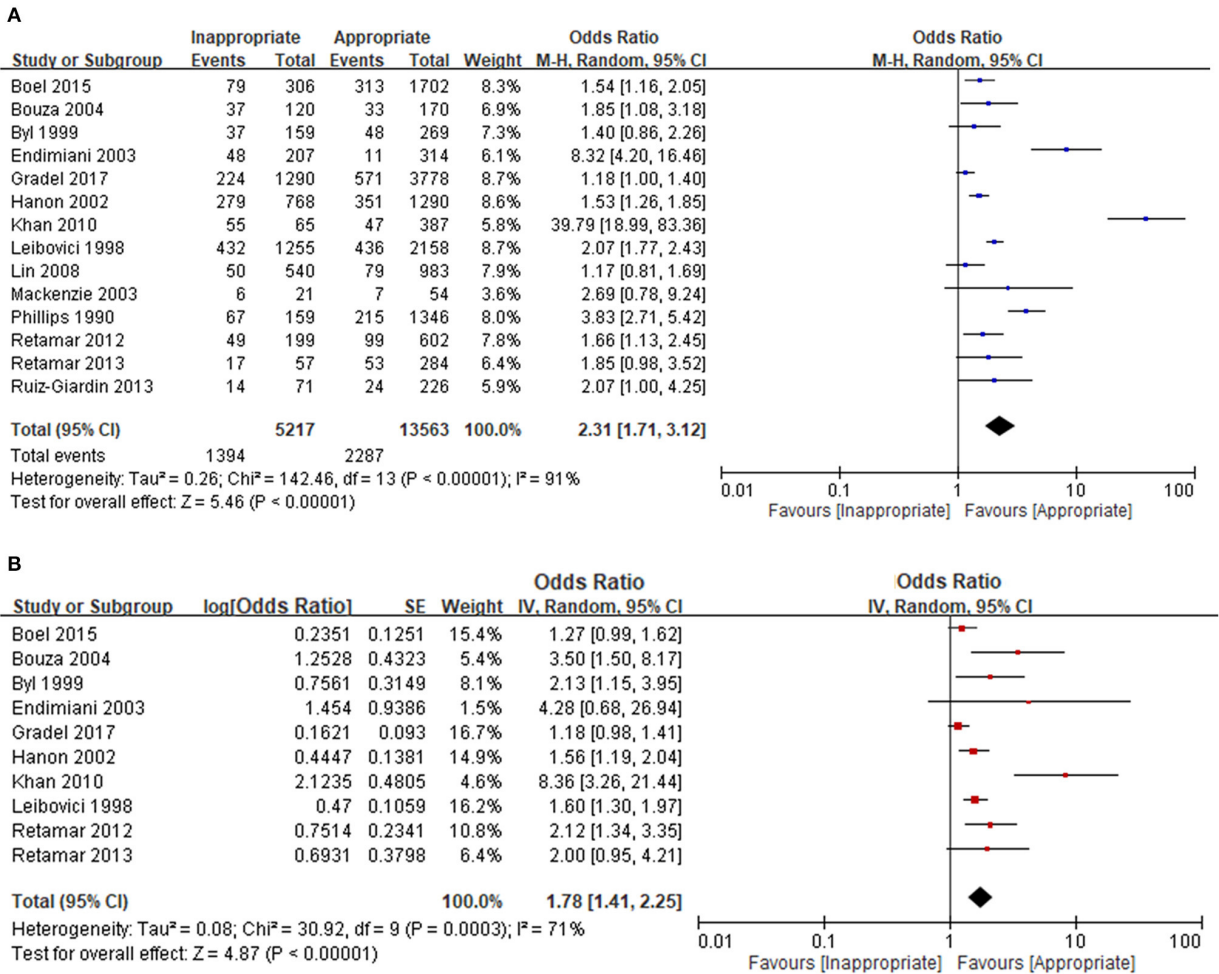
Unadjusted **(A)** and adjusted **(B)** analyses in studies dealing with overall bacteraemia. *Only included non-specific comorbid patients with bacteraemia without the unique focus, causative microorganisms, and acquisition place.

We evaluated the prognostic effect of inappropriate EAT in 14 bacteraemia studies of patients with nonspecific comorbidities and bacteraemia without the specific causative microorganism and specific acquisition source. Both the univariable (Figure 2A) and multivariable (Figure 2B) analyses revealed significant adverse effects of inappropriate EAT, with an OR of 2.31 and adjusted OR of 1.78 for mortality; the between-study heterogeneity remained considerable (*I*^2^ = 91% and 71%, respectively).

## Discussion

Bacteraemia ia an common and complex disease with mortality rates widely ranged from 1.2% (122) to 90% (63), depending on host's immune status or comorbidities, severity of illness at onset, and bacteraemia sources. Of numerous studies previously reported the prognostic effect of inappropriate EAT, the definition of “empirical” administration and mortality assessed for study outcomes were not consistent. To diminish the publication bias, the study adopted by any reasonable definition of “empirical” administration and “short- or long-term outcomes” was comprehensively enrolled in our analyses. Despite the existence of considerable between-study heterogeneity herein, the prognostic impacts of inappropriate EAT remained significant in all the subgroups categorized by different cutoff timelines of EAT or mortality. Furthermore, irrespectively of whether through univariable or multivariable analyses, the pooled effect of inappropriate EAT was significant in all included patients and its impact was nearly all evidenced in patients sub-grouped by different acquisition places, bacteraemia severity, bacteraemia sources, aimed patient populations, and causative microorganisms. Accordingly, the prognostic disadvantage of delayed administration of appropriate antimicrobials was emphasized in our analyses.

A pooled analysis of the univariable results revealed the negligible impact of inappropriate EAT on four subgroups, namely the bacteraemia source of urinary tract infections (four studies), neutropenia individuals (four), MDR-Enterobacteriaceae bacteraemia (two), and non–ICU patients (three). However, other than for studies dealing with urinary tract infections, multivariable analyses were not performed within these studies, because the majority (8/9, 88.9%) revealed similar mortality rates for patients who did not receive appropriate EAT and those who received through the univariate analyses. Currently in literature search, few studies have evaluated the prognostic effect of inappropriate EAT on these subgroups; we believe this is because the nonsignificant effects limit their publications. Although the current evidence is insufficient to highlight the prognostic disadvantage of inappropriate EAT, further studies focusing on these specific populations are warranted.

The pooled results of the univariable and multivariable analyses consistently indicated that inappropriate EAT significantly impacted the prognoses of nearly all subgroup patients. Moreover, the pooled univariable and multivariable analyses consistently revealed negligible impacts of inappropriate EAT on two subgroups: the bacteraemia source of urinary tract infections and *Enterobacter* bacteraemia. However, the pooled results of the univariable and multivariable analyses differed for the prognostic effects in several subgroups herein, including those of healthcare-associated acquisition, older patients, and bacteraemia caused by vascular catheter infections or ESBL-producing Enterobacteriaceae. In such the situation, we believe that the multivariable analysis is necessary to clarify the independent effectiveness of antimicrobial therapy. Taking the ESBL-producer as an example, its crucial association with vascular catheter infections (205), severe comorbid patients (205), older patients (41), or healthcare-associated bacteraemia (205) has been established. Moreover, the association of ESBL-producers and delayed EAT or unfavorable prognoses had been evidenced (19, 111, 144, 145). Consequently, to diminish the ESBL-producer, a crucial confounding factor, affecting the prognostic effects of inappropriate EAT, adjustments for the above parameters were essential. Accordingly, based on the pooled result of multivariable analyses herein, the prognostic effect of delayed EAT was trivial in patients with healthcare-associated bacteraemia or the older patients experiencing bacteraemia and significant in those with bacteraemia caused by vascular catheter infections or ESBL-producing Enterobacteriaceae.

This meta-analysis has several limitations. First, because of ethical concerns with testing the negative effects of inappropriate EAT on mortality, randomized clinical trials comparing the outcomes of appropriate and inappropriate EAT are limited in the literature. Therefore, consistent with that in previous meta-analyses of appropriate EAT administration in patients with sepsis (206–208), between-study heterogeneity might arise from nonrandomised studies herein. Second, in accordance with previous methods (206), we used reasonable assumptions to comprehensively capture the multivariate result of each study to minimize publication bias, such as the inverse input of adjusted ORs and 95% CIs for included studies reporting only the prognostic effects of appropriate EAT. However, the publication bias in the adjusted analyses remained greater than that in the unadjusted analyses. We believe that this bias was partially caused by the lack of included studies that found no significant impact of inappropriate EAT and those that reported their multivariate results only qualitatively, resulting in their true values of adjusted ORs being unavailable for our collection. Moreover, because of the publication bias, another leading limitation of the present study is the prognostic benefits of appropriate EAT only 293 disclosed in specific bacteraemic populations. Third, to avoid confusing the reader with a “massive” meta-analysis, only studies with mortality as the assessed outcome were included in our analyses. Therefore, information detailing the impacts of inappropriate EAT on the economic outcome, microbiological clearance rate, and hospitalization length was not presented herein.

Although current evidence cannot sufficiently support the disadvantage of inappropriate EAT in specific populations with bacteraemia, such as elder patients, non–ICU patients, causative *Enterobacter* species, and the source of urinary tract infections, this review and meta-analysis of contemporaneous literature demonstrates that inappropriate EAT is associated with unfavorable mortality outcomes overall and in most patient subgroups. Our findings underscore the necessity of precision medicine for the rapid diagnosis and for “treating the right patient with the right drug at the right time.”

## Data Availability Statement

The raw data supporting the conclusions of this article will be made available by the authors, without undue reservation.

## Author Contributions

C-CL executed the main database searches and helped to extract data from individual studies by using prespecified methods determined by all study authors and drafted this manuscript. C-CL and Y-PH independently reviewed 416 studies and helped to capture data from individual studies by using prespecified methods determined by all authors. W-CK revised it carefully from a professional point of view. All authors contributed to the inception of the research question, study design, read, and approved the final manuscript.

## Funding

This study was partially supported by research grants from the Ministry of Science and Technology (MOST 110-2314-B-006-068), the Ministry of Health and Welfare (MOHW109-TDU-B-211-114003), and National Cheng Kung University Hospital (NCKUH-11003036 and NCKUH-11104005), Tainan, Taiwan.

## Conflict of Interest

The authors declare that the research was conducted in the absence of any commercial or financial relationships that could be construed as a potential conflict of interest.

## Publisher's Note

All claims expressed in this article are solely those of the authors and do not necessarily represent those of their affiliated organizations, or those of the publisher, the editors and the reviewers. Any product that may be evaluated in this article, or claim that may be made by its manufacturer, is not guaranteed or endorsed by the publisher.
