# Supplementary material for: Effects of Inappropriate Administration of Empirical Antibiotics on Mortality in Adults With Bacteraemia: Systematic Review and Meta-Analysis
Source: Front Med (Lausanne). 2022 May 30;9:869822. doi: 10.3389/fmed.2022.869822 (PMC9197423; doi:10.3389/fmed.2022.869822)

Supplemental Table 1. Search strings for respective databases (Search date: 2021/7/29).

Research step	Query	Study number
PubMed (MEDLINE)		
1	antibiotic [MeSH] OR antimicrobial [MeSH] OR antibiotic [Title/Abstract] OR antimicrobial [Title/Abstract] OR antibiotic [Title/Abstract] OR antimicrobial [Title/Abstract]	942,233
2	appropriate [MeSH] OR inappropriate [MeSH] OR adequate [MeSH] OR inadequate [MeSH] OR appropriate [Title/Abstract] OR inappropriate [Title/Abstract] OR adequate [Title/Abstract] OR inadequate [Title/Abstract]	964,057
3	empirical [MeSH] OR initial [MeSH] OR empirical [Title/Abstract] OR initial [Title/Abstract]	959,975
4	bacteremia [MeSH] OR bacteraemia [MeSH] OR bloodstream [MeSH] OR bacteremia [Title/Abstract] OR bacteraemia [Title/Abstract] OR bloodstream [Title/Abstract]	320,688
5	mortality [MeSH] OR fatality [MeSH] OR death [MeSH] OR dead [MeSH] OR alive [MeSH] OR survival [MeSH] OR mortality [Title/Abstract] OR fatality [Title/Abstract] OR death [Title/Abstract] OR dead [Title/Abstract] OR alive [Title/Abstract] OR survival [Title/Abstract]	2,531,989
6	Children[Title/Abstract] OR neonate[Title/Abstract] OR adolescent[Title/Abstract] OR infant[Title/Abstract] OR pediatric[Title/Abstract]	1.586,707
7	#1 AND #2 AND #3 AND #4 AND #5 NOT #6	579
Cochrane		
1	MeSH descriptor: [antibiotic OR antimicrobial] explode all text	1,644
2	MeSH descriptor: [empirical OR initial OR appropriate OR inappropriate OR adequate OR inadequate] explode all text	8,206
3	MeSH descriptor: [bacteremia OR bacteraemia OR bloodstream] explode all text	407
4	MeSH descriptor: [mortality OR fatality OR death OR dead OR alive OR survival] explode all text	6,295
5	MeSH descriptor: [children OR neonate OR adolescent OR infant OR pediatric] explode title, abstract and keyword	2,813
6	#1 AND #2 AND #3 AND #4 NOT #5	204
Embase		
1	“antibiotic”/all fields OR “antimicrobial” /all fields	935,364
2	“appropriate” /all fields OR “inappropriate” /all fields OR “adequate” /all fields OR “inadequate” /all fields	134,870
3	“empirical”/all fields OR “initial”/all fields	1,294,873
4	“bacteremia" /all fields OR “bacteraemia”/all fields OR “bloodstream” /all fields	14,662
5	“mortality" /all fields OR “fatality” /all fields OR “death” /all fields OR “dead” /all fields OR “alive”/all fields OR”	1,214,168

	survival"/all fields	
6	"children"/ti,ab OR "neonate"/ti,ab OR "adolescent"/ti,ab OR "infant"/ti,ab OR "pediatric"/ti,ab	2,026,479
7.	#1 AND #2 AND #3 AND #4 AND #5 NOT #6	963

Supplemental Table 2. All included studies.

Study and country	Multi-center/Prospective/cohort	Study population /Patient No.	Acquisition place/Bacteraemia source	Specific population	Microorganisms; antibiotic-R isolates (%)	Primary outcome, (mortality rate)	Cut-off timeline for EAT (after initial culture)	Patient No. (%) of appropriate EAT	OR (95% C.I.) of inappropriate EAT for mortality	AOR (95% C.I.) of inappropriate EAT for mortality	Quality (NOQ assessment)
Bodey et al, 1986; US [203]	No/no/yes	Hospital/621	-/overall	Hemato-oncology	*E. coli*	7-day crude, 28.8	0 h	559 (90.0)	ND	NA	5
Meyers et al, 1989; US [202]	No/no/yes	Hospital/100	-/overall	the elderly	All	In-hospital crude, 40.0	0 h	84 (84.0)	ND	NA	5
Phillips et al, 1990; UK [200]	No/no/yes	Hospital/1505	-/overall	Overall	All	In-hospital crude, 18.7	0 h	1346 (89.4)	ND	NA	5
Feldman et al, 1990; South African [201]	No/yes/yes	Hospital/47	-overall	Overall	*K. pneumoniae*	In-hospital crude, 55.3	0 h	41 (87.2)	ND	NA	4
Elhanan et al, 1997; Israel [199]	No/yes/yes	Hospital/134	Community/UTI	Overall	All	In-hospital crude, 12.7	0 h	124 (92.5)	0.63 (0.08-5.24)	ND, NS	6
Salonen et al, 1998; Finland [196]	No/no/yes	Hospital/57	-/overall	Overall	Anaerobes	In-hospital crude, 24.6	BC result	28 (49.1)	ND	NA	5
Leibovici et al, 1998; Israel [197]	No/yes/yes	Hospital/3413	-/overall	Overall	All	In-hospital crude, 25.4	48h	2158 (63.2)	2.1 (1.8-2.4)	1.6 (1.3-1.9)	7
Carratala et al, 1998; Spain [198]	No/yes/yes	Hospital/40	-/pneumonia	Hemato-oncology	All	30-day crude, 62.5	BC result	35 (87.5)	ND	NA	6
Byl et al, 1999; Belgium [195]	No/yes/yes	Hospital/428	-/overall	Overall	All	In-hospital crude, 19.9	24h	269 (62.9)	ND	0.47 (0.25-0.87)	7
Soriano et al, 2000; Spain [193]	No/no/yes	Hospital/908	-/overall	Overall	*S. aureus*; MRSA (24.8)	7-day sepsis, 12.1	BC result	719 (79.2)	ND	2.13 (1.21-3.75)	7
Ibrahim et al, 2000; US [194]	No/yes/yes	ICU/492	-/overall	Overall	All	In-hospital crude, 38.4	BC result	345 (70.1)	2.18 (1.77-2.69)	6.86 (5.09-9.24)	6
Hanon et al, 2002; Denmark [192]	No/no/yes	Hospital/2058	-/overall	Overall	All	180-day crude, 30.6	0 h	1290 (62.7)	ND	0.64 (0.48-0.84)	7
Kang et al, 2003; South Korea [190]	No/no/yes	Hospital/136	-/overall	Overall	*P. aeruginosa*	30-day crude, 39.0	24h	47 (34.6)	2.14 (1.00-4.58)	4.61 (1.18-18.09)	7
Chamot et al, 2003; Switzerland [191]	No/no/yes	Hospital/115	-/overall	Overall	*P. aeruginosa*	30-day crude, 13.9	24h	98 (85.2)	1.1 (0.29-4.5)	1.2 (0.29-5.2)	8
Zaragoza et al, 2003; Spain [186]	No/yes/yes	ICU/166	-/overall	Overall	All	In-hospital crude, 51.8	BC result	127 (76.5)	ND	ND, NS	6
Valles et al, 2003; Spain [187]	Yes/yes/yes	ICU/339	Community/overall	Overall	All	in-hospital crude, 41.6	24h	290 (85.5)	ND	3.23 (1.52-6.83)	7
Endimiani et al, 2003; Italy [3]	No/no/yes	Hospital/521	-/overall	Overall	All	In-hospital crude, 11.3	BC result	314 (60.3)	ND	4.28 (0.68-72.08)	7
Lodise et al, 2003; US [189]	No/no/yes	Hospital/167	Nosocomial/overall	Overall	*S. aureus*; MRSA (61.7)	In-hospital sepsis, 23.4	44.75h	119 (71.3)	ND	3.8 (1.3-11.0)	7
Mackenzie et al, 2003; UK [188]	No/no/yes	Hospital/78	-/overall	Overall	All	In-hospital crude, 20.0	BC result	54* (83.1)	ND	NA	7
Lin et al, 2004; Taiwan [183]	No/no/yes	Hospital/86	Community/overall	Overall	*S. aureus*; MRSA (25.6)	14-day crude, 25.6	0 h	70 (81.4)	ND	NA	7
Anatoliotaki et al, 2004; Greece [185]	No/no/no	Hospital/157	-/overall	Oncology	All	In-hospital sepsis, 19.7	24h	111 (70.7)	ND	0.25 (ND)	7
Bouza et al, 2004; Spain [184]	No/yes/yes	Hospital/297	-/overall	Overall	All	In-hospital crude, 23.6	24h	170 (58.6)	0.5 (0.3-0.9)**	NA	6
Hung et al, 2005; Taiwan [181]	No/yes/yes	Hospital/52	Community/overall	Overall	Anaerobes	30-day crude, 25.0	72h	26* (74.3)	ND	NA	6
Bouza et al, 2005; Spain [182]	Yes/no/yes	Hospital/77	Nosocomial/overall	Overall	*S. pneumoniae*	In-hospital crude, 45.5	24h	65 (84.4)	ND	10.6 (1.2-97.0)	8
Shih et al, 2005; Taiwan [177]	No/no/yes	Hospital/71	-/overall	Overall	*S. marcescens*	14-day crude, 40.8	48h	30 (42.3)	ND	NA	7
Wang et al, 2005; Taiwan	No/no/yes	Hospital/371	-/overall	Hematology	All	In-hospital crude, 25.1	0 h	303 (81.7)	16.80 (8.60-33.15)	NA	6

[176]											
Micek,et al, 2005; US [178]	No/no/yes	Hospital/305	-/overall	Overall	*P. aeruginosa*	In-hospital crude, 21.0	0 h	230 (75.4)	ND	2.04 (1.42-2.92)	8
Kang et al, 2005; South Korea [180]	No/no/yes	Hospital/286	-/overall	Overall	GNB; 3rd GC-R (100)	30-day crude, 33.2	24h	135 (47.2)	1.65 (1.001-2.73)	ND,NS	8
Metan et al, 2005; Turkey [179]	No/no/yes	Hospital/53	-/overall	Overall	*E. coli*; ESBL (100)	30-day crude, 26.4	0 h	12 (22.6)	3.00 (0.86-10.41)	NA	7
Ye et al, 2006; China [167]	No/no/yes	Hospital/126	-/overall	Overall	*Enterobacter* spp.; MDR (15.2)	30-day crude, 39.7	5d	58 (46.0)	ND	ND, NS	7
Schramm et al, 2006; US [168]	No/no/yes	Hospital/559	-/overall	Overall	*S. aureus*; MRSA (100)	In-hospital crude, 23.1	24h	169 (30.2)	ND	1.92 (1.48-2.50)	8
Garey et al, 2006; US [173]	Yes/no/yes	Hospital/230	Nosocomial/overall	Overall	*Candida* spp.	In-hospital crude, 24.5	24h	130* (67.7)	ND	1.50 (1.09-2.09)	8
Falagas et al, 2006; Greece [175]	No/no/yes	Hospital/40	-/overall	Overall	*A. baumannii*	in-hospital crude, 47.5	72h	18 (45.0)	ND	NA	7
Marra et al, 2006; US [169]	No/no/yes	Hospital/91	-/overall	Overall	*S. aureus*; MRSA (46.2)	In-hospital crude, 14.3	24h	64 (70.3)	2.3 (0.70-7.75)	NA	7
Khatib et al, 2006; US [171]	No/yes/yes	Hospital/342	-/overall	Overall	*S. aureus*; MRSA (50.9)	In-hospital crude, 23.7	0 h	282 (82.5)	ND	ND, NS	7
Kim et al, 2006; South Korea [4]	No/no/yes	Hospital/238	-/overall	Overall	*S. aureus*; MRSA (53.4)	12-week sepsis, 33.2	48h	121 (50.8)	1.60 (0.93-2.76)	1.39 (0.62-3.15)	9
Fang et al, 2006; Taiwan [174]	No/no/yes	Hospital/162	Nosocomial/overall	Overall	*S. aureus*; MRSA (100)	30-day crude, 36.4	48h	43 (26.5)	ND	0.83 (0.66-1.04)	7
Lin et al, 2006; Taiwan [170]	No/no/yes	Hospital/108	-/overall	Overall	*E. cloacae*	In-hospital sepsis, 20.4	0 h	67 (62.0)	ND	NA	6
Guilarde et al 2006; Brazil [172]	No/no/yes	Hospital/111	-/overall	Overall	*S. aureus*; MRSA (60.2)	In-hospital sepsis, 35.1	48h	67 (60.4)	2.90 (1.44-5.84)	2.27 (1.02-5.09)	7
Peralta et al, 2007; Spain [162]	No/no/yes	Hospital/663	-/overall	Overall	*E. coli*; ESBL (3.3)	In-hospital crude, 5.4	24h	557 (84.0)	2.6 (1.34-5.03)	2.98 (1.25-7.11)	6
Ortega et al, 2007; Spain [164]	No/yes/yes	Hospital/200	Community/primary	Overall	All	In-hospital crude, 13.0	BC result	162 (81.0)	ND	2.0 (1.22-3.33)	7
Bassetti et al, 2007; Italy [166]	Yes/yes/yes	Hospital/136	-/overall	Overall	*Candida* spp.	21-day crude, 55.1	72h	87* (73.7)	4.89 (1.71-15.87)	4.75 (1.71-13.20)	6
Osih et al, 2007; US [163]	No/no/yes	Hospital/167	-/overall	Overall	*P. aeruginosa*	In-hospital crude, 36.5	24h	99 (59.3)	ND	NA	7
Lodise et al, 2007; US [165]	No/no/yes	Hospital/100	-/overall	Overall	*P. aeruginosa*	30-day crude, 31.0	52h	52 (52.0)	ND	4.1 (1.2-13.9)	6
Labelle AJ et al, 2008; US [160]	No/no/yes	Hospital/245	-/overall	Overall	*Candida* spp.	In-hospital crude, 45.7	24h	37 (15.1)	ND	NA	7
Cheong et al, 2008; South Korea [161]	No/no/yes	Hospital/614	Community/overall	Overall	*P. aeruginosa, E. coli*	30-day crude, 15.8	24h	508 (82.7)	ND	3.51 (1.17-10.52)	6
Tumbarello et al, 2008; Italy [155]	No/no/yes	Hospital/129	-/overall	Overall	*E. coli*; ESBL (100)	21-day crude, 29.4	BC result	73 (56.6)	7.63 (2.97-20.58)	6.22 (2.33-16.61)	6
Marcos et al, 2008; Spain [158]	No/no/yes	Hospital/377	-/overall	Overall	*Enterobacter* spp.	30-day crude, 12.5	BC result	232 (61.6)	ND	NA	6
Marschall et al, 2008; US [157]	No/yes/yes	Hospital/250	-/overall	Overall	GNB	In-hospital crude, 14.0	24h	171 (68.4)	ND	NA	7
Wareham et al, 2008; UK [154]	No/no/yes	Hospital/399	-/overall	Overall	*Acinetobacter* spp.; MDR (34.0)	30-day crude,6.3	BC result	88* (54.3)	ND	NA	7
Soriano et al, 2008; Spain [156]	No/yes/yes	Hospital/414	-/overall	Overall	*S. aureus*; MRSA (100)	30-day crude, 28.0	BC result	168 (40.6)	0.8 (0.5-1.3)**	NA	6
Lin et al, 2008; US [159]	No/no/yes	Hospital/1523	-/overall	Overall	All	30-day crude, 8.5	24h	983 (64.5)	ND	NA	9
Su et al, 2009; Taiwan [145]	No/no/yes	Hospital/123	-/overall	Overall	*Fusobacterium* spp.	In-hospital crude, 40.7	BC result	66 (53.6)	ND	NA	7
Erbay et al, 2009; Turkey [150]	No/no/yes	Hospital/103	Nosocomial/overall	Overall	*A. baumannii*	30-day crude, 54.4	48h	43 (41.7)	ND	2.4 (1.3-4.2)	8

Ammerlaan et al, 2009;Europe [153]	Yes/no/yes	Hospital/334	-/overall	Overall	*S. aureus*; MRSA (23.1)	30-day crude, 24.0	BC result	240 (71.9)	0.69 (0.36-1.32)	NA	7
Metan et al, 2009; Turkey [147]	No/yes/yes	Hospital/100	Nosocomial/overall	Overall	*A. baumannii*; MDR (48.0)	14-day crude, 63.0	24h	24 (24.0)	ND	ND, NS	8
Evans et al, 2009; US [149]	No/no/yes	Hospital/235	-/overall	Spinal cord injury	All	30-day crude, 6.8	48h	147 (62.6)	ND	1.74 (0.61-4.94)	8
Klevay et al, 2009; US [148]	Yes/yes/yes	Hospital/322	-/overall	Overall	*Candida* spp.	28-day crude, 29.8	0 h	244 (75.8)	ND	NA	6
Daikos et al, 2009; Greece [151]	Yes/yes/yes	Hospital/162	Nosocomial/overall	Overall	*K. pneumoniae*; MBL (41.4)	14-day crude, 19.1	48h	133 (82.1)	2.27 (0.91-5.64)	1.63 (0.68-3.86)	9
Ortega et al, 2009; Spain [146]	No/yes/yes	Hospital/4758	-/overall	Overall	*E. coli;* ESBL (4.4)	30-day crude, 9.2	BC result	4409 (92.7)	ND	4.83 (3.48-6.71)	7
Trecarichi et al, 2009; Italy [144]	No/no/yes	Hospital/62	-/overall	Hematology	*E. coli;* ESBL (41.9)	30-day crude, 21.0	48h	51 (82.3)	5.40 (2.25-12.96)	14.96 (1.95-114.51)	8
Chang et al, 2009; Taiwan [152]	No/no/yes	Hospital/88	-/overall	Overall	*Enterobacter aeruginosa*	In-hospital sepsis, 10.2	BC result	55 (62.5)	ND	ND, NS	6
Abhilash et al, 2010; India [143]	No/yes/yes	Hospital/131	-/overall	Overall	*E. coli, K. pneumoniae*; ESBL (73.3)	14-day crude, 23.6	0 h	74 (56.5)	0.76 (0.34-1.73)**	NA	6
Khan et al, 2010; Qatar [141]	Yes/yes/no	Hospital/452	-/overall	Overall	All	In-hospital crude, 22.5	24h	387 (85.6)	ND	8.35 (3.26-29.05)	7
Vitkauskiene et al, 2010; Lithuania [130]	No/no/yes	Hospital/80	-/overall	Overall	*P. aeruginosa*	30-day crude, 28.8	BC result	49 (61.3)	1.05 (0.42-2.61)**	NA	6
Paul et al, 2010; Israel [137]	No/no/yes	Hospital/510	-/overall	Overall	*S. aureus*; MRSA (100)	30-day crude, 43.9	48h	168 (32.9)	ND	2.15 (1.34 - 3.46)	8
Lin et al, 2010; Taiwan [140]	No/no/yes	Hospital/40	-/vascular catheter	Overall	*Chryseobacterium* spp.	14-day crude, 52.5	72h	17 (42.5)	4.19 (1.10-15.90)	ND, S	7
Tumbarello et al, 2010; Italy [131]	No/no/yes	Hospital/134	-/overall	Overall	*E. coli*; ESBL (27.6)	21-day crude, 12.7	0 h	107 (79.9)	3.09 (1.92-4.98)	NA	7
Schweizer et al, 2010; US [134]	No/no/yes	Hospital/814	-/overall	Overall	*S. aureus*; MRSA (60.0)	30-day crude, 22.9	24h	537 (66.0)	1.52 (0.99-2.34)	1.50 (0.96-2.35)	9
Son et al, 2010; South Korea [133]	Yes/yes/yes	Hospital/1144	Healthcare-associated/overall	Overall	All	30-day crude, 13.9	24h	930 (81.3)	ND	6.04 (2.1616.87)	8
Martinez et al, 2010; Spain [139]	No/yes/yes	Hospital/4863	-/overall	Overall	GNB	30-day crude, 9.6	BC result	4409 (90.7)	2.2 (1.7-2.9)	1.8 (1.3-2.5)	7
Tam et al, 2010; US [132]	No/no/yes	Hospital/109	-/overall	Overall	*P. aeruginosa*; MDR (22.9)	30-day crude, 18.3	24h	93 (85.3)	0.295 (0.093-0.943)**	ND, NS	8
Corona et al, 2010; UK [142]	Yes/yes/yes	ICU/1702	-/overall	Overall	All	In-ICU crude, 38.7	0 h	689 (40.5)	0.79 (0.58-1.07)**	0.89 (0.61-1.30)**	7
Rodriguez-Bano et al, 2010; Spain [136]	Yes/yes/yes	Hospital/96	Nosocomial/overall	Overall	*E. coli;* ESBL (100)	30-day crude, 30.2	24h	53 (55.2)	1.0 (0.5-1.8)	ND, NS	7
Micek et al, 2010; US [138]	No/no/yes	Hospital/760	-/overall	Overall	GNB	In-hospital crude, 47.0	24h	238 (31.3)	ND	2.30 (1.89-2.80)	8
Rodriguez-Bano J et al, 2010; Spain [135]	Yes/no/yes	Hospital/282	Community/overall	Overall	*E. coli*; ESBL (33.7)	14-day crude, 11.0	24h	205 (72.7)	3.3 (1.6-6.2)	3.0 (1.3-12.2)	7
Tuon et al, 2011; Brazil [110]	No/no/yes	Hospital/104	-/overall	Overall	*K. pneumoniae*; ESBL (58.7)	30-day crude, 46.2	48h	55 (52.9)	ND	NA	6
Rosa et al, 2011; Italy [127]	No/no/yes	Hospital/128	-/overall	Overall	*E. coli, K. pneumoniae, P. mirabilis*; ESBL (100)	21-day crude,17.2	72h	74 (81.3	ND	NA	7
Rebelo et al, 2011; Portugal [117]	No/no/yes	Hospital/135	-/overall	the elderly	All	In-hospital crude, 22.2	BC result	46 (34.1)	0.375 (0.139-1.010)**	ND, NS	8
Feodoroff et al, 2011;	Yes/no/yes	Population/76	-/overall	Overall	*Campylobacter*	30-day crude, 2.6	0 h	30 (39.5)	ND	NA	6

Finland [125]7					spp.							
Ku et al, 2011; South Korea [122]	No/no/yes	Hospital/27	-/overall	Overall	*Burkholderia cepacia*	28-day crude, 40.7	24h	19 (70.4)	ND	23.921 (1.313-435.967)	7
Park et al, 2011; South Korea [118]	No/no/yes	Hospital/150	Community/overall	Overall	*E. coli;* ESBL (23.3)	30-day crude, 11.3	24h	108 (72.0)	2.0 (0.7-5.5)	NA	7
Schechner et al, 2011; Israel [115]	Yes/yes/yes	Hospital/76	-/overall	Overall	*P. aeruginosa*	In-hospital crude, 35.5	24h	44 (57.9)	1.7 (0.9-3.2)	ND, NS	8
Wang et al, 2011; Taiwan [109]	No/no/yes	Hospital/113	-/overall	Overall	*E. coli, K. pneumoniae*; ESBL (100)	14-day crude, 23.9	BC result	50 (44.2)	ND	NA	7
Ortega et al, 2011; Spain [119]	No/yes/yes	Hospital/910	-/overall	Overall	*K. pneumoniae*; ESBL (12.3)	30-day crude, 10.5	BC result	824 (90.5)	ND	ND, NS	7
Shime et al, 2011; Japan [114]	No/no/yes	Hospital/310	-/overall	Immunocompeten t	All	In-hospital crude, 7.1	24h	270 (87.1)	ND	NA	6
Johnson et al, 2011; US [123]	No/no/yes	Hospital/754	-/overall	Overall	GNB	In-hospital crude, 41.1	24h	519 (68.8)	ND	2.03 (1.66-2.49)	8
Asgeirsson et al, 2011; Iceland [129]	No/no/yes	Hospital/325	-/overall	Overall	*S. aureus*	30-day crude, 13.8	BC result	262 (80.6)	ND	NA	7
Reisfeld et al, 2011; Israel [116]	No/no/yes	Hospital/378	-/overall	Dementia	All	30-day crude, 38.6	48h	227 (60.1)	1.72 (1.13-2.62)	1.41 (0.86-2.29)	9
Song et al, 2011; South Korea [113]	No/no/yes	Hospital/28	-/overall	Overall	*A. baumannii*; carbapenem-R (100)	30-day crude, 53.6	48h	13 (46.4)	ND	2.1 (0.2-23.3)	7
Tumbarello et al, 2011; Italy [111]	Yes/no/yes	Hospital/106	-/overall	Overall	*P. aeruginosa*; MDR (37.7)	21-day crude, 34.0	BC result	69 (65.1)	3.89 (1.52-9.99)	2.73 (1.08-6.85)	7
Gudiol et al, 2011; Spain [124]	No/yes/no	Hospital/372	-/overall	Hemato-oncology	GNB; MDR (13.7)	30-day crude, 22.0	BC result	290* (83.1)	ND	1.57 (0.50-4.90)	7
Kang et al, 2011; South Korea [128]	Yes/no/no	Hospital/365	-/IAI	Overall	GNB; 3rd-GC-R (19.5)	30-day crude, 11.5	24h	301 (82.5)	ND	NA	7
Lewis et al, 2011; UK [120]	No/no/yes	Hospital/195	-/overall	Overall	*S. aureus*; MRSA (100)	30-day crude, 26.7	BC result	63 (32.3)	ND	NA	6
Enoch et al, 2011; UK [126]	No/yes/yes	Hospital/203	-/overall	Overall	GNB; ESBL (3.0)	30-day crude, 22.2	0 h	163 (80.3)	2.63 (1.09-6.34)	NA	7
Lee et al, 2011; South Korea [121]	No/no/yes	Hospital/164	Community/UTI	Overall	All	In-hospital crude, 1.2	BC result	135 (82.3)	0 (0-0)	NA	7
Takesue et al, 2011; Japan [112]	No/no/yes	Hospital/128	-/overall	Overall	*S. aureu*s; MRSA (100)	28-day crude, 34.4	BC result	22 (17.2)	ND	0.22 (0.06-0.91)**	7
Zarkotou et al, 2011; Greece [108]	No/yes/yes	Hospital/53	-/overall	Overall	*K. pneumoniae*; KPC (100)	In-hospital crude, 34.0	48h	14 (26.4)	1.11 (0.31-3.99)	NA	7
Horino et al, 2012; Japan [106]	No/no/yes	Hospital/134	-/overall	Overall	*P. aeruginosa*	30-day crude, 20.9	24h	71 (53.0)	ND	NA	6
Park et al, 2012; South Korea [98]	No/no/yes	Hospital/100	-/pneumonia	Overall	*P. aeruginosa*	28-day crude, 51.0	48h	65 (65.0)	ND	2.73 (1.11-6.71)	7
Peralta et al, 2012; Spain [97]	Yes/no/yes	Hospital/387	-/overall	Overall	*E. coli, K. pneumoniae;* ESBL (100)	In-hospital crude, 20.9	24h	198 (51.2)	0.69 (0.47-1.02)**	0.39 (0.31-0.97)**	7
Jung et al, 2012; South Korea [105]	Yes/no/yes	Hospital/553	Community/overall	Overall	*K. pneumoniae*; ESBL (3.3)	30-day crude, 17.5	BC result	513 (92.8)	2.46 (1.22-4.96)	2.43 (1.07-5.52)	8
Tumbarello et al, 2012; Italy [92]	Yes/no/yes	Hospital/125	-/overall	Overall	*K. pneumoniae*; KPC (100)	30-day crude, 41.6	0h	50 (40.0)	2.00 (1.129-3.34)	4.17 (1.61-10.76)	8
Morata et al, 2012; Spain [100]	No/yes/yes	Hospital/709	-/overall	Overall	*P. aeruginosa*; MDR (17.5)	30-day crude, 19.9	24h	463 (65.3)	ND	2.18 (1.215-3.899)	7

Labelle A et al, 2012; US [104]	No/no/yes	Hospital/906	-/overall	Overall	All	In-hospital crude, 60.2	24h	436 (48.1)	ND	NA	7
Ortega et al, 2012; Spain [99]	No/yes/yes	Hospital/1373	-/BTI	Overall	All	30-day crude, 9.2	BC result	1242 (90.5)	ND	1.4 (1.1-1.7)	7
Lee CC et al, 2012; Taiwan [103]	No/no/yes	ED/454	-/overall	Overall	All	28-day crude, 9.3	0h	330 (72.7)	1.36 (1.0-1.8)	2.26 (1.01-5.13)	8
Sancho et al, 2012; Spain [94]	No/yes/yes	ICU/371	Nosocomial/overall	Overall	All	In-hospital crude, 59.3	BC result	216 (58.2)	ND	1.93 (0.96 - 3.86)	7
Wu et al, 2012; Taiwan [91]	No/yes/yes	Hospital/62	-/overall	Overall	*E. coli*; ESBL (100)	30-day crude, 21.0	48h	38 (61.3)	1.61 (0.50-5.24)	NA	6
Retamar et al, 2012; Spain [96]	Yes/yes/yes	Hospital/801	-/overall	Overall	All	14-day crude, 18.5	BC result	602 (75.2)	1.47 (1.10-2.02)	2.12 (1.34-3.34)	7
Bassetti et al, 2012; Italy [107]	Yes/yes/yes	Hospital/165	-/overall	Overall	*S. aureus*; MRSA (53.9)	30-day crude, 21.2	BC result	109 (66.1)	4.07 (1.74-9.60)	2.79 (1.19-6.52)	7
Lye et al, 2012; Singapore [101]	Yes/no/no	Hospital/675	Nosocomial/overall	Overall	GNB; MDR (44.6)	30-day crude, 22.2	24h	381 (56.4)	0.67 (0.46-0.96)**	0.87 (0.55-1.38)**	8
Lee YT et al, 2012; Taiwan [102]	No/no/yes	Hospital/252	-/overall	Overall	*A. baumannii*	14-day crude, 29.8	48h	91 (36.1)	0.24 (0.12-0.47)**	0.22 (0.10-0.50)**	7
Rong et al, 2012; China [95]	No/no/yes	Hospital/118	Nosocomial/overall	Overall	*S. aureus*; MRSA (63.5)	In-hospital crude, 28.0	48h	72 (61.0)	ND	0.25 (0.09-0.69)**	7
Tumbarello M et al, 2012, Italy [93]	No/no/yes	Hospital/99	-/overall	Overall	*Proteus* spp.; MDR (36.4)	21-day crude, 30.3	BC result	78 (78.8)	14.62 (4.10-57.81)	9.85 (2.67-36.25)	7
Kuo et al, 2013; Taiwan [82]	No/no/yes	Hospital/256	Nosocomial/overall	Overall	*Acinetobacter* spp.	14-day crude, 9.4	48h	135 (50.8)	0.89 (0.39-2.02)**	NA	6
Horcajada et al, 2013; Spain [85]	Yes/yes/yes	Hospital/667	-/UTI	Overall	All	30-day crude, 10.2	24h	540 (81.0)	1.2 (0.6-2.2)	NA	6
Yang et al, 2013; Taiwan [75]	No/no/yes	Hospital/222	-/overall	Overall	All	In-hospital crude, 46.8	24h	91 (41.0)	2.250 (1.297-3.904)	3.715 (1.736-7.948)	7
Gudiol et al, 2013; Spain [86]	No/yes/yes	Hospital/92	-/overall	Hemato-oncology	*Enterococcus faecium*; VRE (0)	30-day crude, 28.3	BC result	59 (64.1)	ND	NA	6
Retamar et al, 2013; Spain [5]	Yes/yes/yes	Hospital/341	-/overall	Overall	All	30-day crude, 20.8	24h	284 (83.3)	1.6 (1.0-2.5)	2.00 (0.95-4.34)	7
Bang et al, 2013; South Korea [90]	No/no/yes	Hospital/61	-/overall	Overall	*P. aeruginosa*	30-day crude, 19.7	24h	18 (29.5)	ND	NA	6
Ortega et al, 2013; Spain [79]	No/yes/yes	Hospital/1007	-/UTI	Overall	All	30-day crude, 9.1	BC result	643* (71.9)	ND	1.86 (1.48-2.44)	7
Ruiz-Giardin et al, 2013; Spain [76]	No/no/yes	Hospital/323	-/overall	Overall	All	30-day crude, 11.8	BC result	226* (76.1)	2 (1.01-4.25)	NA	6
Frakking et al, 2013; Netherland [89]	Yes/no/yes	Hospital/232	-/overall	Overall	*E. coli*, *K. pneumoniae*, *Enterobacter* spp.; ESBL (100)	30-day crude, 19.9	24h	85 (36.6)	ND	NA	6
Pena et al, 2013; Spain [77]	Yes/yes/yes	Hospital/593	-/overall	Overall	*P. aeruginosa*	30-day crude, 29.7	24h	332 (56.0)	1.72 (1.02-2.90)	1.70 (0.99-2.92)	7
Kang, Wi et al, 2013; South Korea [83]	No/no/yes	Hospital/92	Community/overall	Overall	*E. coli*; ESBL (100)	30-day crude, 10.9	0 h	28 (30.4)	ND	NA	6
Gasch et al, 2013; Spain [87]	Yes/yes/yes	Hospital/579	-/overall	Overall	*S. aureus*; MRSA (100)	2-day crude, 8.5	48h	378 (65.3)	4.33	3.59 (1.63-7.89)	7
Chen et al, 2013; Taiwan [9]	No/no/yes	ED/937	Community/overall	Overall	All	30-day crude, 17.0	24h	682 (72.8)	ND	1.77 (1.29-2.44)	7
Kang et al, 2013; South Korea [84]	No/no/yes	Hospital/556	-/BTI	Overall	All	30-day crude, 8.8	24h	299 (53.8)	ND	2.25 (1.13-4.48)	7
Molina et al, 2013; Spain [80]	No/yes/yes	Hospital/210	Nosocomial/overall	Overall	All	30-day crude, 9.5	0 h	65 (31.0)	ND	ND, NS	7
Garnacho-Montero et al, 2013; Spain [88]	No/yes/yes	Hospital/188	-/vascular catheter	Overall	Candida spp.	In-hospital crude, 36.7	48h	66 (35.1)	0.66 (0.37-1.20)**	0.40 (0.23-0.83)**	7
Park et al, 2013; South	Yes/no/yes	Hospital/149	Nosocomial/overall	Overall	*P. aeruginosa, A.*	14-day crude, 29.5	72h	73 (49.0)	ND	0.23 (0.07-0.78)**	7

Korea [78]					*baumannii*						
Lee et al, 2013; Taiwan [81]	No/no/yes	Hospital/130	-/overall	Overall	*A. baumannii*; carbapenem-R (20.8)	30-day crude, 40.8	0 h	67 (51.5)	ND	0.31 (0.15-0.72)**	6
Zilberberg et al, 2014; US [63]	No/no/yes	Hospital/1064	-/overall	Overall	GNB; MDR (5.9)	In-hospital crude, 29.2	24h	819 (77.0)	ND	3.872 (2.770-5.413)	8
Shorr et al, 2014; US [64]	No/no/yes	ICU/131	-/overall	Overall	*A. baumannii;* carbapenem-R (58.0)	In-hospital crude, 49.6	24h	38 (29.0)	ND	1.418 (1.099-1.583)	8
Falcone et al, 2014; Italy [71]	No/yes/yes	Hospital/94	-/overall	Overall	*E. coli*, *K. pneumoniae*, *Enterobacter* spp.; ESBL (100)	21-day crude, 37.2	72h	52 (55.3)	ND	NA	6
Rodriguez-Pardo et al, 2014; Spain [65]	No/yes/yes	Hospital/291	Nosocomial/vascular catheter	Overall	All	30-day crude, 14.5	48h	157 (54.0)	0.43 (0.21-0.86)**	0.36 (0.17-0.77)**	7
Hernandez et al, 2014; Spain [69]	No/yes/yes	Hospital/745	Community/primary	Overall	All	30-day crude, 13.4	24h	556 (74.6)	ND	NA	6
Davis et al, 2014; Australia [72]	No/yes/yes	Hospital/41	Community/pneumonia	Overall	*A. baumannii*	28-day crude, 9.8	24h	41 (100)	ND	NA	6
Marin et al, 2014; Spain [66]	No/yes/yes	Hospital/528	-/overall	Oncology	All	30-day crude, 30.9	BC result	114 (21.6)	ND	NA	6
Bartoletti et al, 2014; Italy [74]	No/no/no	Hospital/162	-/overall	Liver cirrhosis	All	30-day crude, 29.0	24h	100 (61.7)	ND	2.92 (1.50-5.41)	8
Kim et al, 2014; South Korea [68]	No/no/yes	Hospital/234	-/overall	Overall	P. aeruginosa; MDR (2.6)	14-day crude, 22.2	24h	196 (83.8)	ND	NA	7
Lee et al, 2014; Taiwan [67]	No/no/yes	ICU/298	Nosocomial/overall	Overall	*A. baumannii;* MDR (31.5)	30-day crude, 33.6	48h	177 (59.4)	9.91 (5.66-17.36)	7.06 (3.59-13.87)	7
Bodro et al, 2014; Spain [73]	No/yes/yes	Hospital/1148	-/overall	Hemato-oncology	All	30-day crude, 22.0	BC result	880 (75.7)	ND	NA	6
Girometti et al, 2014 Italy [70]	No/no/yes	Hospital/217	-/overall	Overall	*K. pneumoniae*; ESBL (23.0)	30-day crude, 26.7	BC result	93 (42.9)	ND	1.9 (1.1-3.4)	7
Al-Dorzi et al, 2015; Saudi Arabia [62]	No/no/yes	ICU/60	Nosocomial/overall	Overall	*A. baumannii*	In-hospital crude, 90.0	BC result	36 (60.0)	ND	NA	6
Lee et al, 2015; Taiwan [57]	No/no/yes	Hospital/189	-/overall	Overall	*S. aureus*; MRSA (100)	30-day crude, 29.1	48h	148 (78.3)	9.91 (5.66-17.36)	6.78 (2.61-17.60)	7
Picot-Gueraud et al, 2015; France [54]	No/no/yes	Hospital/228	-/overall	Overall	Enterobacteriaceae; MDR (100)	30-day crude, 23.0	24h	137 (60.1)	ND	NA	6
Boel et al, 2015; Denmark [6]	No/no/yes	Hospital/2008	-/overall	Overall	All	30-day crude, 19.5	24h	1702 (84.8)	0.65 (0.51-0.83)**	0.79 (0.62-1.01)**	7
Park H et al, 2015; South Korea [56]	No/no/yes	Hospital/102	-/overall	Liver cirrhosis	All	30-day crude, 30.4	0 h	54 (52.9)	2.00 (0.97-4.13)	3.24 (1.50-7.00)	8
Park SY et al, 2015; South Korea [55]	No/no/yes	Hospital/109	-/overall	Overall	Coagulase(-) staphylococci; methicillin-R (86.2)	30-day crude, 23.9	48h	32 (29.4)	0.59 (0.25-1.35)	NA	7
Wu et al, 2015; China [53]	No/no/yes	Hospital/482	Nosocomial/overall	Overall	All	28-day crude, 16.8	48h	157 32.6)	0.27 (0.14-0.54)**	0.23 (0.10-0.56)**	8
Hsieh et al, 2015; Taiwan [58]	No/no/yes	ED/246	Community/overall	Liver cirrhosis	All	28-day crude, 23.4	72h	212 (86.2)	3.10 (1.46-6.60)	4.29 (1.65-11.16)	8
Cain et al, 2015; US [61]	Yes/no/yes	Hospital/390	-/overall	Overall	GNB	28-day crude, 13.6	48h	359 (92.1)	2.94 (1.40-5.55)	3.54 (1.67-6.85)	7
Hernandez et al, 2015; Spain [59]	No/yes/yes	Hospital/2605	Community/overall	the elderly,	All	30-day crude, 11.4	24h	2201 (84.5)	1.70 (1.26-2.28)	1.49 (1.03-2.16)	7
Dimopoulos et al, 2015; Greece [60]	Yes/yes/yes	ICU/288	Nosocomial/overall	Overall	All	28-day crude, 33.3	BC result	85 (29.5)	ND	0.48 (0.24-0.95)**	7

Ma et al, 2017; China [38]	No/no/yes	Hospital/168	Nosocomial/overall	Hematology	*E. coli*; ESBL (61.9)	30-day crude, 20.2	24h	129 (76.8)	0.881 (0.3012.581)**	NA	7
Trecarichi et al, 2016; Italy [45]	Yes/yes/yes	Hospital/278	Nosocomial/overall	Hemato-oncology	*K. pneumoniae*; KPC (57.9)	21-day crude, 36.3	0 h	126 (45.3)	5.11 (2.63-9.39)	1.87 (1.08-2.22)	8
Guillamet et al, 2016; US [48]	No/no/yes	Hospital/1031	-/pneumonia	Overall	All	In-hospital crude, 37.1	0 h	844 (81.9)	2.3 (1.7-3.2)	2.3 (1.6-3.2)	7
Yoon et al, 2016; South Korea [43]	Yes/yes/yes	Hospital/345	Healthcare-associated/overall	Overall	*S. aureus*; MRSA (100)	In-hospital crude, 33.0	48h	154 (44.6)	0.99 (0.63-1.56)**	NA	7
Gudiol et al, 2016; Spain [49]	No/no/yes	Hospital/54	-/pneumonia	Hemato-oncology	All	30-day crude, 46.3	BC result	46 (85.2)	ND	NA	6
Savage et al, 2016; Canada [46]	Yes/no/yes	ICU/1190	-/overall	Overall	All	In-hospital crude, 40.0	24h	924 (77.6)	ND	1.02 (0.70-1.48)	8
Yilmaz et al, 2016; Turkey [44]	Yes/yes/yes	Hospital/255	-/overall	Overall	*S. aureus*; MRSA (39.2)	28-day crude, 15.3	0 h	183 (71.8)	ND	NA	7
Calle et al, 2016; Spain [50]	No/no/yes	Hospital/98	-pneumonia	Overall	*S. aureus*; MRSA (42.9)	30-day crude, 46.9	24h	60 (61.2)	ND	NA	6
Abraham et al, 2016; US [52]	No/no/yes	ICU/55	Nosocomial/overall	Overall	All	In-hospital crude. 20.0	24h	43 (78.2)	ND	NA	6
Migiyama et al, 2016; Japan [47]	No/no/yes	Hospital/126	-/overall	Overall	*P. aeruginosa*	30-day crude, 31.7	48h	77 (61.1)	0.31 (0.17-0.59)**	0.21 (0.09-0.51)**	7
Cheng et al, 2016; Taiwan [51]	Yes/no/yes	Hospital/111	-/pneumonia	Overall	*E. coli* and *K. pneumoniae*; ESBL (100)	30-day crude, 40.5	5d	51 (45.9)	0.21 (0.09-0.49)**	0.19 (0.07-0.55)**	8
Yamaga & Shime, 2018; Japan [20]	No/no/yes	ICU/62	-/overall	Overall	All	60-day crude, 48.4	24h	46 (74.2)	0.19 (0.052-0.71)**	0.043 (0.0047-0.23)**	8
Saliba et al, 2018; Spain [21]	No/yes/yes	Hospital/546	Nosocomial/vascular catheter	Overall	All	30-day crude, 13.9	48h	331 (60.6)	ND	NA	6
Lee et al, 2017; Taiwan [40]	No/no/yes	ED/2349	Community/overall	Overall	All	28-day crude, 14.5	48h	1917 (81.6)	2.12 (1.63-2.75)	2.60 (1.88-3.60)	7
Xie et al, 2017; China [31]	No/no/yes	Hospital/852	-/overall	Liver cirrhosis	All	30-day crude, 22.5	24h	612 (71.8)	6.060 (4.282-8.576)	8.278 (5.46-12.55)	7
Li et al, 2017; China [39]	No/no/yes	Hospital/104	-/overall	Overall	*K. pneumoniae*; ESBL (39.4)	30-day crude, 25.0	BC result	79 (76.0)	ND	NA	6
Zhang et al, 2017; China [29]	No/no/yes	Hospital/226	Nosocomial/overall	Overall	Enterococci.; VRE (3.3)	30-day crude, 23.9	24h	213 (94.2)	0.2 (0.1-0.5)**	0.2 (0.1-0.4)**	8
Man et al, 2017; China [37]	No/no/yes	Hospital/853	-/overall	Overall	*K. pneumoniae*; ESBL (12.3)	30-day crude, 20.6	24h	489 (57.3)	ND	1.716 (1.267-2.324)	8
Tagashira et al, 2017; Japan [34]	Yes/no/yes	Hospital/573	-/BTI	Overall	All	30-day crude, 6.6	BC result	440 (76.8)	ND	2.78 (1.27-6.11)	7
Satlin et al, 2017; US [35]	Yes/no/yes	Hospital/121	-/overall	Overall	*E. coli*, *K. pneumoniae*, *Enterobacter* spp.; carbapenem-R (100)	30-day crude, 51.2	48h	55 (45.5)	ND	NA	6
Gradel et al, 2017; Denmark [41]	Yes/no/yes	Population/6483	-/overall	Overall	All	30-day crude, 15.7	BC result	3778* (74.5)	1.18 (1.00-1.02)	0.85 (0.70 - 1.02)	7
Wang X et al, 2017; China [32]	Yes/no/yes	Hospital/40	-/overall	Hematology	A. baumannii; carbapenem-R (32.5)	30-day crude, 32.5	48h	20 (50.0)	5.56 (12.50-25.00)	ND, NS	7
Adrie et al, 2017; France [42]	Yes/yes/yes	ICU/571	Nosocomial/overall	Overall	All	30-day crude, 35.7	24h	418 (73.2)	ND	2.3 (1.8-3.0)	7
Royo-Cebrecos et al, 2017; Spain [36]	No/yes/yes	Hospital/173	-/BTI	Oncology	All	30-day crude, 26.0	0 h	135 (78.0)	ND	NA	6
Wang W et al, 2017; China	No/no/yes	Hospital/138	-/overall	Overall	GNB; MDR (100)	In-hospital crude, 25.4	BC result	91 (65.9)	3.18 (1.44-7.04)	NA	6

33											
Yu et al, 2017; Taiwan [30]	No/no/yes	Hospital/49	-/overall	Overall	*K. pneumoniae;* ESBL (100)	In-hospital crude, 55.1	0 h	24 (49.0)	0.42 (0.18-0.95)**	0.88 (0.28-2.77)**	7
Park et al, 2018; South Korea [22]	No/no/yes	Hospital/146	-/pneumonia	Overall	*A. baumannii*; carbapenem-R (100)	28-day crude, 70.6	48h	42 (28.8)	2.3 (1.44-3.67)	3.24 (1.94 - 5.42)	8
Kuo SH, et al, 2018; Taiwan [24]	No/no/yes	ICU/428	Nosocomial/vascular catheter	Overall	All	14-day crude, 32.0	48h	153 (35.7)	ND	1.879 (1.011-3.493)	7
Bartoletti at al, 2018; Europe [28]	Yes/yes/yes	Hospital/312	-/overall	Liver cirrhosis	All	30-day crude, 25.3	24h	190 (60.9)	ND	3.14 (1.93-5.12)	7
Kleinhendler et al, 2018; Israel [25]	No/no/yes	Hospital/180	-/overall	Hemato-oncology	All	30-day crude, 22.8	BC result	65* (82.3)	4.6 (2.2-9.6)	NA	7
Haruki et al, 2018; Japan [26]	No/no/yes	ICU/101	-/overall	Overall	*E. coli;* ESBL (23.8)	28-day crude, 20.8	BC result	87 (86.1)	ND	NA	6
Liu et al, 2018; Taiwan [23]	No/no/yes	Hospital/36	-/overall	Overall	*Citrobacter freundii*	28-day crude, 16.7	48h	25 (69.4)	0.857 (0.132-5.552)	NA	5
Bassetti et al, 2018; Italy [27]	Yes/no/yes	Hospital/337	-/overall	the elderly	*S. aureus*; MRSA (39.2)	30-day crude, 35.7	BC result	70* (60.9)	ND	ND, NS	7
Lim et al, 2019; Australia [18]	No/no/no	Hospital/110	-/overall	Overall	*E. coli* and *K. pneumoniae;* ESBL (88.2)	30-day crude, 20.0	24h	53 (48.2)	ND	NA	6
Chusri et al, 2019; Thailand [19]	No/no/yes	Hospital/68	-/overall	Overall	*A. baumannii*	30-day crude, 41.7	BC result	26* (43.3)	0.12 (0.04-0.37)	0.05 (0.01-0.50)	8
Shargian-Alon et al, 2019; Israel [17]	No/no/yes	Hospital/46	Nosocomial/overall	Hematology	*A. baumannii*	7-day crude, 71.7	48h	22 (47.8)	ND	0.052 (0.005-0.590)**	7
Lee et al, 2019; Taiwan [7]	Yes/no/yes	ED/3194	Community/overall	Overall	All	30-day crude, 14.8	24h	2591 (81.1)	0.51 (0.41-0.64)**	0.62 (0.48–0.79)**	8
Alvarez-Marin et al, 2020; Spain [16]	Yes/yes/yes	Hospital/285	-/overall	Overall	*Enterobacter* spp.; ESBL (5.3)	30-day crude, 19.6	24h	168 (58.9)	ND	NA	6
Seo et al, 2020; South Korea [10]	No/no/yes	Hospital/133	-/overall	Overall	*E. coli* and *K. pneumoniae*; Carbapenem-R (100)	14-day crude, 24.1	48h	54 (40.6)	ND	NA	7
Martinez-Nadal et al, 2020; Spain [15]	Yes/yes/yes	Hospital/1615	-/overall	Hemato-oncology	All	30-day crude, 21.0	0 h	1221 (75.6)	ND	NA	6
Falcone et al, 2020; Italy [8]	Yes/no/yes	ICU/102	Nosocomial/overall	Overall	*K. pneumoniae*; KPC (100)	30-day crude, 45.1	24h	55 (53.9)	ND	NA	6
Babar et al, 2021; Pakistan [14]	No/yes/yes	Hospital/137	-/overall	Overall	GNB: Carbapenem-R (100)	14-day crude, 13.9	0 h	99 (72.3)	4.30 (1.87-9.89)	1.382 (1.132-1.687)	8

EAT = empirical antimicrobial therapy; AOR = adjusted odds ratio; BC = blood culture; CI = confidence interval; ESBL = extended-spectrum beta-lactamase; GC = generation cephalosporin; GNB = Gram-negative bacillus; ICU = intensive care unit; MRSA = methicillin-resistant S. aureus; MDR = multidrug resistant; NA = no analysis; ND = no data; NOQ = Newcastle-Ottawa Quality; NS = no significance; OR = odds ratio; R = resistant; S = significance; VRE = vancomycin-resistant enterococci

* indicated the different patient numbers between the entire study and patients assessed for prognostic effects of EAT.

** indicated the prognostic effect of appropriate EAT.

Supplemental Table 3. Included studies with other outcomes, specific sub-populations, or different EAT definitions.

Study and country	Aimed population, patient No.	Primary outcome, (mortality rate)	Cut-off timeline for EAT (after initial culture)	Patient No. (%) of appropriate EAT	OR (95% C.I.) of inappropriate EAT for mortality	AOR (95% C.I.) of inappropriate EAT for mortality
Byl et al, 1999; Belgium [195]	overall, 428	In-hospital sepsis, 13.0	24h	269, 62.9	ND	0.38 (0.17-0.83)
	septic shock, 47	In-hospital crude, 72.3	24h	36 (76.6)	ND	NA
	septic shock, 47	In-hospital sepsis, 61.7	24h	36 (76.6)	ND	NA
Bouza et al, 2004; Spain [184]	overall, 290	In-hospital sepsis, 17.6	24h	170, 58.6	0.5 (0.3-0.9)*	3.5 (1.5-8.2)
Falagas et al, 2006; Greece [175]	overall, 40	14-day crude, 37.5	72h	18, 45.0	ND	ND, NS
Fang et al, 2006; Taiwan [174]	overall, 162	30-day sepsis, 47.5	48h	43, 26.5	ND	0.99 (0.88-1.12)
Osih et al, 2007; US [163]	overall, 167	In-hospital crude, 36.5	48h	114, 68.2	ND	NA
Evans et al, 2009; US [149]	overall, 235	In-hospital crude, 11.1	48h	147, 62.6	ND	1.05 (0.43-2.55)
Horino et al, 2011; Japan [106]	overall, 134	30-day crude, 20.9	48h	81, 60.4	ND	NA
Schechner et al, 2011; Israel [115]	severe sepsis/septic shock, 22	In-hospital crude, 72.7	24h	14, 63.6	1.8 (1.1-2.8)	NA
Enoch et al, 2011; UK [126]	overall, 203	7-day crude, 12.0	0 h	163, 80.3	6.40 (2.22-18.45)	NA
Lee et al, 2011; South Korea [121]	overall, 164	Early clinical response at D3, 73.8	BC result	135, 82.3	8.79 (3.63-21.27)	11.08 (4.37-28.074)
		Longer LOS (>10day)	BC result	135 (82.3)	2.32 (1.02-5.31)	2.47 (1.04-5.84)
Labelle AJ et al, 2008; US [160]	overall, 245	In-hospital crude, 45.7	48h	100, 40.8	ND	ND, NS
Park et al, 2012; South Korea [98]	overall, 100	14-day crude, 30.0	48h	65, 65.0	ND	NA
		7-day crude, 21.0	48h	65 (65.0)	ND	NA
Retamar et al, 2012; Spain [96]	overall, 801	30-day crude 22.6	BC result	ND	ND	1.56 (1.01-2.40)
Retamar et al, 2013; Spain [5]	overall, 341	14-day crude, 16.4	24h	284, 83.3	2.0 (1.2-3.3)	3.33 (1.42-7.69)
Frakking et al, 2013; Netherland [89]	overall, 232	In-hospital crude, 23	24h	85, 36.6	ND	NA
Gasch et al, 2013; Spain [87]	overall, 579	30-day crude, 32	48h	382, 66.0	1.39 (1.04-1.37)	1.37 (1.02-1.83)
Molina et al, 2013; Spain [80]	overall, 105	30-day crude, 14.3	72h	60, 57.1	ND	NA
Marin et al, 2014; Spain [66]	overall, 528	48-hr crude, 6.8	0 h	408, 77.3	ND	NA
Al-Dorzi et al, 2015; Saudi Arabia [62]	overall, 60	ICU crude, 66.7	BC result	36, 60.0	ND	0.15 (0.03-0.96)*
Yamaga & Shime, 2018; Japan [20]	overall, 62	28-day crude, 38.7	24h	46, 74.2	0.54 (0.28-1.04)*	NA
		In-hospital crude, 45.2	24h	46 (74.2)	0.28 (ND)	NA
Zhang et al, 2017; China [29]	overall, 226	7-day crude, 11.1	24h	213, 94.2	0.0 (0.0-0.1)*	0.2 (0.0-0.7)*
Satlin et al, 2017; US [35]	overall, 121	30-day crude, 51.2	12h	19, 15.7	ND	NA
Gradel et al, 2017; Denmark [41]	overall, 5068	1-year crude, 20.6	BC result	3778, 74.5	1.54 (1.32-1.80)	1.35 (1.13-1.60)
Park et al, 2018; South Korea [22]	overall, 146	14-day crude, 65.8	5 day	42, 28.8	ND	NA
Kleinhendler et al, 2018; Israel [25]	overall, 79	7-day crude, 4.6	BC result	65, 82.3	9.3 (2.63-32.7)	NA
Bassetti et al, 2018; Italy[27]	overall, 115	7-day crude, 20.3	BC result	72, 62.6	ND	0.3 (0.1-0.9)*
Shargian-Alon et al, 2019; Israel [17]	overall, 46	7-day crude, 71.7	24h	11, 23.9	ND	NA
Lee et al, 2019; Taiwan [7]	overall, 3194	30-day crude, 14.8	48h	2719, 85.1	0.434 (0.343-0.548)*	0.54 (0.43–0.71)*
	critically ill, 646	30-day crude, 46.7	24h	526 (81.4)	0.564 (0.377-0.842)*	0.50 (0.33–0.78)*
	critically ill, 646	30-day crude, 46.7	48h	546 (84.5)	0.505 (0.327-0.781)*	0.42 (0.27–0.67)*
Martinez-Nadal et al, 2020; Spain [15]	*Pseudomonas* spp., 251	30-day crude, 34.7	0 h	201, 80.1	ND	3.02 (1.29-7.07)

EAT = empirical antimicrobial therapy;; AOR = adjusted odds ratio; BC = blood culture; CI = confidence interval; ICU = intensive care unit; LOS = length of hospital stay; OR = odds ratio; NA = no analysis; ND =no data; NS =no significance.

* indicated the prognostic effect of appropriate EAT.

Supplemental Figure 1. Unadjusted analyses in overall patients: the forest (1A) and funnel plots (1B).

(1A) Forest plots

Study or Subgroup	Inappropriate Events	Inappropriate Total	Appropriate Events	Appropriate Total	Weight	Odds Ratio M-H, Random, 95% CI
Abhilash 2010	13	47	17	74	0.5%	1.28 [0.55, 2.96]
Abraham 2016	3	12	8	43	0.2%	1.46 [0.32, 6.64]
Adrie 2017	77	153	127	418	0.7%	2.32 [1.59, 3.39]
Al-Dorzi 2015	21	24	33	36	0.2%	0.64 [0.12, 3.45]
Alvarez-Marin 2020	21	117	34	168	0.6%	0.86 [0.47, 1.58]
Ammerlaan 2009	20	94	60	240	0.6%	0.81 [0.46, 1.44]
Anatoliotaki 2004	18	46	14	111	0.5%	4.45 [1.97, 10.06]
Asgeirsson 2011	9	63	36	262	0.5%	1.05 [0.48, 2.30]
Babar 2021	16	38	3	99	0.3%	23.27 [6.23, 86.88]
Bang 2013	10	43	2	18	0.2%	2.42 [0.47, 12.39]
Bartoletti 2014	48	122	31	190	0.6%	3.33 [1.96, 5.65]
Bartoletti 2018	23	62	24	100	0.6%	1.87 [0.94, 3.72]
Bassetti 2007	25	31	40	87	0.4%	4.90 [1.83, 13.12]
Bassetti 2012	21	56	14	109	0.5%	4.07 [1.87, 8.88]
Bassetti 2018	21	45	20	70	0.5%	2.19 [1.00, 4.78]
Bodey 1986	50	62	129	559	0.6%	13.89 [7.18, 26.87]
Bodro 2014	66	268	187	880	0.7%	1.21 [0.88, 1.67]
Boel 2015	79	306	313	1702	0.8%	1.54 [1.16, 2.05]
Bouza 2004	37	120	33	170	0.6%	1.85 [1.08, 3.18]
Bouza 2005	10	12	25	65	0.2%	8.00 [1.62, 39.56]
Byl 1999	37	159	48	269	0.7%	1.40 [0.86, 2.26]
Cain 2015	9	31	44	359	0.5%	2.93 [1.27, 6.77]
Calle 2016	14	38	32	60	0.5%	0.51 [0.22, 1.17]
Carratala 1998	3	5	22	35	0.2%	0.89 [0.13, 6.02]
Chamot 2003	3	17	13	98	0.3%	1.40 [0.35, 5.55]
Chang 2009	6	33	3	55	0.3%	3.85 [0.89, 16.62]
Chen 2013	97	255	62	682	0.7%	6.14 [4.27, 8.83]
Cheng 2016	34	60	11	51	0.5%	4.76 [2.05, 11.02]
Cheong 2008	29	106	68	508	0.7%	2.44 [1.48, 4.01]
Chusri 2019	7	34	18	26	0.3%	0.12 [0.04, 0.37]
Corona 2010	415	1013	244	689	0.8%	1.27 [1.04, 1.55]
Daikos 2009	9	29	4	133	0.3%	14.51 [4.08, 51.60]
Davis 2014	0	0	21	41		Not estimable
Dimopoulos 2015	75	203	21	85	0.6%	1.79 [1.01, 3.16]
Elhanan 1997	0	10	17	124	0.1%	0.29 [0.02, 5.22]
Endimiani 2003	48	207	11	314	0.6%	8.32 [4.20, 16.46]
Enoch 2011	15	40	30	163	0.5%	2.66 [1.25, 5.65]
Erbay 2009	39	60	17	43	0.5%	2.84 [1.26, 6.38]
Evans 2009	8	88	8	147	0.4%	1.74 [0.63, 4.81]
Falagas 2006	13	22	6	18	0.3%	2.89 [0.79, 10.57]
Falcone 2014	15	52	20	42	0.5%	0.45 [0.19, 1.05]
Falcone 2020	30	47	16	55	0.5%	4.30 [1.87, 9.89]
Fang 2006	36	119	23	43	0.5%	0.38 [0.18, 0.77]
Feldman 1990	2	6	24	41	0.2%	0.35 [0.06, 2.16]
Feodoroff 2011	1	46	1	30	0.1%	0.64 [0.04, 10.71]
Frakking 2013	30	147	16	85	0.6%	1.11 [0.56, 2.17]
Garey 2006	24	62	23	130	0.6%	2.94 [1.49, 5.81]
Garnacho-Montero 2013	44	122	23	66	0.6%	1.05 [0.56, 1.97]
Gasch 2013	312	201	18	378		Not estimable
Girometti 2014	40	124	18	93	0.6%	1.98 [1.05, 3.75]
Gradel 2017	224	1290	571	3778	0.8%	1.18 [1.00, 1.40]
Gudiol 2011	20	59	62	290	0.6%	1.89 [1.03, 3.46]
Gudiol 2013	7	33	19	59	0.4%	0.57 [0.21, 1.54]
Gudiol 2016	6	9	19	46	0.3%	2.84 [0.63, 12.80]
Guilarde 2006	22	44	17	67	0.5%	2.94 [1.31, 6.59]
Guillamet 2016	100	187	282	844	0.7%	2.29 [1.66, 3.16]
Hanon 2002	279	768	351	1290	0.8%	1.53 [1.26, 1.85]
Haruki 2018	7	14	14	87	0.3%	5.21 [1.58, 17.20]
Hernandez 2014	28	189	72	556	0.7%	1.17 [0.73, 1.87]
Hernandez 2015	69	404	231	2201	0.8%	1.76 [1.31, 2.35]
Horcajada 2012	15	127	53	540	0.6%	1.23 [0.67, 2.26]
Horino 2011	14	63	14	71	0.5%	1.16 [0.51, 2.68]
Hsieh 2015	15	34	43	212	0.5%	3.10 [1.46, 6.60]
Hung 2005	2	9	4	26	0.2%	1.57 [0.24, 10.49]
Ibrahim 2000	91	147	98	345	0.7%	4.10 [2.73, 6.15]
Johnson 2011	125	235	185	519	0.7%	2.05 [1.50, 2.81]
Jung 2012	13	40	84	513	0.6%	2.46 [1.22, 4.96]
[illegible]	[illegible]	[illegible]	[illegible]	[illegible]	[illegible]	[illegible]

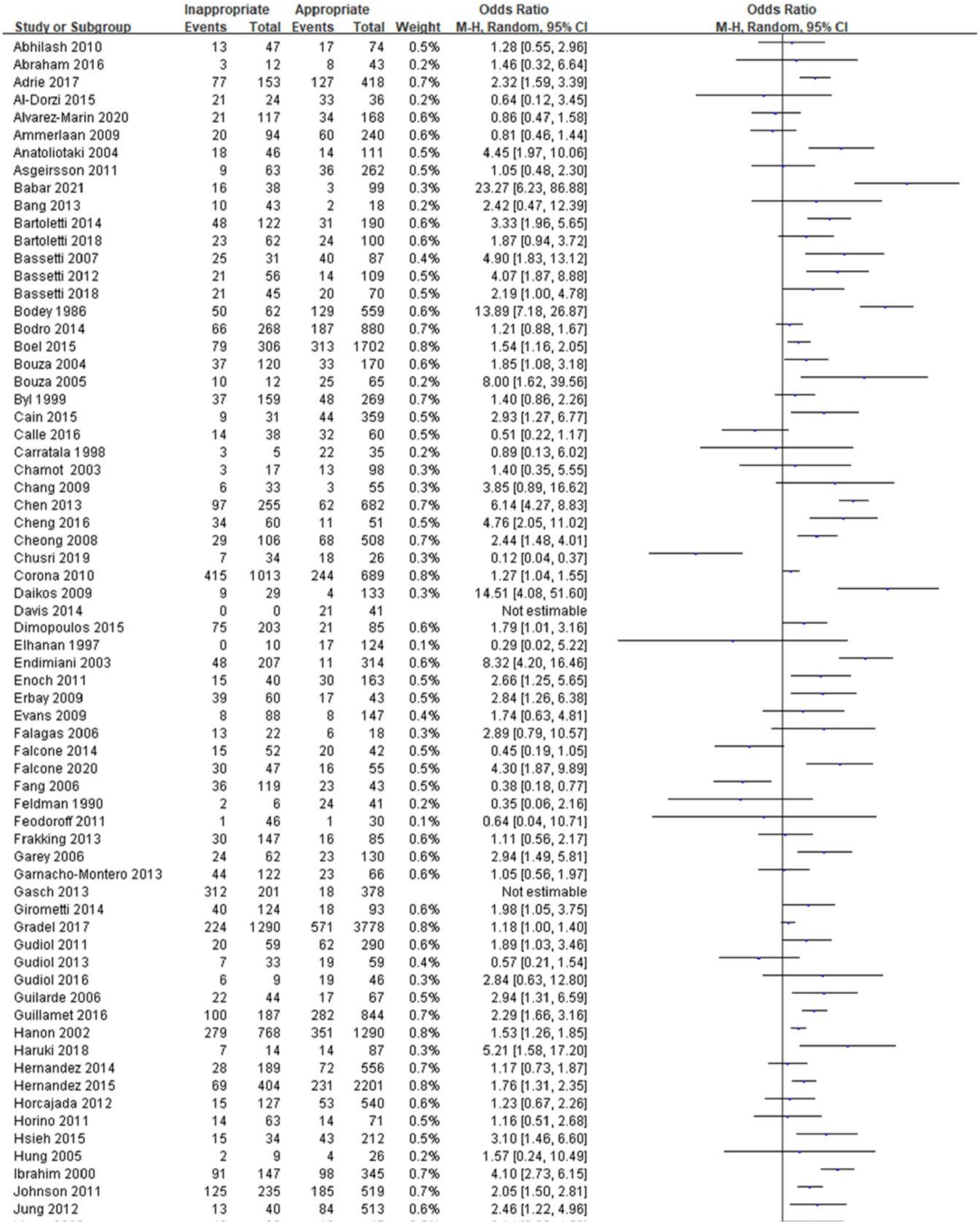

(1A) Forest plots (continued)

Kang 2003	40	89	13	47	0.5%	2.14 [0.99, 4.58]
Kang 2005	58	151	37	135	0.7%	1.65 [1.00, 2.72]
Kang 2011	10	64	32	301	0.5%	1.56 [0.72, 3.35]
Kang 2013	7	64	3	28	0.3%	1.02 [0.24, 4.28]
Kang Wi 2013	27	257	22	299	0.6%	1.48 [0.82, 2.66]
Khan 2010	55	65	47	387	0.5%	39.79 [18.99, 83.36]
Khatib 2006	21	60	60	282	0.6%	1.99 [1.09, 3.64]
Kim 2006	46	117	34	121	0.6%	1.66 [0.96, 2.85]
Kim 2014	7	38	45	296	0.5%	1.26 [0.52, 3.03]
Kleinhendler 2018	9	14	9	65	0.3%	11.20 [3.05, 41.10]
Klevay 2009	21	78	75	244	0.6%	0.83 [0.47, 1.47]
Ku 2011	6	8	5	19	0.2%	8.40 [1.26, 56.07]
Kuo 2012	13	131	12	135	0.5%	1.13 [0.50, 2.57]
Kuo SH 2018	100	275	37	153	0.7%	1.79 [1.15, 2.79]
Labelle A 2012	60	208	12	37	0.5%	0.84 [0.40, 1.79]
Labelle AJ 2012	321	470	224	436	0.8%	2.04 [1.56, 2.67]
Lee 2011	0	29	2	135	0.1%	0.91 [0.04, 19.35]
Lee 2013	32	63	21	67	0.5%	2.26 [1.11, 4.62]
Lee 2014	75	121	25	177	0.6%	9.91 [5.66, 17.36]
Lee 2015	24	41	31	148	0.5%	5.33 [2.55, 11.13]
Lee 2017	100	432	239	1917	0.8%	2.11 [1.63, 2.75]
Lee 2019	136	603	336	2591	0.8%	1.95 [1.56, 2.44]
Lee CC 2012	19	124	23	330	0.6%	2.42 [1.26, 4.61]
Lee NY 2012	63	177	12	91	0.6%	3.64 [1.84, 7.19]
Leibovici 1998	432	1255	436	2158	0.8%	2.07 [1.77, 2.43]
Lewis 2011	27	112	11	63	0.5%	1.50 [0.69, 3.28]
Li 2017	7	25	19	79	0.4%	1.23 [0.45, 3.39]
Lim 2019	10	57	12	53	0.4%	0.73 [0.28, 1.86]
Lin 2004	7	16	15	70	0.4%	2.85 [0.91, 8.92]
Lin 2006	12	41	10	67	0.4%	2.36 [0.91, 6.10]
Lin 2008	50	540	79	983	0.7%	1.17 [0.81, 1.69]
Lin 2010	17	23	6	17	0.3%	5.19 [1.33, 20.28]
Liu 2018	2	11	4	25	0.2%	1.17 [0.18, 7.56]
Lodise 2003	16	48	23	119	0.5%	2.09 [0.98, 4.43]
Lodise 2007	21	48	10	52	0.5%	3.27 [1.33, 7.99]
Lye 2012	77	294	73	381	0.7%	1.50 [1.04, 2.16]
Ma 2017	14	39	20	129	0.5%	3.05 [1.36, 6.86]
Mackenzie 2003	6	21	7	54	0.3%	2.69 [0.78, 9.24]
Man 2017	100	364	76	489	0.7%	2.06 [1.47, 2.88]
Marcos 2008	10	82	27	232	0.5%	1.05 [0.49, 2.29]
Marin 2014	123	414	40	114	0.7%	0.78 [0.50, 1.21]
Marra 2006	6	27	7	64	0.3%	2.33 [0.70, 7.72]
Marschall 2008	11	79	24	171	0.5%	0.99 [0.46, 2.14]
Martinez 2010	79	454	388	4409	0.8%	2.18 [1.68, 2.84]
Martinez-Nadal 2020	99	394	240	1221	0.8%	1.37 [1.05, 1.79]
Metan 2005	5	41	9	12	0.2%	0.05 [0.01, 0.23]
Metan 2009	53	76	10	24	0.4%	3.23 [1.25, 8.32]
Meyers 1989	8	16	32	84	0.4%	1.63 [0.55, 4.76]
Micek & Lioyd 2010	23	75	41	230	0.6%	2.04 [1.12, 3.70]
Micek 2010	270	522	87	238	0.7%	1.86 [1.36, 2.55]
Migiyama 2016	23	49	17	77	0.5%	3.12 [1.43, 6.80]
Molina 2013	1	13	0	18	0.1%	4.44 [0.17, 118.00]
Morata 2012	65	246	76	463	0.7%	1.83 [1.26, 2.66]
Ortega 2007	14	38	12	162	0.5%	7.29 [3.01, 17.64]
Ortega 2009	100	349	340	4409	0.8%	4.81 [3.72, 6.22]
Ortega 2011	14	86	82	824	0.6%	1.76 [0.95, 3.26]
Ortega 2012	20	131	106	1242	0.6%	1.93 [1.15, 3.24]
Ortega 2013	16	155	46	643	0.6%	1.49 [0.82, 2.72]
Osih 2007	26	68	35	99	0.6%	1.13 [0.60, 2.15]
Park 2011	7	42	10	108	0.4%	1.96 [0.69, 5.55]
Park 2012	24	35	27	65	0.5%	3.07 [1.29, 7.31]
Park 2013	17	76	17	73	0.5%	0.95 [0.44, 2.04]
Park 2018	80	104	23	42	0.5%	2.75 [1.29, 5.89]
Park H 2015	21	48	10	54	0.5%	3.42 [1.40, 8.35]
Park SY 2015	17	77	9	32	0.4%	0.72 [0.28, 1.85]
Paul 2010	168	342	56	168	0.7%	1.93 [1.31, 2.84]
Pena 2013	86	261	90	332	0.7%	1.32 [0.93, 1.88]
Peralta 2007	12	106	24	557	0.5%	2.84 [1.37, 5.86]
Peralta 2012	47	189	34	198	0.7%	1.60 [0.97, 2.62]
Phillips 1990	67	159	215	1346	0.7%	3.83 [2.71, 5.42]
Picot-Gueraud 2015	21	91	30	137	0.6%	1.07 [0.57, 2.02]
[illegible]	[illegible]	[illegible]	[illegible]	[illegible]	[illegible]	[illegible]

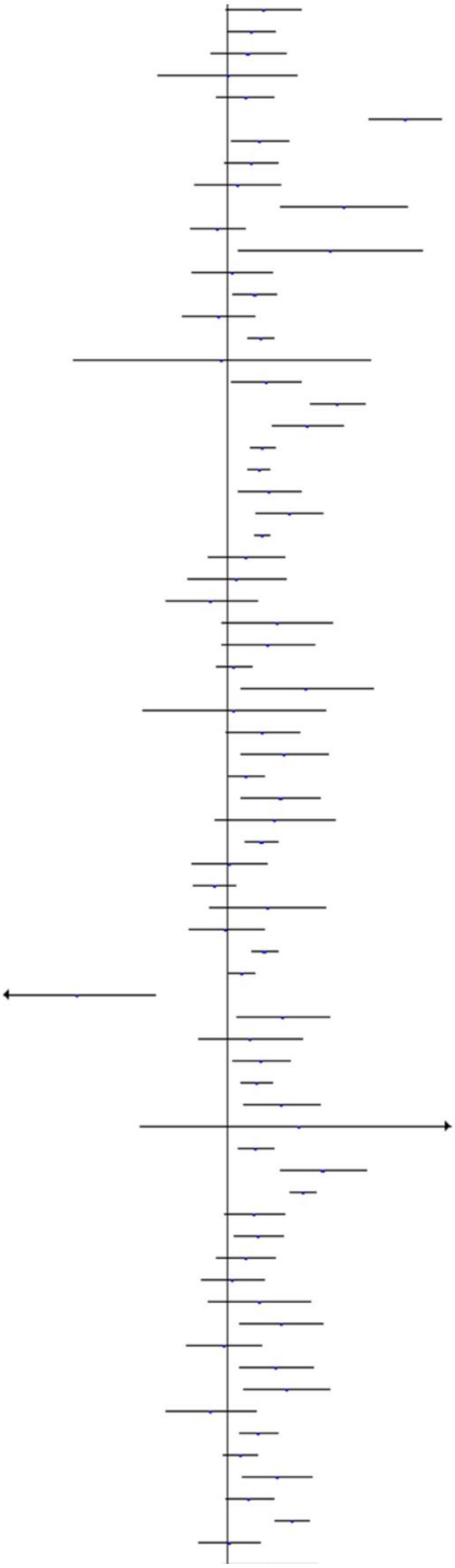

(1A) Forest plots (continued)

Rebelo 2011	24	89	6	46	0.4%	2.46 [0.93, 6.54]
Reisfeld 2011	70	151	79	227	0.7%	1.62 [1.06, 2.47]
Retamar 2012	49	199	99	602	0.7%	1.66 [1.13, 2.45]
Retamar 2013	17	57	53	284	0.6%	1.85 [0.98, 3.52]
Rodriguez-Bano 2010	13	43	16	53	0.5%	1.00 [0.42, 2.41]
Rodriguez-Bano J 2010	17	77	14	205	0.5%	3.87 [1.80, 8.30]
Rodriguez-Pardo 2014	22	134	15	157	0.6%	1.86 [0.92, 3.75]
Rong 2012	21	46	12	72	0.5%	4.20 [1.80, 9.82]
Rosa 2011	6	17	11	74	0.3%	3.12 [0.96, 10.20]
Royo-Cebrecos 2017	9	38	36	135	0.5%	0.85 [0.37, 1.98]
Ruiz-Giardin 2013	14	71	24	226	0.5%	2.07 [1.00, 4.25]
Saliba 2017	27	215	49	331	0.7%	0.83 [0.50, 1.37]
Salonen 1997	9	29	5	28	0.3%	2.07 [0.60, 7.20]
Sancho 2012	106	155	114	216	0.7%	1.94 [1.26, 2.98]
Satlin 2017	31	66	28	55	0.5%	0.85 [0.42, 1.75]
Savage 2016	135	266	341	924	0.8%	1.76 [1.34, 2.32]
Schechner 2011	15	32	14	44	0.4%	1.89 [0.74, 4.84]
Schramm 2006	99	390	28	169	0.7%	1.71 [1.08, 2.73]
Schweizer 2010	77	277	109	537	0.7%	1.51 [1.08, 2.12]
Seo 2020	16	79	16	54	0.5%	0.60 [0.27, 1.34]
Shargian-Alon 2019	22	24	11	22	0.2%	11.00 [2.07, 58.52]
Shih 2005	20	41	9	30	0.4%	2.22 [0.82, 5.99]
Shime 2011	6	40	16	270	0.4%	2.80 [1.03, 7.65]
Shorr 2014	54	93	11	38	0.5%	3.40 [1.51, 7.66]
Son 2020	77	214	116	930	0.7%	3.94 [2.81, 5.54]
Song 2011	11	15	1	13	0.1%	33.00 [3.18, 342.26]
Soriano 2000	33	189	77	719	0.7%	1.76 [1.13, 2.75]
Soriano 2008	73	246	43	168	0.7%	1.23 [0.79, 1.91]
Su 2007	23	57	27	66	0.5%	0.98 [0.47, 2.01]
Tagashira 2017	17	133	21	440	0.6%	2.92 [1.49, 5.72]
Takesue 2011	41	106	3	22	0.3%	3.99 [1.11, 14.35]
Tam 2010	6	16	14	93	0.3%	3.39 [1.06, 10.81]
Trecarichi 2009	7	11	5	51	0.2%	16.10 [3.46, 74.84]
Trecarichi 2016	79	152	22	126	0.6%	5.12 [2.92, 8.95]
Tumbarello 2008	29	56	9	73	0.5%	7.64 [3.19, 18.28]
Tumbarello 2010	11	27	6	107	0.4%	11.57 [3.75, 35.68]
Tumbarello 2011	20	37	16	69	0.5%	3.90 [1.66, 9.16]
Tumbarello 2012	39	75	13	50	0.5%	3.08 [1.42, 6.71]
Tumbarello 2013	16	21	14	78	0.4%	14.63 [4.59, 46.61]
Tuon 2011	20	49	28	55	0.5%	0.67 [0.31, 1.45]
Valles 2003	34	49	107	290	0.6%	3.88 [2.02, 7.45]
Vitkauskiene 2010	18	31	29	49	0.4%	0.95 [0.38, 2.38]
Wang 2005	50	68	43	303	0.6%	16.80 [8.96, 31.47]
Wang 2011	15	63	12	50	0.5%	0.99 [0.41, 2.36]
Wang W 2017	19	47	16	91	0.5%	3.18 [1.44, 7.04]
Wang X 2017	10	20	3	20	0.3%	5.67 [1.25, 25.61]
Wareham 2008	10	74	7	88	0.4%	1.81 [0.65, 5.01]
Wu 2012	9	38	4	24	0.3%	1.55 [0.42, 5.74]
Wu 2015	70	325	11	157	0.6%	3.64 [1.87, 7.10]
Xie 2017	81	240	111	612	0.7%	2.30 [1.64, 3.22]
Yamaga & Shime 2017	14	16	16	46	0.2%	13.13 [2.65, 65.07]
Yang 2013	72	131	32	91	0.6%	2.25 [1.30, 3.90]
Ye 2006	33	68	17	58	0.5%	2.27 [1.09, 4.76]
Yilmaz 2016	12	72	27	183	0.5%	1.16 [0.55, 2.43]
Yoon 2016	63	191	51	154	0.7%	0.99 [0.63, 1.56]
Yu 2017	16	25	11	24	0.4%	2.10 [0.67, 6.60]
Zaragoza 2003	22	39	64	127	0.5%	1.27 [0.62, 2.62]
Zarkotou 2011	13	39	5	14	0.3%	0.90 [0.25, 3.24]
Zhang 2017	13	13	41	213	0.1%	112.23 [6.54, 1926.54]
Ziberberg 2014	106	245	205	819	0.8%	2.28 [1.70, 3.08]
**Total (95% CI)**		**25954**		**64008**	**100.0%**	**2.06 [1.88, 2.25]**
Total events	8662		10815			

Heterogeneity: Tau² = 0.26; Chi² = 885.51, df = 195 (P < 0.00001); I² = 78%

Test for overall effect: Z = 15.63 (P < 0.00001)

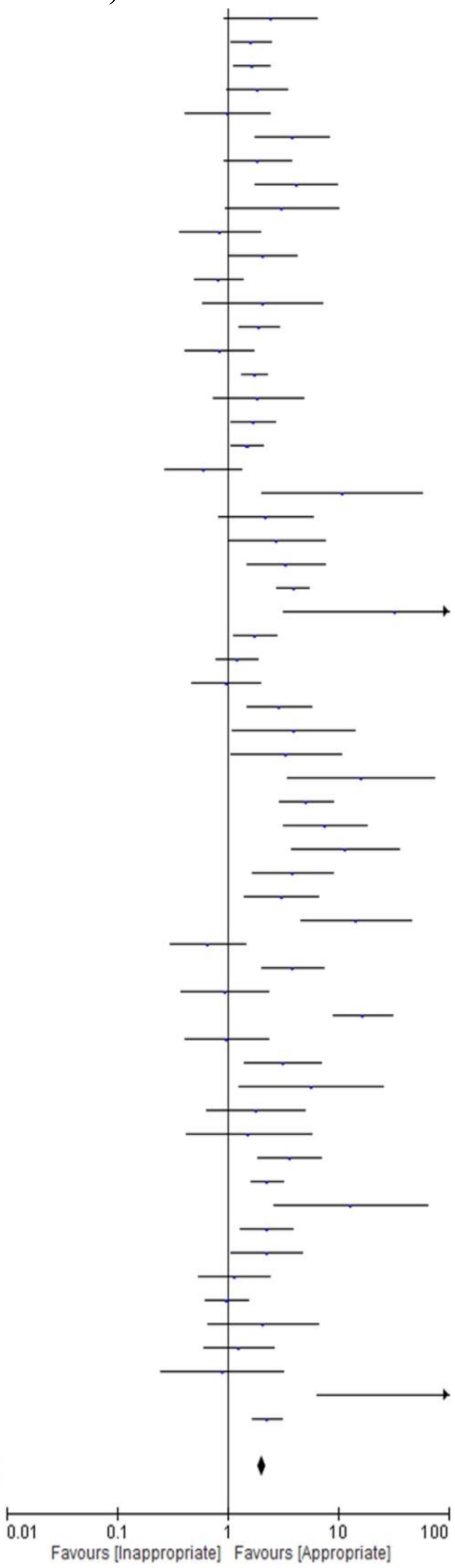

(1B) Funnel plots

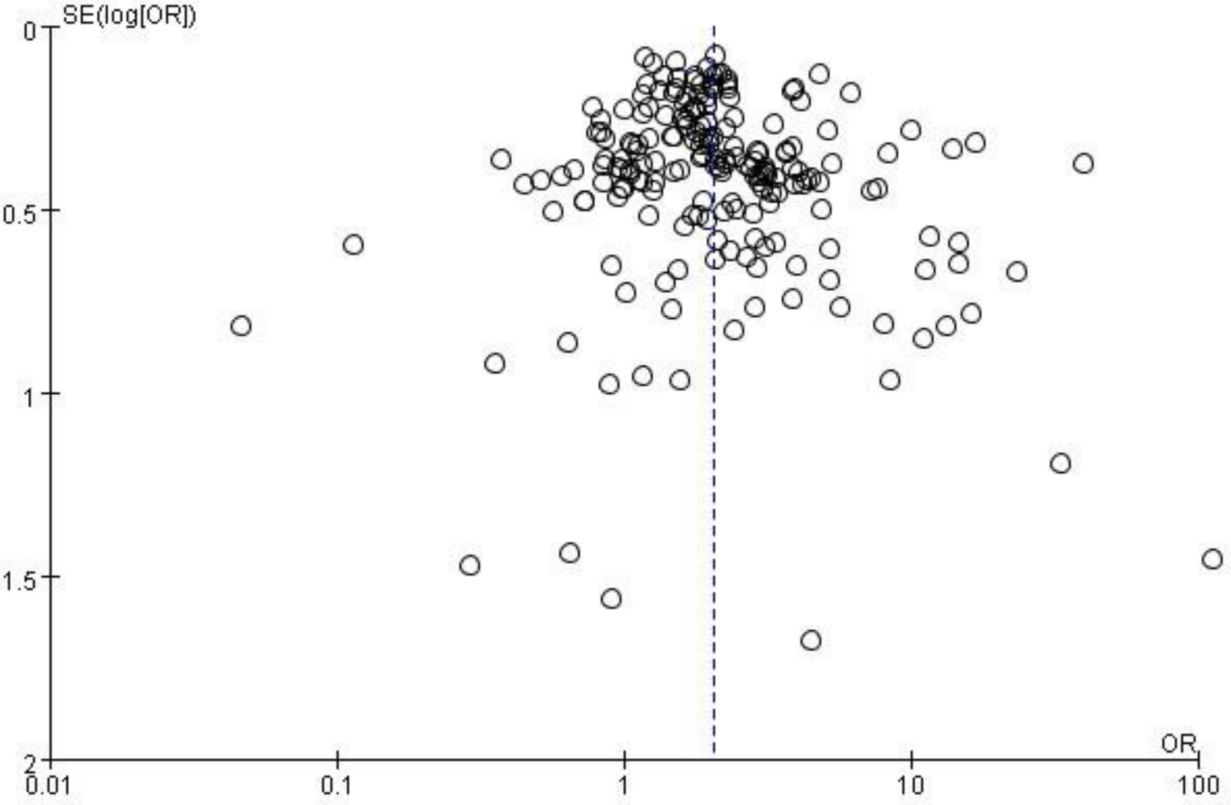

Supplemental Figure 2. Unadjusted analyses in subgroup patients: (2A) acquisition places; (2B) specific comorbidities or conditions; (2C) bacteraemia sources; (2D) bacteraemia severity; (2E) *Staphylococcus aureus*; (2F) Enterobacteriaceae, by acquisition places; (2G) Enterobacteriaceae, by species; (2H) glucose non-fermentative rods; (2I) antibiotic-resistant microorganisms; (2J) varied timeliness of empirical antimicrobial therapy (EAT); and (2K) varied mortality timeliness

(2A) Acquisition places

Study or Subgroup	Inappropriate Events	Inappropriate Total	Appropriate Events	Appropriate Total	Weight	Odds Ratio M-H, Random, 95% CI
**1.2.1 Community-onset/acquired**						
Chen 2013	97	255	62	682	2.9%	6.14 [4.27, 8.83]
Cheong 2008	29	106	68	508	2.7%	2.44 [1.48, 4.01]
Davis 2014	0	0	4	41		Not estimable
Elhanan 1997	0	10	17	124	0.4%	0.29 [0.02, 5.22]
Hernandez 2014	28	189	72	556	2.7%	1.17 [0.73, 1.87]
Hernandez 2015	67	404	231	2201	3.0%	1.70 [1.26, 2.28]
Hsieh 2015	15	34	43	212	2.1%	3.10 [1.46, 6.60]
Hung 2005	2	9	4	26	0.7%	1.57 [0.24, 10.49]
Jung 2012	13	40	84	513	2.2%	2.46 [1.22, 4.96]
Kang Wi 2013	7	64	3	28	1.1%	1.02 [0.24, 4.28]
Lee 2011	0	29	2	135	0.3%	0.91 [0.04, 19.35]
Lee 2017	100	432	239	1917	3.1%	2.11 [1.63, 2.75]
Lee 2019	136	603	336	2591	3.2%	1.95 [1.56, 2.44]
Lin 2004	7	16	15	70	1.5%	2.85 [0.91, 8.92]
Ortega 2007	14	38	12	162	1.9%	7.29 [3.01, 17.64]
Park 2011	7	42	10	108	1.6%	1.96 [0.69, 5.55]
Rodriguez-Bano J 2010	17	77	14	205	2.1%	3.87 [1.80, 8.30]
Valles 2003	34	49	107	290	2.3%	3.88 [2.02, 7.45]
**Subtotal (95% CI)**		**2397**		**10369**	**33.9%**	**2.49 [1.90, 3.27]**
Total events	573		1323			
Heterogeneity: Tau² = 0.17; Chi² = 57.90, df = 16 (P < 0.00001); I² = 72%						
Test for overall effect: Z = 6.57 (P < 0.00001)						
**1.2.2 Hospital-onset/acquired**						
Abraham 2016	3	12	8	43	1.0%	1.46 [0.32, 6.64]
Adrie 2017	77	153	127	418	2.9%	2.32 [1.59, 3.39]
Al-Dorzi 2015	21	24	33	36	0.9%	0.64 [0.12, 3.45]
Bouza 2005	10	12	25	65	0.9%	8.00 [1.62, 39.56]
Daikos 2009	9	29	22	133	1.8%	2.27 [0.91, 5.64]
Dimopoulos 2015	75	203	21	85	2.5%	1.79 [1.01, 3.16]
Erbay 2009	39	60	17	43	2.0%	2.84 [1.26, 6.38]
Falcone 2020	30	47	16	55	2.0%	4.30 [1.87, 9.89]
Fang 2006	36	119	23	43	2.2%	0.38 [0.18, 0.77]
Garey 2006	24	62	23	130	2.3%	2.94 [1.49, 5.81]
Kuo 2012	13	131	12	135	2.0%	1.13 [0.50, 2.57]
Kuo SH 2018	100	275	37	153	2.8%	1.79 [1.15, 2.79]
Lee 2014	75	121	25	177	2.5%	9.91 [5.66, 17.36]
Lodise 2003	16	48	23	119	2.1%	2.09 [0.98, 4.43]
Lye 2012	77	294	73	381	2.9%	1.50 [1.04, 2.16]
Ma 2017	14	39	20	129	2.0%	3.05 [1.36, 6.86]
Metan 2009	53	76	10	24	1.8%	3.23 [1.25, 8.32]
Molina 2013	1	13	0	18	0.3%	4.44 [0.17, 118.00]
Park 2013	27	76	17	73	2.2%	1.82 [0.89, 3.72]
Rodriguez-Bano 2010	13	43	16	53	1.9%	1.00 [0.42, 2.41]
Rodriguez-Pardo 2014	22	134	15	157	2.2%	1.86 [0.92, 3.75]
Rong 2012	21	46	12	72	1.9%	4.20 [1.80, 9.82]
Saliba 2017	27	215	49	331	2.7%	0.83 [0.50, 1.37]
Sancho 2012	106	155	114	216	2.8%	1.94 [1.26, 2.98]
Shargian-Alon 2019	22	24	11	22	0.9%	11.00 [2.07, 58.52]
Trecarichi 2016	79	152	22	126	2.5%	5.12 [2.92, 8.95]
Wu 2015	70	325	11	157	2.3%	3.64 [1.87, 7.10]
Zhang 2017	13	13	41	213	0.4%	112.23 [6.54, 1926.54]
**Subtotal (95% CI)**		**2901**		**3607**	**54.9%**	**2.33 [1.76, 3.07]**
Total events	1073		823			
Heterogeneity: Tau² = 0.37; Chi² = 111.70, df = 27 (P < 0.00001); I² = 76%						
Test for overall effect: Z = 5.92 (P < 0.00001)						
**1.2.3 Healthcare-associated**						
Lye 2012	77	294	73	381	2.9%	1.50 [1.04, 2.16]
Son 2020	77	214	116	930	3.0%	3.94 [2.81, 5.54]
Trecarichi 2016	79	152	22	126	2.5%	5.12 [2.92, 8.95]
Yoon 2016	63	191	51	154	2.8%	0.99 [0.63, 1.56]
**Subtotal (95% CI)**		**851**		**1591**	**11.2%**	**2.32 [1.13, 4.78]**
Total events	296		262			
Heterogeneity: Tau² = 0.49; Chi² = 36.17, df = 3 (P < 0.00001); I² = 92%						
Test for overall effect: Z = 2.29 (P = 0.02)						
**Total (95% CI)**		**6149**		**15567**	**100.0%**	**2.36 [1.96, 2.84]**
Total events	1942		2408			
Heterogeneity: Tau² = 0.27; Chi² = 206.20, df = 48 (P < 0.00001); I² = 77%						
Test for overall effect: Z = 9.13 (P < 0.00001)						
Test for subgroup differences: Chi² = 0.13, df = 2 (P = 0.94), I² = 0%						

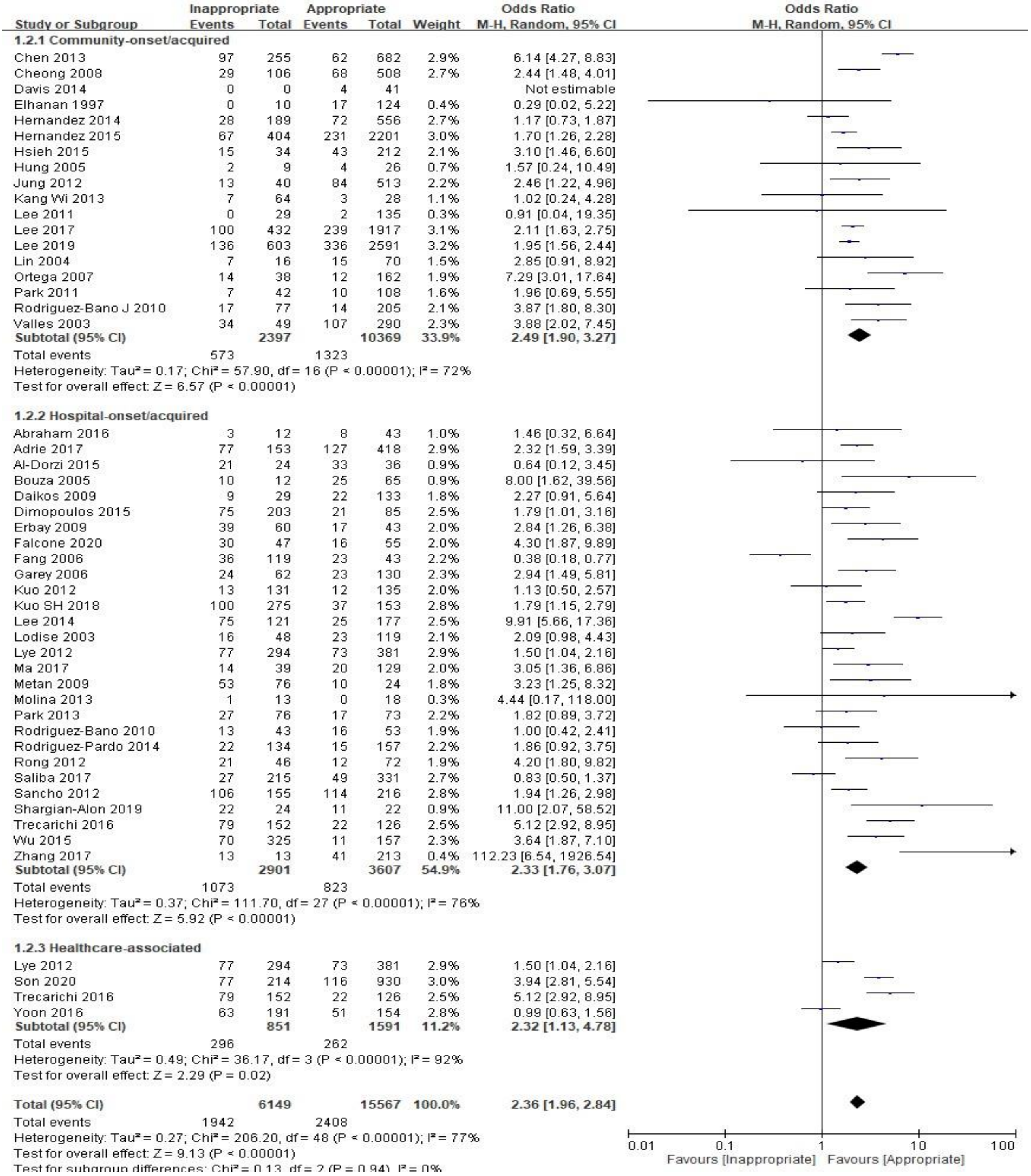

(2B) Specific comorbidity or condition

Study or Subgroup	Inappropriate Events	Inappropriate Total	Appropriate Events	Appropriate Total	Weight	Odds Ratio M-H, Random, 95% CI
**1.3.1 Comorbid haemato-oncology**						
Anatoliotaki 2004	18	46	14	111	3.5%	4.45 [1.97, 10.06]
Bodey 1986	50	62	129	559	3.9%	13.89 [7.18, 26.87]
Bodro 2014	66	268	187	880	4.6%	1.21 [0.88, 1.67]
Carratala 1998	3	5	22	35	1.6%	0.89 [0.13, 6.02]
Gudiol 2011	20	59	62	290	4.0%	1.89 [1.03, 3.46]
Gudiol 2013	7	33	19	59	3.1%	0.57 [0.21, 1.54]
Gudiol 2016	6	9	19	46	2.1%	2.84 [0.63, 12.80]
Kleinhendler 2018	9	14	9	65	2.5%	11.20 [3.05, 41.10]
Ma 2017	14	39	20	129	3.5%	3.05 [1.36, 6.86]
Marin 2014	123	414	40	114	4.4%	0.78 [0.50, 1.21]
Martinez-Nadal 2020	99	394	240	1221	4.7%	1.37 [1.05, 1.79]
Royo-Cebrecos 2017	9	38	36	135	3.5%	0.85 [0.37, 1.98]
Shargian-Alon 2019	22	24	11	22	1.9%	11.00 [2.07, 58.52]
Trecarichi 2009	7	11	5	51	2.1%	16.10 [3.46, 74.84]
Trecarichi 2016	79	152	22	126	4.1%	5.12 [2.92, 8.95]
Wang 2005	50	68	43	303	4.0%	16.80 [8.96, 31.47]
Wang X 2017	10	20	3	20	2.1%	5.67 [1.25, 25.61]
**Subtotal (95% CI)**		**1656**		**4166**	**55.4%**	**3.10 [1.85, 5.19]**
Total events	592		881			
Heterogeneity: Tau² = 0.92; Chi² = 158.26, df = 16 (P < 0.00001); I² = 90%						
Test for overall effect: Z = 4.31 (P < 0.0001)						
**1.3.2 Comorbid liver cirrhosis**						
Bartoletti 2014	48	122	31	190	4.2%	3.33 [1.96, 5.65]
Bartoletti 2018	23	62	24	100	3.8%	1.87 [0.94, 3.72]
Hsieh 2015	15	34	43	212	3.7%	3.10 [1.46, 6.60]
Park H 2015	21	48	10	54	3.3%	3.42 [1.40, 8.35]
Xie 2017	81	240	111	612	4.6%	2.30 [1.64, 3.22]
**Subtotal (95% CI)**		**506**		**1168**	**19.5%**	**2.56 [2.02, 3.26]**
Total events	188		219			
Heterogeneity: Tau² = 0.00; Chi² = 2.79, df = 4 (P = 0.59); I² = 0%						
Test for overall effect: Z = 7.71 (P < 0.00001)						
**1.3.3 Older patients**						
Bartoletti 2018	21	45	20	70	3.6%	2.19 [1.00, 4.78]
Hernandez 2015	67	404	231	2201	4.6%	1.70 [1.26, 2.28]
Meyers 1989	8	16	32	84	2.9%	1.63 [0.55, 4.76]
Rebelo 2011	24	89	6	46	3.1%	2.46 [0.93, 6.54]
**Subtotal (95% CI)**		**554**		**2401**	**14.3%**	**1.78 [1.38, 2.31]**
Total events	120		289			
Heterogeneity: Tau² = 0.00; Chi² = 0.82, df = 3 (P = 0.84); I² = 0%						
Test for overall effect: Z = 4.40 (P < 0.0001)						
**1.3.4 Neutropenic patients**						
Carratala 1998	3	5	22	35	1.6%	0.89 [0.13, 6.02]
Gudiol 2016	6	9	19	46	2.1%	2.84 [0.63, 12.80]
Kleinhendler 2018	9	14	9	65	2.5%	11.20 [3.05, 41.10]
Martinez-Nadal 2020	99	394	240	1221	4.7%	1.37 [1.05, 1.79]
**Subtotal (95% CI)**		**422**		**1367**	**10.8%**	**2.48 [0.85, 7.30]**
Total events	117		290			
Heterogeneity: Tau² = 0.80; Chi² = 10.59, df = 3 (P = 0.01); I² = 72%						
Test for overall effect: Z = 1.65 (P = 0.10)						
**Total (95% CI)**		**3138**		**9102**	**100.0%**	**2.69 [2.01, 3.61]**
Total events	1017		1679			
Heterogeneity: Tau² = 0.46; Chi² = 181.86, df = 29 (P < 0.00001); I² = 84%						
Test for overall effect: Z = 6.66 (P < 0.00001)						
Test for subgroup differences: Chi² = 5.76, df = 3 (P = 0.12), I² = 47.9%						

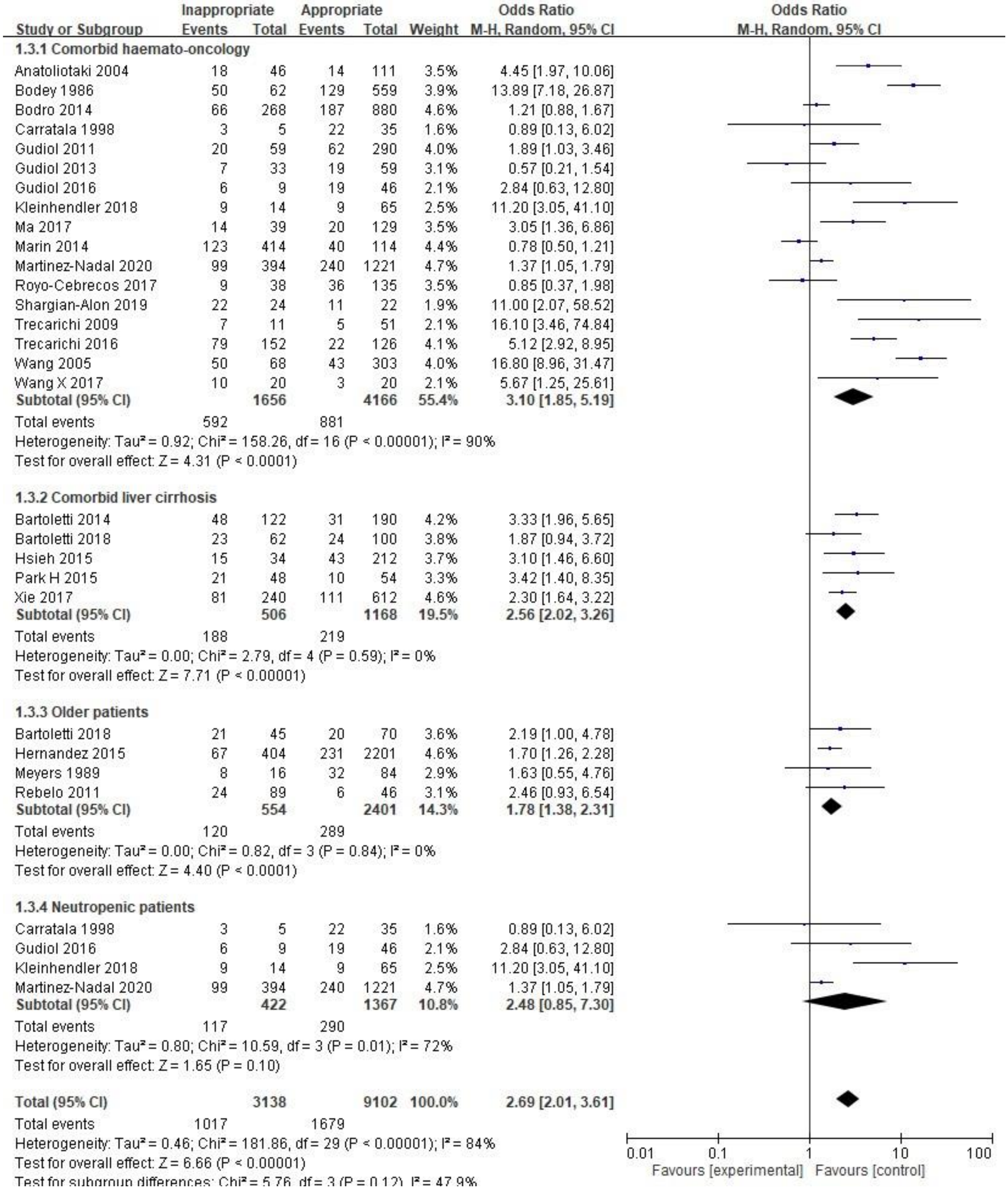

(2C) Bacteraemia source

Study or Subgroup	Inappropriate Events	Inappropriate Total	Appropriate Events	Appropriate Total	Weight	Odds Ratio M-H, Random, 95% CI
**1.4.1 Vascular catheter**						
Garnacho-Montero 2013	44	122	23	66	6.0%	1.05 [0.56, 1.97]
Kuo SH 2018	100	275	37	153	7.6%	1.79 [1.15, 2.79]
Lin 2010	17	23	6	17	2.3%	5.19 [1.33, 20.28]
Rodriguez-Pardo 2014	22	134	15	157	5.4%	1.86 [0.92, 3.75]
Saliba 2017	27	215	49	331	7.0%	0.83 [0.50, 1.37]
**Subtotal (95% CI)**		**769**		**724**	**28.2%**	**1.46 [0.92, 2.32]**
Total events	210		130			
Heterogeneity: Tau² = 0.16; Chi² = 10.33, df = 4 (P = 0.04); I² = 61%						
Test for overall effect: Z = 1.62 (P = 0.11)						
**1.4.2 Pneumonia**						
Calle 2016	14	38	32	60	4.5%	0.51 [0.22, 1.17]
Carratala 1998	3	5	22	35	1.3%	0.89 [0.13, 6.02]
Cheng 2016	34	60	11	51	4.4%	4.76 [2.05, 11.02]
Davis 2014	0	0	4	41		Not estimable
Gudiol 2016	6	9	19	46	1.9%	2.84 [0.63, 12.80]
Guillamet 2016	100	187	282	844	8.8%	2.29 [1.66, 3.16]
Kang 2011	10	64	32	301	4.9%	1.56 [0.72, 3.35]
Park 2012	24	35	27	65	4.3%	3.07 [1.29, 7.31]
Park 2018	80	104	23	42	5.0%	2.75 [1.29, 5.89]
**Subtotal (95% CI)**		**502**		**1485**	**35.0%**	**2.02 [1.28, 3.20]**
Total events	271		452			
Heterogeneity: Tau² = 0.23; Chi² = 17.75, df = 7 (P = 0.01); I² = 61%						
Test for overall effect: Z = 3.02 (P = 0.002)						
**1.4.3 Biliary tract**						
Kang 2013	27	257	22	299	6.3%	1.48 [0.82, 2.66]
Ortega 2012	20	131	106	1242	6.9%	1.93 [1.15, 3.24]
Royo-Cebrecos 2017	9	38	36	135	4.4%	0.85 [0.37, 1.98]
Tagashira 2017	17	133	21	440	5.6%	2.92 [1.49, 5.72]
**Subtotal (95% CI)**		**559**		**2116**	**23.2%**	**1.71 [1.11, 2.64]**
Total events	73		185			
Heterogeneity: Tau² = 0.09; Chi² = 5.54, df = 3 (P = 0.14); I² = 46%						
Test for overall effect: Z = 2.42 (P = 0.02)						
**1.4.4 Urinary tract**						
Elhanan 1997	0	10	17	124	0.6%	0.29 [0.02, 5.22]
Horcajada 2012	15	127	53	540	6.1%	1.23 [0.67, 2.26]
Lee 2011	0	29	2	135	0.5%	0.91 [0.04, 19.35]
Ortega 2013	16	155	46	643	6.2%	1.49 [0.82, 2.72]
**Subtotal (95% CI)**		**321**		**1442**	**13.5%**	**1.31 [0.86, 1.98]**
Total events	31		118			
Heterogeneity: Tau² = 0.00; Chi² = 1.34, df = 3 (P = 0.72); I² = 0%						
Test for overall effect: Z = 1.25 (P = 0.21)						
**Total (95% CI)**		**2151**		**5767**	**100.0%**	**1.66 [1.32, 2.10]**
Total events	585		885			
Heterogeneity: Tau² = 0.13; Chi² = 42.58, df = 20 (P = 0.002); I² = 53%						
Test for overall effect: Z = 4.28 (P < 0.0001)						
Test for subgroup differences: Chi² = 2.17, df = 3 (P = 0.54), I² = 0%						

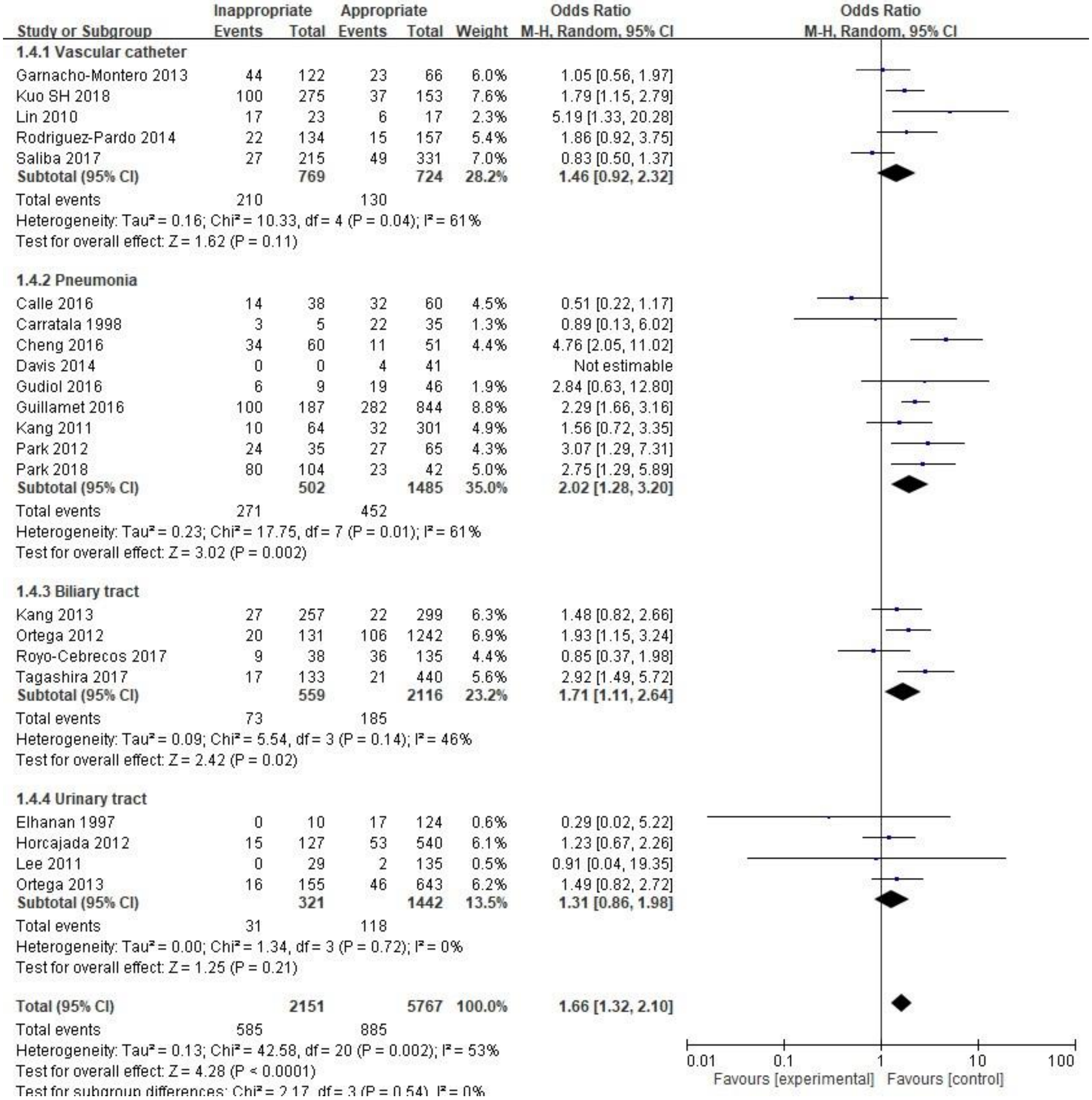

(2D) Bacteraemia severity

Study or Subgroup	Inappropriate Events	Inappropriate Total	Appropriate Events	Appropriate Total	Weight	Odds Ratio M-H, Random, 95% CI
**1.5.1 ICU patients**						
Abraham 2016	3	12	8	43	1.5%	1.46 [0.32, 6.64]
Adrie 2017	77	153	127	418	5.5%	2.32 [1.59, 3.39]
Al-Dorzi 2015	21	24	33	36	1.3%	0.64 [0.12, 3.45]
Corona 2010	415	1013	244	689	6.2%	1.27 [1.04, 1.55]
Dimopoulos 2015	75	203	21	85	4.5%	1.79 [1.01, 3.16]
Falcone 2020	30	47	16	55	3.3%	4.30 [1.87, 9.89]
Haruki 2018	7	14	14	87	2.1%	5.21 [1.58, 17.20]
Ibrahim 2000	91	147	98	345	5.3%	4.10 [2.73, 6.15]
Kuo SH 2018	100	275	37	153	5.1%	1.79 [1.15, 2.79]
Lee 2014	75	121	25	177	4.5%	9.91 [5.66, 17.36]
Sancho 2012	106	155	114	216	5.2%	1.94 [1.26, 2.98]
Savage 2016	135	266	341	924	6.0%	1.76 [1.34, 2.32]
Shorr 2014	54	93	11	38	3.4%	3.40 [1.51, 7.66]
Valles 2003	34	49	107	290	4.1%	3.88 [2.02, 7.45]
Yamaga & Shime 2017	14	16	16	46	1.4%	13.13 [2.65, 65.07]
Zaragoza 2003	22	39	64	127	3.8%	1.27 [0.62, 2.62]
**Subtotal (95% CI)**		**2627**		**3729**	**63.2%**	**2.58 [1.88, 3.54]**
Total events	1259		1276			
Heterogeneity: Tau² = 0.28; Chi² = 85.26, df = 15 (P < 0.00001); I² = 82%						
Test for overall effect: Z = 5.86 (P < 0.00001)						
**1.5.2 severe sepsis or septic shock**						
Byl 1999	10	11	24	36	0.8%	5.00 [0.57, 43.76]
Johnson 2011	125	235	185	519	5.8%	2.05 [1.50, 2.81]
Labelle A 2012	321	470	224	436	6.0%	2.04 [1.56, 2.67]
Schechner 2011	8	8	8	14	0.5%	13.00 [0.63, 268.93]
Ziberberg 2014	106	245	205	819	5.9%	2.28 [1.70, 3.08]
**Subtotal (95% CI)**		**969**		**1824**	**18.9%**	**2.14 [1.81, 2.53]**
Total events	570		646			
Heterogeneity: Tau² = 0.00; Chi² = 2.33, df = 4 (P = 0.68); I² = 0%						
Test for overall effect: Z = 8.89 (P < 0.00001)						
**1.5.3 Pitt bacteraemia score ≥ 4**						
Lee 2013	32	63	21	67	3.8%	2.26 [1.11, 4.62]
Lee 2019	70	120	232	526	5.4%	1.77 [1.19, 2.65]
**Subtotal (95% CI)**		**183**		**593**	**9.1%**	**1.88 [1.33, 2.67]**
Total events	102		253			
Heterogeneity: Tau² = 0.00; Chi² = 0.34, df = 1 (P = 0.56); I² = 0%						
Test for overall effect: Z = 3.54 (P = 0.0004)						
**1.5.4 Non-ICU patients**						
Marschall 2008	11	79	24	171	3.5%	0.99 [0.46, 2.14]
Molina 2013	1	13	0	18	0.4%	4.44 [0.17, 118.00]
Saliba 2017	27	215	49	331	4.8%	0.83 [0.50, 1.37]
**Subtotal (95% CI)**		**307**		**520**	**8.8%**	**0.90 [0.59, 1.36]**
Total events	39		73			
Heterogeneity: Tau² = 0.00; Chi² = 1.08, df = 2 (P = 0.58); I² = 0%						
Test for overall effect: Z = 0.51 (P = 0.61)						
**Total (95% CI)**		**4086**		**6666**	**100.0%**	**2.24 [1.81, 2.77]**
Total events	1970		2248			
Heterogeneity: Tau² = 0.18; Chi² = 103.61, df = 25 (P < 0.00001); I² = 76%						
Test for overall effect: Z = 7.41 (P < 0.00001)						
Test for subgroup differences: Chi² = 17.34, df = 3 (P = 0.0006), I² = 82.7%						

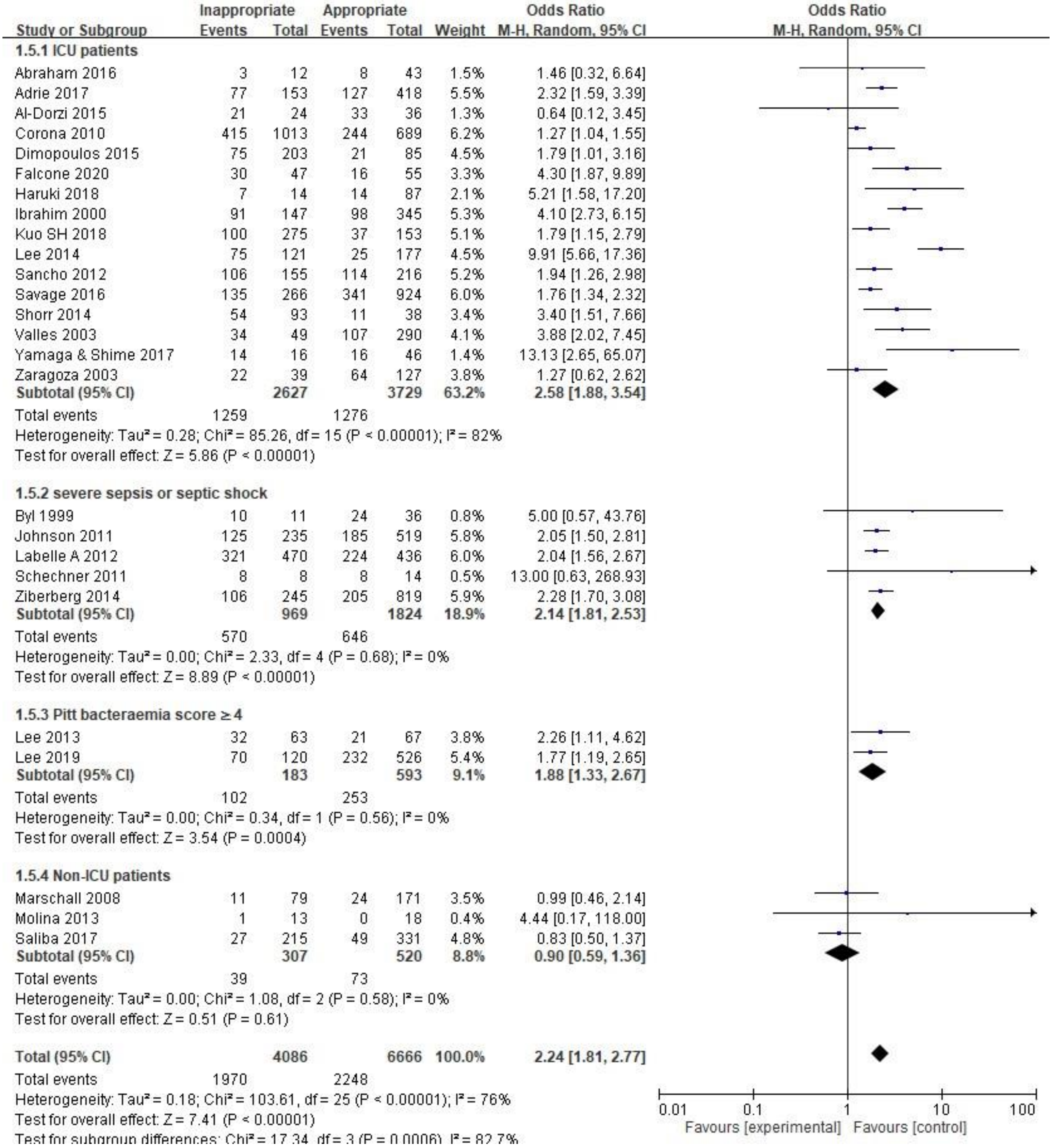

(2E) Staphylococcus aureus

Study or Subgroup	Inappropriate Events	Inappropriate Total	Appropriate Events	Appropriate Total	Weight	Odds Ratio M-H, Random, 95% CI
**1.6.1 Hospital-onset/acquired**						
Fang 2006	36	119	23	43	4.0%	0.38 [0.18, 0.77]
Lodise 2003	16	48	23	119	3.9%	2.09 [0.98, 4.43]
Rong 2012	21	46	12	72	3.5%	4.20 [1.80, 9.82]
Yoon 2016	63	191	51	154	5.3%	0.99 [0.63, 1.56]
**Subtotal (95% CI)**		**404**		**388**	**16.7%**	**1.31 [0.54, 3.19]**
Total events	136		109			
Heterogeneity: Tau² = 0.70; Chi² = 21.07, df = 3 (P = 0.0001); I² = 86%						
Test for overall effect: Z = 0.59 (P = 0.55)						
**1.6.2 Community-acquired**						
Lin 2004	7	16	15	70	2.5%	2.85 [0.91, 8.92]
**Subtotal (95% CI)**		**16**		**70**	**2.5%**	**2.85 [0.91, 8.92]**
Total events	7		15			
Heterogeneity: Not applicable						
Test for overall effect: Z = 1.80 (P = 0.07)						
**1.6.3 Mixed**						
Ammerlaan 2009	20	94	60	240	4.7%	0.81 [0.46, 1.44]
Asgeirsson 2011	9	63	36	262	3.7%	1.05 [0.48, 2.30]
Bassetti 2012	21	56	14	109	3.8%	4.07 [1.87, 8.88]
Bassetti 2018	21	45	20	70	3.8%	2.19 [1.00, 4.78]
Calle 2016	14	38	32	60	3.5%	0.51 [0.22, 1.17]
Gasch 2013	31	201	18	378	4.5%	3.65 [1.98, 6.70]
Guilarde 2006	22	44	17	67	3.6%	2.94 [1.31, 6.59]
Khatib 2006	21	60	60	282	4.6%	1.99 [1.09, 3.64]
Kim 2006	45	117	34	121	4.9%	1.60 [0.93, 2.76]
Lee 2015	24	41	31	148	4.0%	5.33 [2.55, 11.13]
Li 2017	27	112	11	63	3.8%	1.50 [0.69, 3.28]
Marra 2006	6	27	7	64	2.4%	2.33 [0.70, 7.72]
Paul 2010	168	342	56	168	5.6%	1.93 [1.31, 2.84]
Schramm 2006	99	390	28	169	5.3%	1.71 [1.08, 2.73]
Schweizer 2010	77	277	109	537	5.9%	1.51 [1.08, 2.12]
Soriano 2000	33	189	77	719	5.4%	1.76 [1.13, 2.75]
Soriano 2008	73	246	43	168	5.4%	1.23 [0.79, 1.91]
Takesue 2011	41	106	3	22	2.2%	3.99 [1.11, 14.35]
Yilmaz 2016	12	72	27	183	3.9%	1.16 [0.55, 2.43]
**Subtotal (95% CI)**		**2520**		**3830**	**80.8%**	**1.78 [1.42, 2.22]**
Total events	764		683			
Heterogeneity: Tau² = 0.13; Chi² = 44.59, df = 18 (P = 0.0005); I² = 60%						
Test for overall effect: Z = 5.07 (P < 0.00001)						
**Total (95% CI)**		**2940**		**4288**	**100.0%**	**1.71 [1.36, 2.15]**
Total events	907		807			
Heterogeneity: Tau² = 0.20; Chi² = 71.96, df = 23 (P < 0.00001); I² = 68%						
Test for overall effect: Z = 4.60 (P < 0.00001)						
Test for subgroup differences: Chi² = 1.11, df = 2 (P = 0.57), I² = 0%						

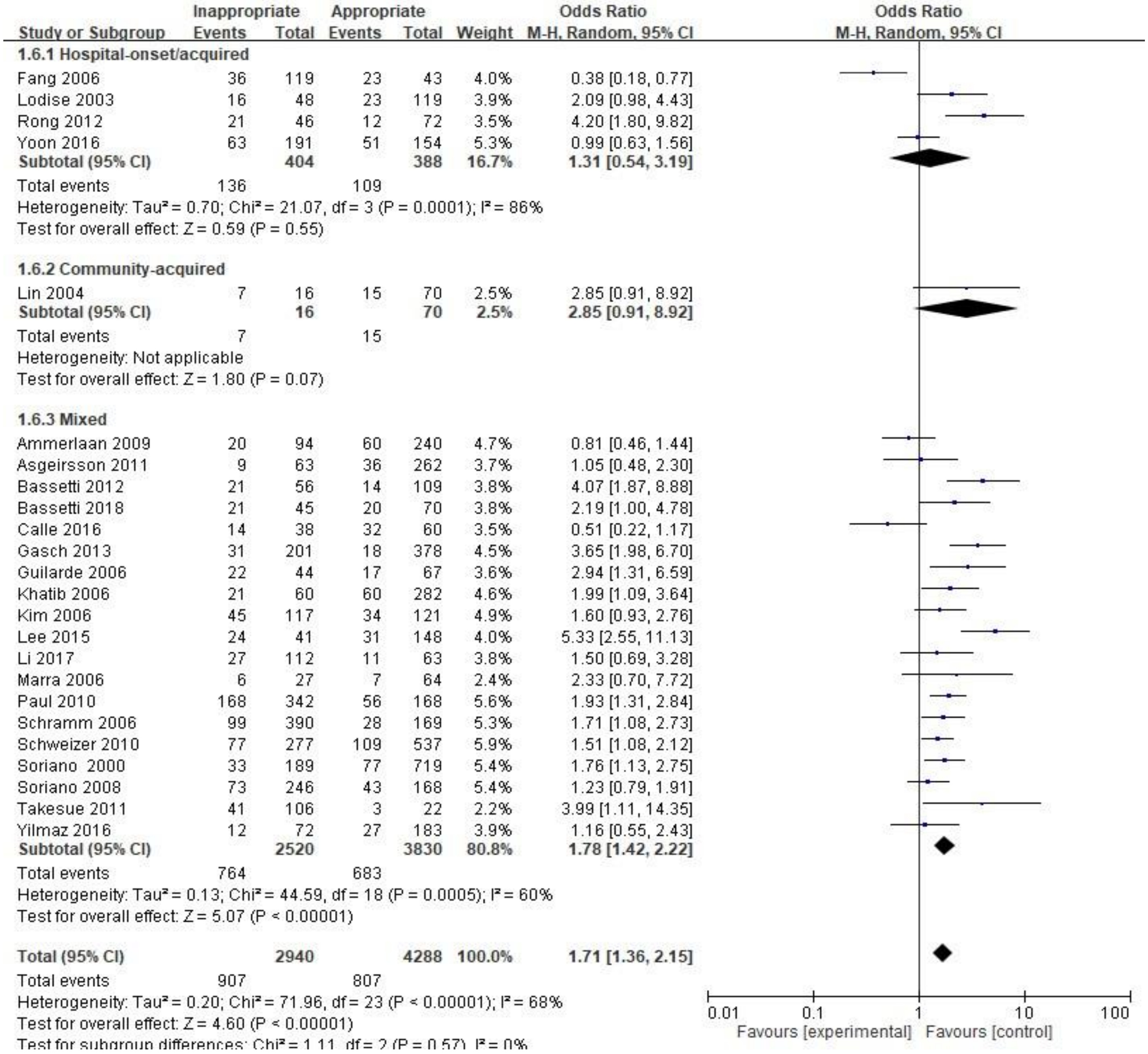

(2F) Enterobacteriaceae, by acquisition places places

Study or Subgroup	Inappropriate Events	Inappropriate Total	Appropriate Events	Appropriate Total	Weight	Odds Ratio M-H, Random, 95% CI
**1.7.1 Hospital-onset/acquired**						
Daikos 2009	9	29	22	133	2.2%	2.27 [0.91, 5.64]
Falcone 2020	30	47	16	55	2.3%	4.30 [1.87, 9.89]
Ma 2017	14	39	20	129	2.4%	3.05 [1.36, 6.86]
Rodriguez-Bano 2010	13	43	16	53	2.3%	1.00 [0.42, 2.41]
Trecarichi 2016	79	152	22	126	2.7%	5.12 [2.92, 8.95]
**Subtotal (95% CI)**		**310**		**496**	**11.8%**	**2.87 [1.62, 5.05]**
Total events	145		96			
Heterogeneity: $Tau^2 = 0.26$; $Chi^2 = 10.50$, df = 4 (P = 0.03); $I^2 = 62\%$						
Test for overall effect: Z = 3.64 (P = 0.0003)						
**1.7.2 Community-onset/acquired**						
Jung 2012	13	40	84	513	2.5%	2.46 [1.22, 4.96]
Kang Wi 2013	7	64	3	28	1.6%	1.02 [0.24, 4.28]
Park 2011	7	42	10	108	2.1%	1.96 [0.69, 5.55]
Rodriguez-Bano J 2010	17	77	14	205	2.4%	3.87 [1.80, 8.30]
**Subtotal (95% CI)**		**223**		**854**	**8.5%**	**2.53 [1.63, 3.92]**
Total events	44		111			
Heterogeneity: $Tau^2 = 0.00$; $Chi^2 = 2.96$, df = 3 (P = 0.40); $I^2 = 0\%$						
Test for overall effect: Z = 4.12 (P < 0.0001)						
**1.7.3 Mixed**						
Abhilash 2010	13	47	17	74	2.3%	1.28 [0.55, 2.96]
Alvarez-Marin 2020	21	117	34	168	2.6%	0.86 [0.47, 1.58]
Bodey 1986	50	62	129	559	2.5%	13.89 [7.18, 26.87]
Chang 2009	6	33	3	55	1.6%	3.85 [0.89, 16.62]
Cheng 2016	34	60	11	51	2.3%	4.76 [2.05, 11.02]
Falcone 2014	15	52	20	42	2.3%	0.45 [0.19, 1.05]
Feldman 1990	2	6	24	41	1.2%	0.35 [0.06, 2.16]
Frakking 2013	30	147	16	85	2.5%	1.11 [0.56, 2.17]
Girometti 2014	40	124	18	93	2.6%	1.98 [1.05, 3.75]
Haruki 2018	7	14	14	87	1.9%	5.21 [1.58, 17.20]
Li 2017	7	25	19	79	2.1%	1.23 [0.45, 3.39]
Lim 2019	10	57	12	53	2.2%	0.73 [0.28, 1.86]
Lin 2006	12	41	10	67	2.2%	2.36 [0.91, 6.10]
Man 2017	100	364	76	489	2.9%	2.06 [1.47, 2.88]
Marcos 2008	10	82	27	232	2.4%	1.05 [0.49, 2.29]
Metan 2005	5	41	9	12	1.4%	0.05 [0.01, 0.23]
Ortega 2009	100	349	340	4409	3.0%	4.81 [3.72, 6.22]
Ortega 2011	14	86	82	824	2.6%	1.76 [0.95, 3.26]
Peralta 2007	12	106	24	557	2.5%	2.84 [1.37, 5.86]
Peralta 2012	47	189	34	198	2.7%	1.60 [0.97, 2.62]
Picot-Gueraud 2015	21	91	30	137	2.6%	1.07 [0.57, 2.02]
Rosa 2011	6	17	11	74	1.9%	3.12 [0.96, 10.20]
Satlin 2017	31	66	28	55	2.5%	0.85 [0.42, 1.75]
Seo 2020	16	79	16	54	2.4%	0.60 [0.27, 1.34]
Shih 2005	20	41	9	30	2.1%	2.22 [0.82, 5.99]
Trecarichi 2009	7	11	5	51	1.5%	16.10 [3.46, 74.84]
Tumbarello 2008	29	56	9	73	2.3%	7.64 [3.19, 18.28]
Tumbarello 2010	11	27	6	107	1.9%	11.57 [3.75, 35.68]
Tumbarello 2012	39	75	13	50	2.4%	3.08 [1.42, 6.71]
Tumbarello 2013	16	21	14	78	1.9%	14.63 [4.59, 46.61]
Tuon 2011	20	49	28	55	2.4%	0.67 [0.31, 1.45]
Wang 2011	15	63	12	50	2.3%	0.99 [0.41, 2.36]
Wu 2012	9	38	4	24	1.7%	1.55 [0.42, 5.74]
Ye 2006	33	68	17	58	2.4%	2.27 [1.09, 4.76]
Yu 2017	16	25	11	24	1.9%	2.10 [0.67, 6.60]
Zarkotou 2011	13	39	5	14	1.8%	0.90 [0.25, 3.24]
**Subtotal (95% CI)**		**2768**		**9109**	**79.6%**	**1.89 [1.38, 2.57]**
Total events	837		1137			
Heterogeneity: $Tau^2 = 0.68$; $Chi^2 = 209.78$, df = 35 (P < 0.00001); $I^2 = 83\%$						
Test for overall effect: Z = 4.02 (P < 0.0001)						
**Total (95% CI)**		**3301**		**10459**	**100.0%**	**2.01 [1.55, 2.61]**
Total events	1026		1344			
Heterogeneity: $Tau^2 = 0.58$; $Chi^2 = 227.13$, df = 44 (P < 0.00001); $I^2 = 81\%$						
Test for overall effect: Z = 5.23 (P < 0.00001)						
Test for subgroup differences: $Chi^2 = 2.15$, df = 2 (P = 0.34), $I^2 = 7.0\%$						

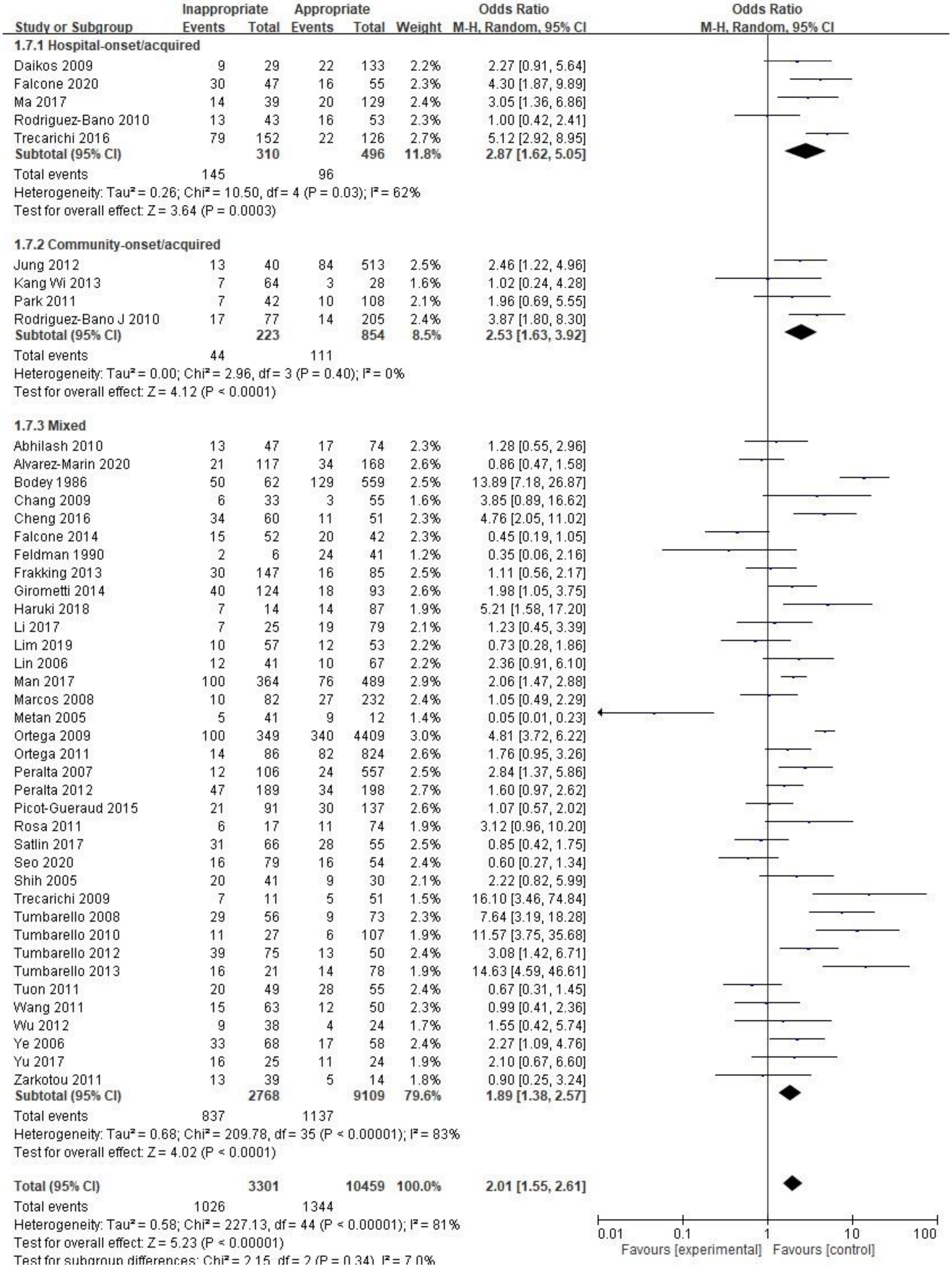

(2G) Enterobacteriaceae, by species

Study or Subgroup	Inappropriate Events	Inappropriate Total	Appropriate Events	Appropriate Total	Weight	Odds Ratio M-H, Random, 95% CI
**1.8.1 E. coli**						
Bodey 1986	80	62	129	559		Not estimable
Haruki 2018	7	14	14	87	1.9%	5.21 [1.58, 17.20]
Kang Wi 2013	7	64	3	28	1.6%	1.02 [0.24, 4.28]
Ma 2017	14	39	20	129	2.4%	3.05 [1.36, 6.86]
Metan 2005	5	41	9	12	1.4%	0.05 [0.01, 0.23]
Ortega 2009	100	349	340	4409	3.1%	4.81 [3.72, 6.22]
Park 2011	7	42	10	108	2.1%	1.96 [0.69, 5.55]
Peralta 2007	12	106	24	557	2.5%	2.84 [1.37, 5.86]
Rodriguez-Bano 2010	17	77	14	205	2.5%	3.87 [1.80, 8.30]
Rodriguez-Bano J 2010	13	43	16	53	2.3%	1.00 [0.42, 2.41]
Trecarichi 2009	7	11	5	51	1.5%	16.10 [3.46, 74.84]
Tumbarello 2008	29	56	9	73	2.3%	7.64 [3.19, 18.28]
Tumbarello 2010	11	27	6	107	2.0%	11.57 [3.75, 35.68]
Wu 2012	9	38	4	24	1.7%	1.55 [0.42, 5.74]
**Subtotal (95% CI)**		**969**		**6402**	**27.3%**	**2.77 [1.62, 4.72]**
Total events	318		603			
Heterogeneity: Tau² = 0.68; Chi² = 59.84, df = 12 (P < 0.00001); I² = 80%						
Test for overall effect: Z = 3.73 (P = 0.0002)						
**1.8.2 K. pneumoniae**						
Daikos 2009	9	29	22	133	2.3%	2.27 [0.91, 5.64]
Falcone 2020	30	47	16	55	2.4%	4.30 [1.87, 9.89]
Feldman 1990	2	6	24	41	1.2%	0.35 [0.06, 2.16]
Girometti 2014	40	124	18	93	2.7%	1.98 [1.05, 3.75]
Jung 2012	13	40	84	513	2.6%	2.46 [1.22, 4.96]
Li 2017	7	25	19	79	2.1%	1.23 [0.45, 3.39]
Man 2017	100	364	76	489	3.1%	2.06 [1.47, 2.88]
Ortega 2011	14	86	82	824	2.7%	1.76 [0.95, 3.26]
Trecarichi 2016	79	152	22	126	2.8%	5.12 [2.92, 8.95]
Tumbarello 2012	39	75	13	50	2.5%	3.08 [1.42, 6.71]
Tuon 2011	20	49	28	55	2.5%	0.67 [0.31, 1.45]
Yu 2017	16	25	11	24	1.9%	2.10 [0.67, 6.60]
Zarkotou 2011	13	39	5	14	1.8%	0.90 [0.25, 3.24]
**Subtotal (95% CI)**		**1061**		**2496**	**30.4%**	**2.02 [1.46, 2.80]**
Total events	382		420			
Heterogeneity: Tau² = 0.19; Chi² = 28.94, df = 12 (P = 0.004); I² = 59%						
Test for overall effect: Z = 4.22 (P < 0.0001)						
**1.8.3 Enterobacter spp.**						
Alvarez-Marin 2020	21	117	34	168	2.7%	0.86 [0.47, 1.58]
Chang 2009	6	33	3	55	1.5%	3.85 [0.89, 16.62]
Lin 2006	12	41	10	67	2.2%	2.36 [0.91, 6.10]
Marcos 2008	10	82	27	232	2.5%	1.05 [0.49, 2.29]
Ye 2006	33	68	17	58	2.5%	2.27 [1.09, 4.76]
**Subtotal (95% CI)**		**341**		**580**	**11.5%**	**1.56 [0.93, 2.62]**
Total events	82		91			
Heterogeneity: Tau² = 0.16; Chi² = 7.64, df = 4 (P = 0.11); I² = 48%						
Test for overall effect: Z = 1.68 (P = 0.09)						
**1.8.4 Other species**						
Picot-Gueraud 2015	21	91	30	137	2.7%	1.07 [0.57, 2.02]
Shih 2005	20	41	9	30	2.2%	2.22 [0.82, 5.99]
Tumbarello 2013	16	21	14	78	1.9%	14.63 [4.59, 46.61]
**Subtotal (95% CI)**		**153**		**245**	**6.7%**	**3.07 [0.72, 13.08]**
Total events	57		53			
Heterogeneity: Tau² = 1.41; Chi² = 15.12, df = 2 (P = 0.0005); I² = 87%						
Test for overall effect: Z = 1.51 (P = 0.13)						
**1.8.5 Mixed species**						
Abhilash 2010	13	47	17	74	2.4%	1.28 [0.55, 2.96]
Falcone 2014	15	52	20	42	2.4%	0.45 [0.19, 1.05]
Frakking 2013	30	147	16	85	2.6%	1.11 [0.56, 2.17]
Lim 2019	10	57	12	53	2.2%	0.73 [0.28, 1.86]
Peralta 2012	47	189	34	198	2.9%	1.60 [0.97, 2.62]
Rosa 2011	6	17	11	74	1.9%	3.12 [0.96, 10.20]
Satlin 2017	31	66	28	55	2.6%	0.85 [0.42, 1.75]
Seo 2020	16	79	16	54	2.4%	0.60 [0.27, 1.34]
Wang 2011	15	63	12	50	2.3%	0.99 [0.41, 2.36]
Ye 2006	34	60	11	51	2.4%	4.76 [2.05, 11.02]
**Subtotal (95% CI)**		**777**		**736**	**24.0%**	**1.17 [0.77, 1.76]**
Total events	217		177			
Heterogeneity: Tau² = 0.27; Chi² = 24.34, df = 9 (P = 0.004); I² = 63%						
Test for overall effect: Z = 0.73 (P = 0.46)						
**Total (95% CI)**		**3301**		**10459**	**100.0%**	**1.91 [1.49, 2.46]**
Total events	1056		1344			
Heterogeneity: Tau² = 0.51; Chi² = 198.41, df = 43 (P < 0.00001); I² = 78%						
Test for overall effect: Z = 5.05 (P < 0.00001)						
Test for subgroup differences: Chi² = 8.07, df = 4 (P = 0.09), I² = 50.4%						

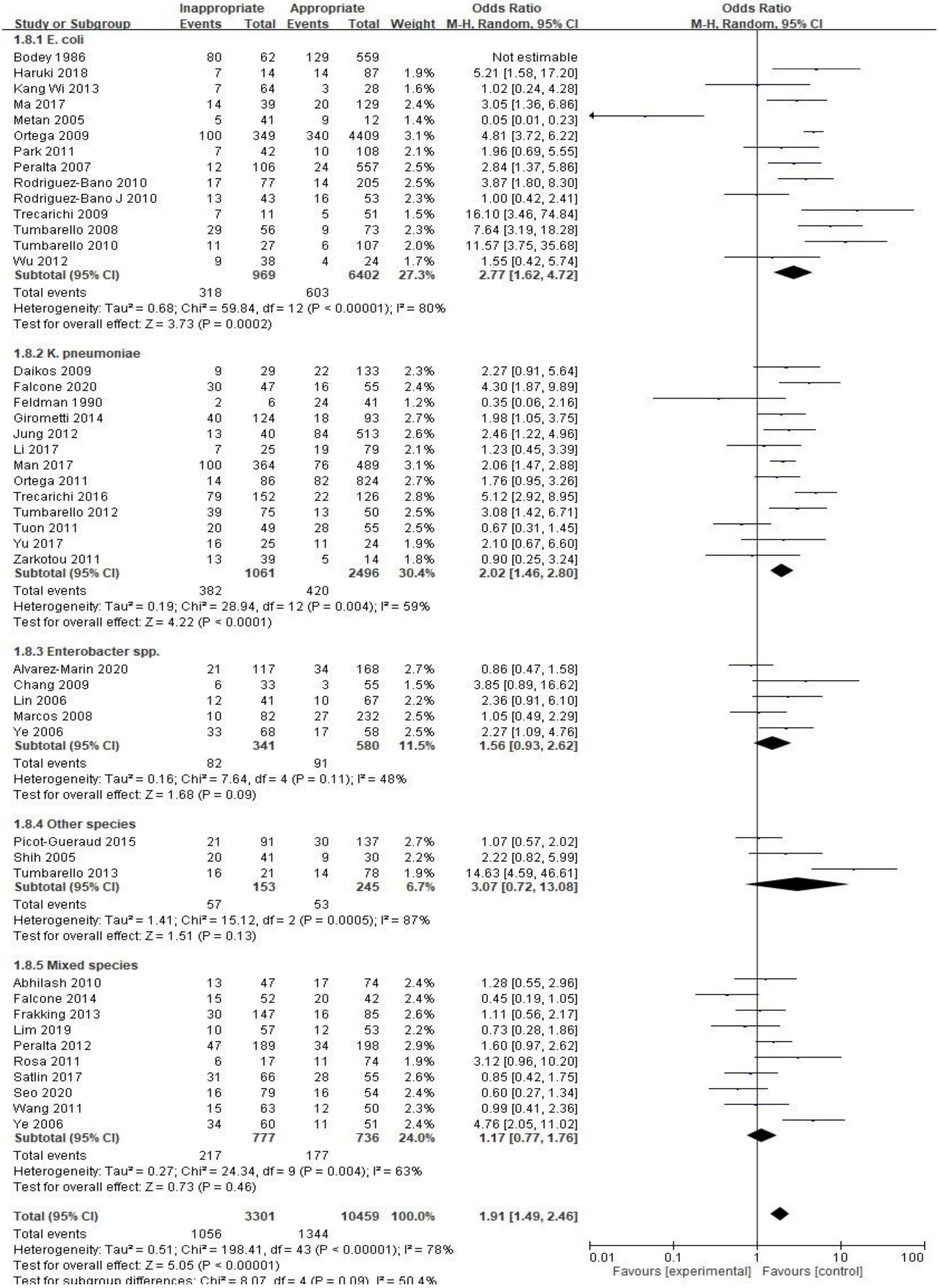

(2H) Glucose non-fermentative rods

Study or Subgroup	Inappropriate Events	Inappropriate Total	Appropriate Events	Appropriate Total	Weight	Odds Ratio M-H, Random, 95% CI
**1.9.1 Pseudomonas spp.**						
Bang 2013	10	43	2	18	1.5%	2.42 [0.47, 12.39]
Chamot 2003	3	17	13	98	1.9%	1.40 [0.35, 5.55]
Horino 2011	14	63	14	71	3.2%	1.16 [0.51, 2.68]
Kang 2003	40	89	13	47	3.4%	2.14 [0.99, 4.58]
Kim 2014	7	38	45	296	3.0%	1.26 [0.52, 3.03]
Lodise 2007	21	48	10	52	3.0%	3.27 [1.33, 7.99]
Martinez-Nadal 2020	99	394	240	1221	4.8%	1.37 [1.05, 1.79]
Micek & Lioyd 2010	23	75	41	230	3.9%	2.04 [1.12, 3.70]
Migiyama 2016	23	49	17	77	3.3%	3.12 [1.43, 6.80]
Morata 2012	65	246	76	463	4.6%	1.83 [1.26, 2.66]
Osih 2007	26	68	35	99	3.8%	1.13 [0.60, 2.15]
Park 2012	24	35	27	65	3.1%	3.07 [1.29, 7.31]
Park 2013	86	261	90	332	4.6%	1.32 [0.93, 1.88]
Schechner 2011	15	32	12	44	2.8%	2.35 [0.90, 6.15]
Tam 2010	6	16	14	93	2.3%	3.39 [1.06, 10.81]
Tumbarello 2011	20	37	16	69	3.1%	3.90 [1.66, 9.16]
Vitkauskiene 2010	18	31	29	49	2.9%	0.95 [0.38, 2.38]
**Subtotal (95% CI)**		**1542**		**3324**	**55.1%**	**1.74 [1.45, 2.10]**
Total events	500		694			
Heterogeneity: Tau² = 0.03; Chi² = 21.24, df = 16 (P = 0.17); I² = 25%						
Test for overall effect: Z = 5.83 (P < 0.00001)						
**1.9.2 Acinetobacter spp.**						
Al-Dorzi 2015	21	24	33	36	1.4%	0.64 [0.12, 3.45]
Chusri 2019	7	34	18	26	2.3%	0.12 [0.04, 0.37]
Davis 2014	0	0	4	41		Not estimable
Erbay 2009	39	60	17	43	3.2%	2.84 [1.26, 6.38]
Falagas 2006	13	22	6	18	2.0%	2.89 [0.79, 10.57]
Kuo 2012	13	131	12	135	3.2%	1.13 [0.50, 2.57]
Lee 2013	32	63	21	67	3.5%	2.26 [1.11, 4.62]
Lee 2014	75	121	25	177	4.0%	9.91 [5.66, 17.36]
Lee NY 2012	63	177	12	91	3.6%	3.64 [1.84, 7.19]
Metan 2009	53	76	10	24	2.8%	3.23 [1.25, 8.32]
Park 2018	80	104	23	42	3.4%	2.75 [1.29, 5.89]
Shargian-Alon 2019	22	24	11	22	1.5%	11.00 [2.07, 58.52]
Shorr 2014	54	93	11	38	3.2%	3.40 [1.51, 7.66]
Song 2011	11	15	4	13	1.5%	6.19 [1.20, 31.97]
Wang X 2017	10	20	3	20	1.7%	5.67 [1.25, 25.61]
Wareham 2008	10	74	7	88	2.6%	1.81 [0.65, 5.01]
**Subtotal (95% CI)**		**1038**		**881**	**40.0%**	**2.55 [1.53, 4.27]**
Total events	503		217			
Heterogeneity: Tau² = 0.74; Chi² = 61.50, df = 14 (P < 0.00001); I² = 77%						
Test for overall effect: Z = 3.57 (P = 0.0004)						
**1.9.3 Burkholderia spp.**						
Ku 2011	6	8	5	19	1.2%	8.40 [1.26, 56.07]
**Subtotal (95% CI)**		**8**		**19**	**1.2%**	**8.40 [1.26, 56.07]**
Total events	6		5			
Heterogeneity: Not applicable						
Test for overall effect: Z = 2.20 (P = 0.03)						
**1.9.4 Mixed**						
Park 2013	44	76	27	73	3.7%	2.34 [1.21, 4.52]
**Subtotal (95% CI)**		**76**		**73**	**3.7%**	**2.34 [1.21, 4.52]**
Total events	44		27			
Heterogeneity: Not applicable						
Test for overall effect: Z = 2.54 (P = 0.01)						
**Total (95% CI)**		**2664**		**4297**	**100.0%**	**2.20 [1.73, 2.79]**
Total events	1053		943			
Heterogeneity: Tau² = 0.29; Chi² = 103.30, df = 33 (P < 0.00001); I² = 68%						
Test for overall effect: Z = 6.48 (P < 0.00001)						
Test for subgroup differences: Chi² = 4.81, df = 3 (P = 0.19), I² = 37.6%						

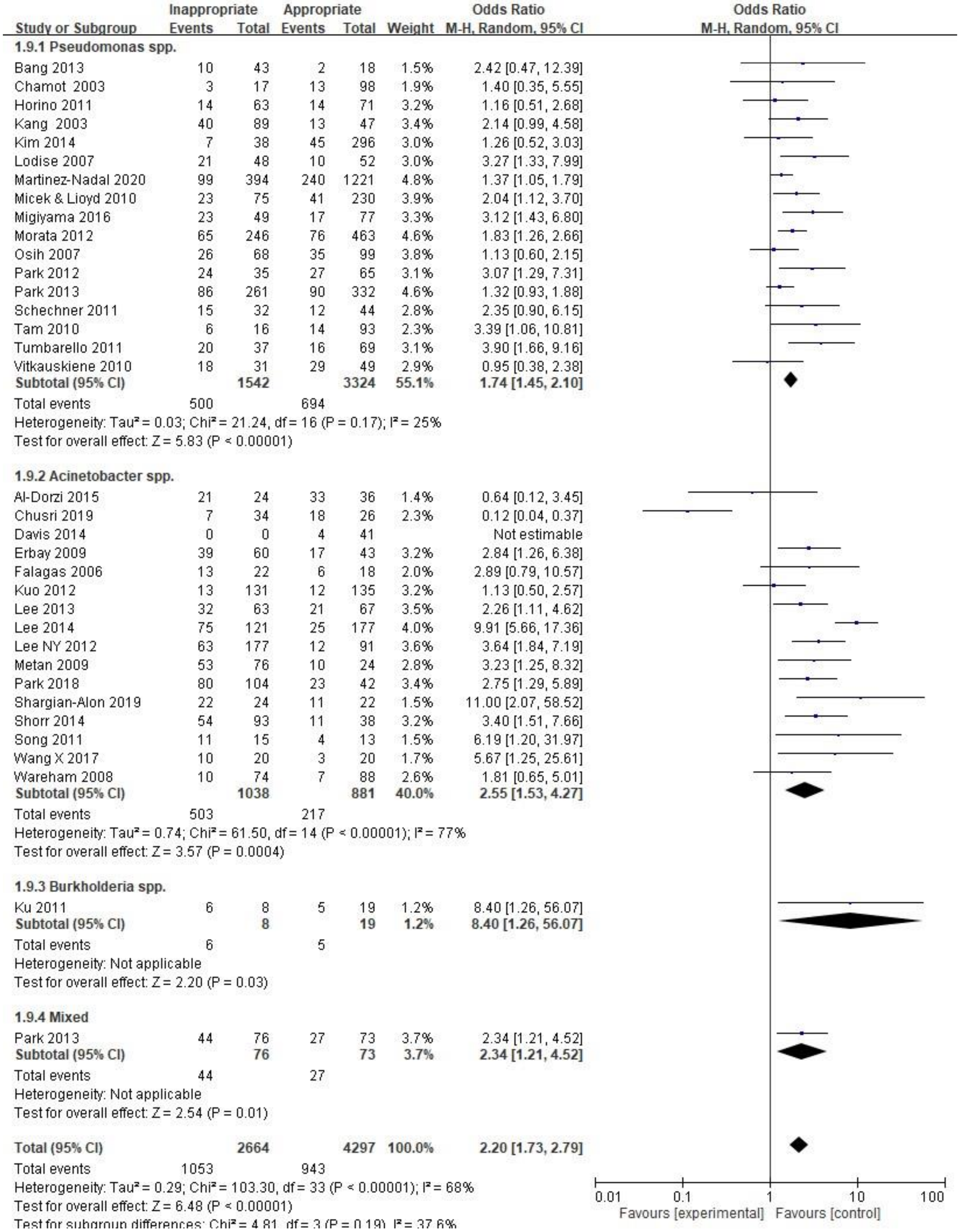

(2I) Antibiotic -resistant microorganism

Study or Subgroup	Inappropriate Events	Inappropriate Total	Appropriate Events	Appropriate Total	Weight	Odds Ratio M-H, Random, 95% CI
**1.10.1 MRSA**						
Fang 2006	36	119	23	43	3.4%	0.38 [0.18, 0.77]
Gasch 2013	31	201	18	378	3.7%	3.65 [1.98, 6.70]
Lee 2015	24	41	31	148	3.4%	5.33 [2.55, 11.13]
Lewis 2011	27	112	11	63	3.3%	1.50 [0.69, 3.28]
Paul 2010	168	342	56	168	4.1%	1.93 [1.31, 2.84]
Schramm 2006	99	390	28	169	4.0%	1.71 [1.08, 2.73]
Soriano 2008	73	246	43	168	4.0%	1.23 [0.79, 1.91]
Takesue 2011	41	106	3	22	2.3%	3.99 [1.11, 14.35]
Yoon 2016	63	191	51	154	4.0%	0.99 [0.63, 1.56]
**Subtotal (95% CI)**		**1748**		**1313**	**32.2%**	**1.70 [1.10, 2.63]**
Total events	562		264			
Heterogeneity: Tau² = 0.34; Chi² = 41.60, df = 8 (P < 0.00001); I² = 81%						
Test for overall effect: Z = 2.37 (P = 0.02)						
**1.10.2 ESBL-producing Enterobactericeae**						
Cheng 2016	34	60	11	51	3.2%	4.76 [2.05, 11.02]
Falcone 2014	15	52	20	42	3.1%	0.45 [0.19, 1.05]
Frakking 2013	30	147	16	85	3.5%	1.11 [0.56, 2.17]
Kang 2005	58	151	37	135	3.9%	1.65 [1.00, 2.72]
Kang Wi 2013	7	64	3	28	2.0%	1.02 [0.24, 4.28]
Metan 2005	5	41	9	12	1.8%	0.05 [0.01, 0.23]
Peralta 2012	47	189	34	198	3.9%	1.60 [0.97, 2.62]
Rodriguez-Bano 2010	13	43	16	53	3.1%	1.00 [0.42, 2.41]
Rosa 2011	6	17	11	74	2.5%	3.12 [0.96, 10.20]
Tumbarello 2008	29	56	9	73	3.1%	7.64 [3.19, 18.28]
Wang 2011	15	63	12	50	3.1%	0.99 [0.41, 2.36]
Wu 2012	9	38	4	24	2.2%	1.55 [0.42, 5.74]
Yu 2017	16	25	11	24	2.5%	2.10 [0.67, 6.60]
**Subtotal (95% CI)**		**946**		**849**	**38.0%**	**1.39 [0.85, 2.28]**
Total events	284		193			
Heterogeneity: Tau² = 0.58; Chi² = 51.14, df = 12 (P < 0.00001); I² = 77%						
Test for overall effect: Z = 1.32 (P = 0.19)						
**1.10.3 MDR Enterobactericaea**						
Picot-Gueraud 2015	21	91	30	137	3.6%	1.07 [0.57, 2.02]
Wang W 2017	19	47	16	91	3.3%	3.18 [1.44, 7.04]
**Subtotal (95% CI)**		**138**		**228**	**6.9%**	**1.80 [0.62, 5.21]**
Total events	40		46			
Heterogeneity: Tau² = 0.46; Chi² = 4.42, df = 1 (P = 0.04); I² = 77%						
Test for overall effect: Z = 1.08 (P = 0.28)						
**1.10.4 Carbapenem-resistant Enterobactericeae**						
Babar 2021	16	38	3	99	2.2%	23.27 [6.23, 86.88]
Falcone 2020	30	47	16	55	3.2%	4.30 [1.87, 9.89]
Park 2018	80	104	23	42	3.3%	2.75 [1.29, 5.89]
Satlin 2017	31	66	28	55	3.4%	0.85 [0.42, 1.75]
Seo 2020	16	79	16	54	3.3%	0.60 [0.27, 1.34]
Song 2011	11	15	4	13	1.7%	6.19 [1.20, 31.97]
Tumbarello 2012	39	75	13	50	3.3%	3.08 [1.42, 6.71]
Zarkotou 2011	13	39	5	14	2.3%	0.90 [0.25, 3.24]
**Subtotal (95% CI)**		**463**		**382**	**22.8%**	**2.45 [1.16, 5.17]**
Total events	236		108			
Heterogeneity: Tau² = 0.89; Chi² = 35.81, df = 7 (P < 0.00001); I² = 80%						
Test for overall effect: Z = 2.35 (P = 0.02)						
**Total (95% CI)**		**3295**		**2772**	**100.0%**	**1.71 [1.30, 2.26]**
Total events	1122		611			
Heterogeneity: Tau² = 0.45; Chi² = 135.25, df = 31 (P < 0.00001); I² = 77%						
Test for overall effect: Z = 3.81 (P = 0.0001)						
Test for subgroup differences: Chi² = 1.55, df = 3 (P = 0.67), I² = 0%						

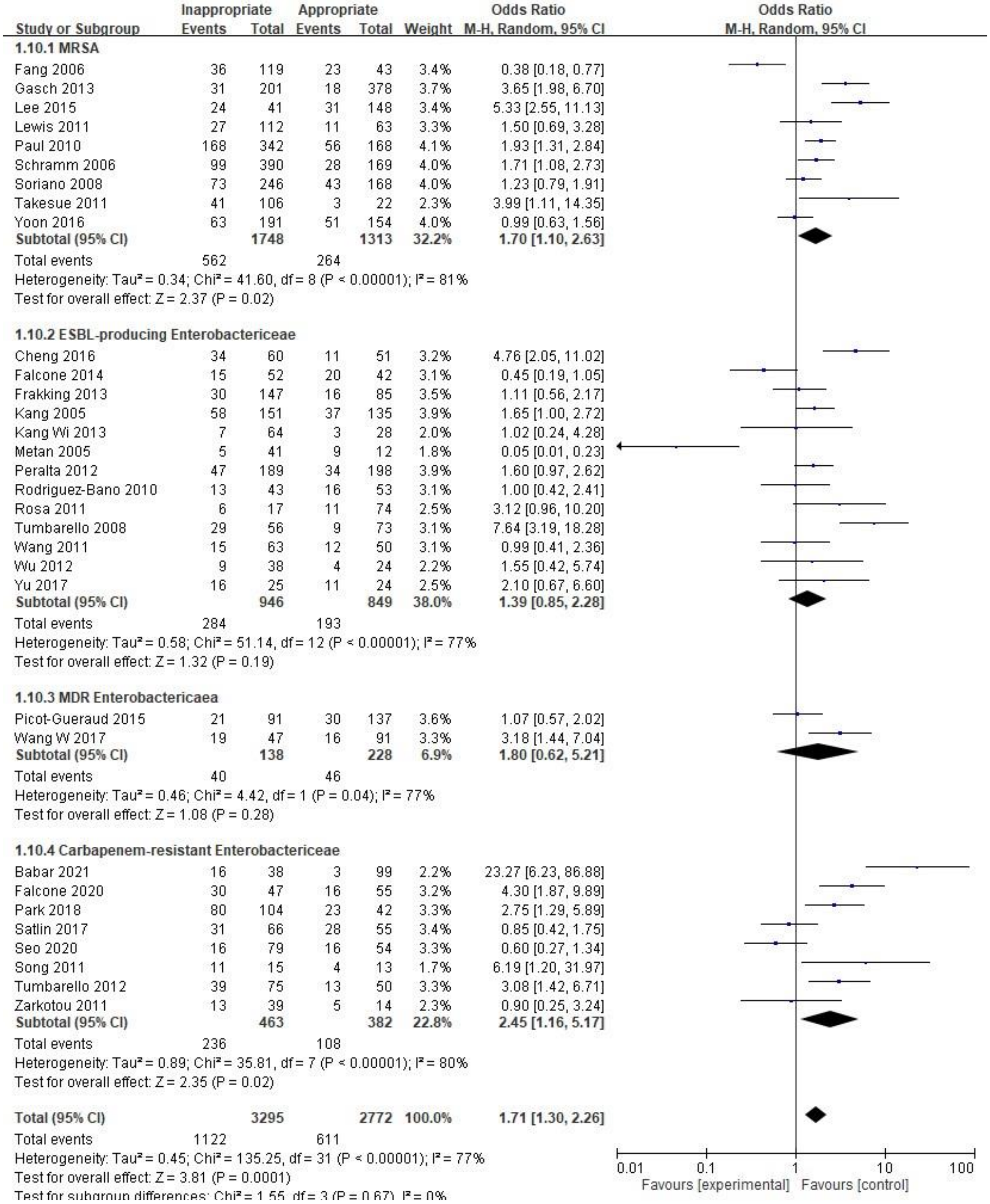

(2J) Varied EAT timeliness

Study or Subgroup	Inappropriate Events	Inappropriate Total	Appropriate Events	Appropriate Total	Weight	Odds Ratio M-H, Random, 95% CI
**1.11.1 0 hour**						
Abhilash 2010	13	47	17	74	0.5%	1.28 [0.55, 2.96]
Babar 2021	16	38	3	99	0.3%	23.27 [6.23, 86.88]
Bodey 1986	50	62	129	559	0.5%	13.89 [7.18, 26.87]
Corona 2010	415	1013	244	689	0.8%	1.27 [1.04, 1.55]
Elhanan 1997	0	10	17	124	0.1%	0.29 [0.02, 5.22]
Enoch 2011	15	40	30	163	0.5%	2.66 [1.25, 5.65]
Feldman 1990	2	6	24	41	0.2%	0.35 [0.06, 2.16]
Feodoroff 2011	1	46	1	30	0.1%	0.64 [0.04, 10.71]
Guillamet 2016	100	187	282	844	0.7%	2.29 [1.66, 3.16]
Hanon 2002	279	768	351	1290	0.8%	1.53 [1.26, 1.85]
Kang Wi 2013	7	64	3	28	0.3%	1.02 [0.24, 4.28]
Khatib 2006	21	60	60	282	0.6%	1.99 [1.09, 3.64]
Klevay 2009	21	78	75	244	0.6%	0.83 [0.47, 1.47]
Lee 2013	32	63	21	67	0.5%	2.26 [1.11, 4.62]
Lee CC 2012	19	124	23	330	0.6%	2.42 [1.26, 4.61]
Lin 2004	7	16	15	70	0.3%	2.85 [0.91, 8.92]
Lin 2006	12	41	10	67	0.4%	2.36 [0.91, 6.10]
Marin 2014	123	414	40	114	0.7%	0.78 [0.50, 1.21]
Martinez-Nadal 2020	99	394	240	1221	0.7%	1.37 [1.05, 1.79]
Metan 2005	5	41	9	12	0.2%	0.05 [0.01, 0.23]
Meyers 1989	8	16	32	84	0.4%	1.63 [0.55, 4.76]
Micek & Lloyd 2010	23	75	41	230	0.6%	2.04 [1.12, 3.70]
Molina 2013	1	13	0	18	0.1%	4.44 [0.17, 118.00]
Park H 2015	21	48	10	54	0.4%	3.42 [1.40, 8.35]
Phillips 1990	67	159	215	1346	0.7%	3.83 [2.71, 5.42]
Royo-Cebrecos 2017	9	38	36	135	0.5%	0.85 [0.37, 1.98]
Trecarichi 2016	79	152	22	126	0.6%	5.12 [2.92, 8.95]
Tumbarello 2010	11	27	6	107	0.3%	11.57 [3.75, 35.68]
Tumbarello 2012	39	75	13	50	0.5%	3.08 [1.42, 6.71]
Wang 2005	50	68	43	303	0.6%	16.80 [8.96, 31.47]
Yilmaz 2016	12	72	27	183	0.5%	1.16 [0.55, 2.43]
Yu 2017	16	25	11	24	0.3%	2.10 [0.67, 6.60]
**Subtotal (95% CI)**		**4280**		**9008**	**14.7%**	**2.15 [1.63, 2.85]**
Total events	1573		2050			
Heterogeneity: Tau² = 0.45; Chi² = 222.15, df = 31 (P < 0.00001); I² = 86%						
Test for overall effect: Z = 5.35 (P < 0.00001)						
**1.11.2 24 hours**						
Abraham 2016	3	12	8	43	0.2%	1.46 [0.32, 6.64]
Adrie 2017	77	153	127	418	0.7%	2.32 [1.59, 3.39]
Alvarez-Marin 2020	21	117	34	168	0.6%	0.86 [0.47, 1.58]
Anatoliotaki 2004	18	46	14	111	0.5%	4.45 [1.97, 10.06]
Bang 2013	10	43	2	18	0.2%	2.42 [0.47, 12.39]
Bartoletti 2014	48	122	31	190	0.6%	3.33 [1.96, 5.65]
Bartoletti 2018	23	62	24	100	0.5%	1.87 [0.94, 3.72]
Boel 2015	79	306	313	1702	0.7%	1.54 [1.16, 2.05]
Bouza 2004	37	120	33	170	0.6%	1.85 [1.08, 3.18]
Bouza 2005	10	12	25	65	0.2%	8.00 [1.62, 39.56]
Byl 1999	37	159	48	269	0.6%	1.40 [0.86, 2.26]
Calle 2016	14	38	32	60	0.5%	0.51 [0.22, 1.17]
Chamot 2003	3	17	13	98	0.3%	1.40 [0.35, 5.55]
Chen 2013	97	255	62	682	0.7%	6.14 [4.27, 8.83]
Cheong 2008	29	106	68	508	0.6%	2.44 [1.48, 4.01]
Davis 2014	0	0	4	41		Not estimable
Falcone 2020	30	47	16	55	0.5%	4.30 [1.87, 9.89]
Frakking 2013	30	147	16	85	0.5%	1.11 [0.56, 2.17]
Garey 2006	24	62	23	130	0.5%	2.94 [1.49, 5.81]
Hernandez 2014	28	189	72	556	0.6%	1.17 [0.73, 1.87]
Hernandez 2015	67	404	231	2201	0.7%	1.70 [1.26, 2.28]
Horcajada 2012	15	127	53	540	0.6%	1.23 [0.67, 2.26]
Horino 2011	14	63	14	71	0.5%	1.16 [0.51, 2.68]
Johnson 2011	125	235	185	519	0.7%	2.05 [1.50, 2.81]
Kang 2003	40	89	13	47	0.5%	2.14 [0.99, 4.58]
Kang 2005	58	151	37	135	0.6%	1.65 [1.00, 2.72]
Kang 2011	10	64	32	301	0.5%	1.56 [0.72, 3.35]
Kang 2013	27	257	22	299	0.6%	1.48 [0.82, 2.66]
Khan 2010	55	65	47	387	0.5%	39.79 [18.99, 83.36]
Kim 2014	7	38	45	296	0.4%	1.26 [0.52, 3.03]
Ku 2011	6	8	5	19	0.2%	8.40 [1.26, 56.07]
Labelle A 2012	321	470	224	436	0.7%	2.04 [1.56, 2.67]
Labelle AJ 2012	60	208	12	37	0.5%	0.84 [0.40, 1.79]
Lee 2019	136	603	336	2591	0.8%	1.95 [1.56, 2.44]
Lim 2019	10	57	12	53	0.4%	0.73 [0.28, 1.86]
Lin 2008	50	540	79	983	0.7%	1.17 [0.81, 1.69]
Lye 2012	77	294	73	381	0.7%	1.50 [1.04, 2.16]
Ma 2017	14	39	20	129	0.5%	3.05 [1.36, 6.86]
Man 2017	100	364	76	489	0.7%	2.06 [1.47, 2.88]
Marra 2006	6	27	7	64	0.3%	2.33 [0.70, 7.72]
Marschall 2008	11	79	24	171	0.5%	0.99 [0.46, 2.14]
Metan 2009	53	76	10	24	0.4%	3.23 [1.25, 8.32]
Micek 2010	270	522	87	238	0.7%	1.86 [1.36, 2.55]
Morata 2012	65	246	76	463	0.7%	1.83 [1.26, 2.66]
Osih 2007	26	68	35	99	0.6%	1.13 [0.60, 2.15]
Park 2011	7	42	10	108	0.4%	1.96 [0.69, 5.55]
Pena 2013	86	261	90	332	0.7%	1.32 [0.93, 1.88]
Peralta 2007	12	106	24	557	0.5%	2.84 [1.37, 5.86]
Peralta 2012	47	189	34	198	0.6%	1.60 [0.97, 2.62]
Picot-Gueraud 2015	21	91	30	137	0.6%	1.07 [0.57, 2.02]
Retamar 2013	17	57	53	284	0.6%	1.85 [0.98, 3.52]
Rodriguez-Bano 2010	13	43	16	53	0.4%	1.00 [0.42, 2.41]
Rodriguez-Bano J 2010	17	77	14	205	0.5%	3.87 [1.80, 8.30]
Satlin 2017	46	102	13	19	0.4%	0.38 [0.13, 1.08]
Savage 2016	135	266	341	924	0.7%	1.76 [1.34, 2.32]
Schechner 2011	15	32	12	44	0.4%	2.35 [0.90, 6.15]
Schramm 2006	99	390	28	169	0.6%	1.71 [1.08, 2.73]
Schweizer 2010	77	277	109	537	0.7%	1.51 [1.08, 2.12]
Shargian-Alon 2019	26	35	7	11	0.3%	1.65 [0.39, 6.99]
Shime 2011	6	40	16	270	0.4%	2.80 [1.03, 7.65]
Shorr 2014	54	93	11	38	0.5%	3.40 [1.51, 7.66]
Son 2020	77	214	116	930	0.7%	3.94 [2.81, 5.54]
Tam 2010	6	16	14	93	0.3%	3.39 [1.06, 10.81]
Valles 2003	34	49	107	290	0.6%	3.88 [2.02, 7.45]
Xie 2017	81	240	111	612	0.7%	2.30 [1.64, 3.22]
Yamaga & Shime 2017	14	16	16	46	0.2%	13.13 [2.65, 65.07]
Yang 2013	72	131	32	91	0.6%	2.25 [1.30, 3.90]
Zhang 2017	3	13	41	213	0.3%	1.26 [0.33, 4.78]
Ziberberg 2014	106	245	205	819	0.7%	2.28 [1.70, 3.08]
**Subtotal (95% CI)**		**10132**		**23422**	**36.2%**	**1.95 [1.72, 2.22]**
Total events	3312		4170			
Heterogeneity: Tau² = 0.17; Chi² = 244.69, df = 67 (P < 0.00001); I² = 73%						
Test for overall effect: Z = 10.29 (P < 0.00001)						

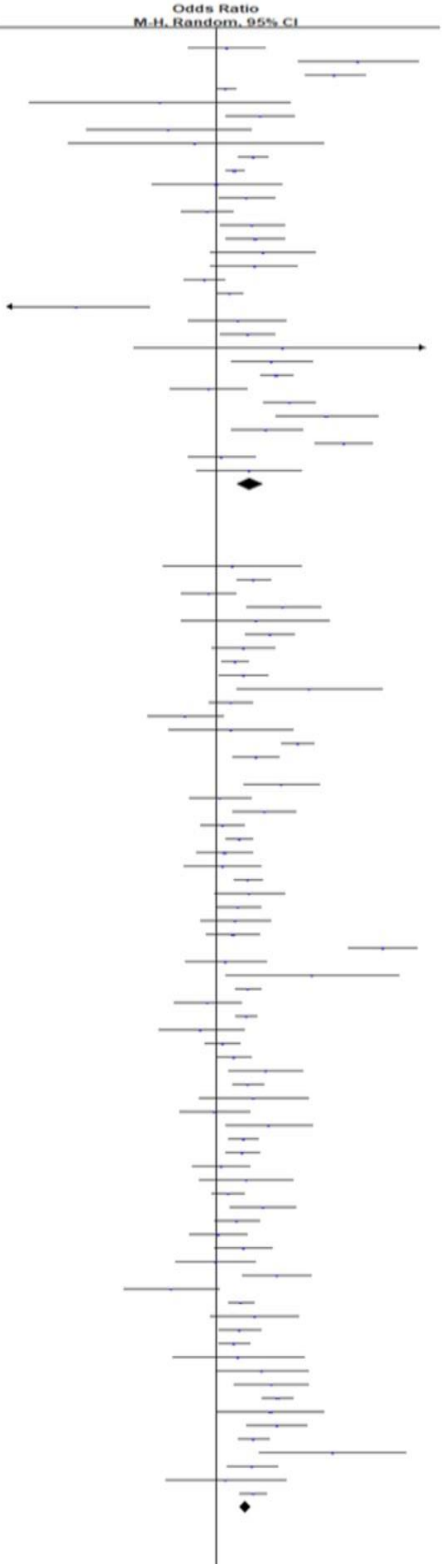

(2J) Varied EAT timeliness (continued)

1.11.3 48 hours

Cain 2015	9	31	44	359	0.5%	2.93 [1.27, 6.77]
Daikos 2009	9	29	22	133	0.4%	2.27 [0.91, 5.64]
Erbay 2009	39	60	17	43	0.5%	2.84 [1.26, 6.38]
Evans 2009	8	88	8	147	0.4%	1.74 [0.63, 4.81]
Fang 2006	36	119	23	43	0.5%	0.38 [0.18, 0.77]
Garnacho-Montero 2013	44	122	23	66	0.6%	1.05 [0.56, 1.97]
Gasch 2013	31	201	18	378	0.6%	3.65 [1.98, 6.70]
Guilarde 2006	22	44	17	67	0.5%	2.94 [1.31, 6.59]
Horino 2011	13	63	15	81	0.5%	1.14 [0.50, 2.62]
Kim 2006	45	117	34	121	0.6%	1.60 [0.93, 2.76]
Kuo 2012	13	131	12	135	0.5%	1.13 [0.50, 2.57]
Kuo SH 2018	100	275	37	153	0.7%	1.79 [1.15, 2.79]
Labelle AJ 2012	34	145	38	100	0.6%	0.50 [0.29, 0.87]
Lee 2014	75	121	25	177	0.6%	9.91 [5.66, 17.36]
Lee 2015	24	41	31	148	0.5%	5.33 [2.55, 11.13]
Lee 2017	100	432	239	1917	0.7%	2.11 [1.63, 2.75]
Lee 2019	121	475	351	2591	0.8%	2.18 [1.72, 2.76]
Lee NY 2012	63	177	12	91	0.5%	3.64 [1.84, 7.19]
Leibovici 1998	432	1255	436	2158	0.8%	2.07 [1.77, 2.43]
Liu 2018	2	11	4	25	0.2%	1.17 [0.18, 7.56]
Lodise 2003	16	48	23	119	0.5%	2.09 [0.98, 4.43]
Lodise 2007	21	48	10	52	0.4%	3.27 [1.33, 7.99]
Migiyama 2016	23	49	17	77	0.5%	3.12 [1.43, 6.80]
Osih 2007	26	53	35	114	0.5%	2.17 [1.11, 4.25]
Park 2012	24	35	27	65	0.4%	3.07 [1.29, 7.31]
Park SY 2015	17	77	9	32	0.4%	0.72 [0.28, 1.85]
Paul 2010	168	342	56	168	0.7%	1.93 [1.31, 2.84]
Reisfeld 2011	70	151	76	227	0.7%	1.72 [1.13, 2.62]
Rodriguez-Pardo 2014	22	134	15	157	0.5%	1.86 [0.92, 3.75]
Rong 2012	21	46	12	72	0.5%	4.20 [1.80, 9.82]
Saliba 2017	27	215	49	331	0.6%	0.83 [0.50, 1.37]
Satlin 2017	31	66	28	55	0.5%	0.85 [0.42, 1.75]
Seo 2020	16	79	16	54	0.5%	0.60 [0.27, 1.34]
Shargian-Alon 2019	22	24	11	22	0.2%	11.00 [2.07, 58.52]
Shih 2005	20	41	9	30	0.4%	2.22 [0.82, 5.99]
Song 2011	11	15	4	13	0.2%	6.19 [1.20, 31.97]
Trecarichi 2009	7	11	5	51	0.2%	16.10 [3.46, 74.84]
Tuon 2011	20	49	28	55	0.5%	0.67 [0.31, 1.45]
Wang X 2017	10	20	3	20	0.2%	5.67 [1.25, 25.61]
Wu 2012	9	38	4	24	0.3%	1.55 [0.42, 5.74]
Wu 2015	70	325	11	157	0.5%	3.64 [1.87, 7.10]
Yoon 2016	63	191	51	154	0.7%	0.99 [0.63, 1.56]
Zarkotou 2011	13	39	5	14	0.3%	0.90 [0.25, 3.24]
**Subtotal (95% CI)**		**6033**		**10996**	**21.1%**	**1.92 [1.59, 2.32]**
Total events	1947		1910			

Heterogeneity: Tau^2 = 0.25; Chi^2 = 174.73, df = 42 (P < 0.00001); I^2 = 76%
Test for overall effect: Z = 6.72 (P < 0.00001)

1.11.4 72 hours

Bassetti 2007	25	31	40	87	0.4%	4.90 [1.83, 13.12]
Falagas 2006	13	22	6	18	0.3%	2.89 [0.79, 10.57]
Falcone 2014	15	52	20	42	0.5%	0.45 [0.19, 1.05]
Hsieh 2015	15	34	43	212	0.5%	3.10 [1.46, 6.60]
Hung 2005	2	9	4	26	0.2%	1.57 [0.24, 10.49]
Lin 2010	17	23	6	17	0.3%	5.19 [1.33, 20.28]
Molina 2013	6	36	0	60	0.1%	25.79 [1.41, 472.98]
Park 2013	27	76	17	73	0.5%	1.82 [0.89, 3.72]
Rosa 2011	6	17	11	74	0.3%	3.12 [0.96, 10.20]
**Subtotal (95% CI)**		**300**		**609**	**3.0%**	**2.49 [1.34, 4.62]**
Total events	126		147			

Heterogeneity: Tau^2 = 0.52; Chi^2 = 21.98, df = 8 (P = 0.005); I^2 = 64%
Test for overall effect: Z = 2.87 (P = 0.004)

1.11.5 5 days

Cheng 2016	34	60	11	51	0.5%	4.76 [2.05, 11.02]
Park 2018	80	104	23	42	0.5%	2.75 [1.29, 5.89]
Ye 2006	33	68	17	58	0.5%	2.27 [1.09, 4.76]
**Subtotal (95% CI)**		**232**		**151**	**1.5%**	**3.00 [1.92, 4.69]**
Total events	147		51			

Heterogeneity: Tau^2 = 0.00; Chi^2 = 1.74, df = 2 (P = 0.42); I^2 = 0%
Test for overall effect: Z = 4.80 (P < 0.00001)

1.11.6 before BC

Al-Dorzi 2015	21	24	33	36	0.2%	0.64 [0.12, 3.45]
Ammerlaan 2009	20	94	60	240	0.6%	0.81 [0.46, 1.44]
Asgeirsson 2011	9	63	36	262	0.5%	1.05 [0.48, 2.30]
Bassetti 2012	21	56	14	109	0.5%	4.07 [1.87, 8.88]
Bassetti 2018	21	45	20	70	0.5%	2.19 [1.00, 4.78]
Bodro 2014	66	268	187	880	0.7%	1.21 [0.88, 1.67]
Carratala 1998	3	5	22	35	0.2%	0.89 [0.13, 6.02]
Chang 2009	6	33	3	55	0.2%	3.85 [0.89, 16.62]
Chusri 2019	7	34	18	26	0.3%	0.12 [0.04, 0.37]
Dimopoulos 2015	75	203	21	85	0.6%	1.79 [1.01, 3.16]
Endimiani 2003	48	207	11	314	0.5%	8.32 [4.20, 16.46]
Girometti 2014	40	124	18	93	0.6%	1.98 [1.05, 3.75]
Gradel 2017	224	1290	571	3778	0.8%	1.18 [1.00, 1.40]
Gudiol 2011	20	59	62	290	0.6%	1.89 [1.03, 3.46]
Gudiol 2013	7	33	19	59	0.4%	0.57 [0.21, 1.54]
Gudiol 2016	6	9	19	46	0.2%	2.84 [0.63, 12.80]
Haruki 2018	7	14	14	87	0.3%	5.21 [1.58, 17.20]
Ibrahim 2000	91	147	98	345	0.7%	4.10 [2.73, 6.15]
Jung 2012	13	40	84	513	0.5%	2.46 [1.22, 4.96]
Kleinhendler 2018	9	14	9	65	0.3%	11.20 [3.05, 41.10]
Lee 2011	0	29	2	135	0.1%	0.91 [0.04, 19.35]
Lewis 2011	27	112	11	63	0.5%	1.50 [0.69, 3.28]
Li 2017	7	25	19	79	0.4%	1.23 [0.45, 3.39]
Mackenzie 2003	6	21	7	54	0.3%	2.69 [0.78, 9.24]
Marcos 2008	10	82	27	232	0.5%	1.05 [0.49, 2.29]
Martinez 2010	79	454	388	4409	0.7%	2.18 [1.68, 2.84]
Ortega 2007	14	38	12	162	0.4%	7.29 [3.01, 17.64]
Ortega 2009	100	349	340	4409	0.7%	4.81 [3.72, 6.22]
Ortega 2011	14	86	82	824	0.6%	1.76 [0.95, 3.26]
Ortega 2012	20	131	106	1242	0.6%	1.93 [1.15, 3.24]
Ortega 2013	16	155	46	643	0.6%	1.49 [0.82, 2.72]
Rebelo 2011	24	89	6	46	0.4%	2.46 [0.93, 6.54]
Retamar 2012	49	199	99	602	0.7%	1.66 [1.13, 2.45]
Ruiz-Giardin 2013	14	71	24	226	0.5%	2.07 [1.00, 4.25]
Salonen 1997	9	29	5	28	0.3%	2.07 [0.60, 7.20]
Sancho 2012	106	155	114	216	0.7%	1.94 [1.26, 2.98]
Soriano 2000	33	189	77	719	0.7%	1.76 [1.13, 2.75]
Soriano 2008	73	246	43	168	0.7%	1.23 [0.79, 1.91]
Su 2007	23	57	27	66	0.5%	0.98 [0.47, 2.01]
Tagashira 2017	17	133	21	440	0.5%	2.92 [1.49, 5.72]
Takesue 2011	41	106	3	22	0.3%	3.99 [1.11, 14.35]
Tumbarello 2008	29	56	9	73	0.4%	7.64 [3.19, 18.28]
Tumbarello 2011	20	37	16	69	0.5%	3.90 [1.66, 9.16]
Tumbarello 2012	16	21	14	78	0.3%	14.63 [4.59, 46.61]
Vitkauskiene 2010	18	31	29	49	0.4%	0.95 [0.38, 2.38]
Wang 2011	15	63	12	50	0.4%	0.99 [0.41, 2.36]
Wang W 2017	19	47	16	91	0.5%	3.18 [1.44, 7.04]
Wareham 2008	10	74	7	88	0.4%	1.81 [0.65, 5.01]
Zaragoza 2003	22	39	64	127	0.5%	1.27 [0.62, 2.62]
**Subtotal (95% CI)**		**5886**		**22798**	**23.4%**	**2.02 [1.66, 2.46]**
Total events	1545		2945			

Heterogeneity: Tau^2 = 0.32; Chi^2 = 232.75, df = 48 (P < 0.00001); I^2 = 79%
Test for overall effect: Z = 6.97 (P < 0.00001)

**Total (95% CI)**		**26863**		**66984**	**100.0%**	**2.02 [1.85, 2.20]**
Total events	8650		11273			

Heterogeneity: Tau^2 = 0.25; Chi^2 = 903.60, df = 203 (P < 0.00001); I^2 = 78%
Test for overall effect: Z = 15.67 (P < 0.00001)
Test for subgroup differences: Chi^2 = 4.15, df = 5 (P = 0.53), I^2 = 0%

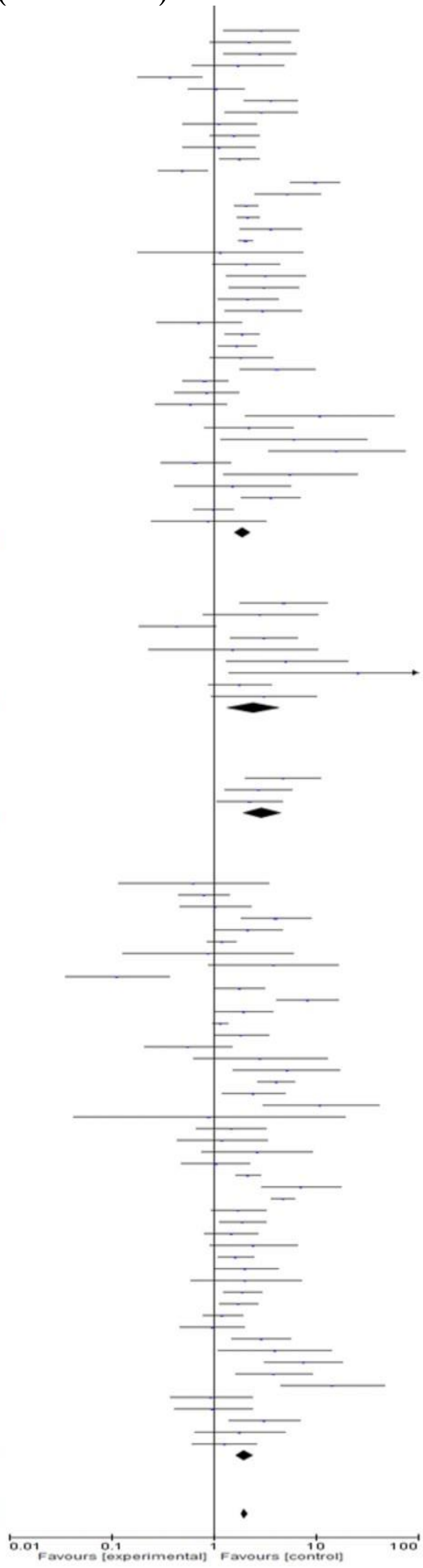

(2K) Varied mortality deadline

Study or Subgroup	Inappropriate Events	Inappropriate Total	Appropriate Events	Appropriate Total	Weight	Odds Ratio M-H, Random, 95% CI
**1.12.1 7 days**						
Bassetti 2018	14	46	10	72	0.4%	2.71 [1.08, 6.78]
Bodey 1986	50	62	129	559	0.5%	13.89 [7.18, 26.87]
Enoch 2011	13	40	11	163	0.4%	6.65 [2.70, 16.38]
Gasch 2013	31	201	18	378	0.5%	3.65 [1.98, 6.70]
Kleinhendler 2018	6	14	3	65	0.2%	15.50 [3.23, 74.45]
Marin 2014	36	120	11	408	0.5%	15.47 [7.57, 31.62]
Park 2012	10	35	11	65	0.4%	1.96 [0.74, 5.23]
Shargian-Alon 2019	22	24	11	22	0.2%	11.00 [2.07, 58.52]
Soriano 2000	33	189	77	719	0.6%	1.76 [1.13, 2.75]
Zhang 2017	12	13	13	213	0.1%	184.62 [22.25, 1531.49]
**Subtotal (95% CI)**		**744**		**2664**	**4.0%**	**6.83 [3.40, 13.73]**
Total events	227		294			
Heterogeneity: $Tau^2 = 0.98$; $Chi^2 = 60.33$, $df = 9$ ($P < 0.00001$); $I^2 = 85\%$						
Test for overall effect: $Z = 5.39$ ($P < 0.00001$)						
**1.12.3 14 days**						
Abhilash 2010	13	47	17	74	0.4%	1.28 [0.55, 2.96]
Babar 2021	16	38	3	99	0.3%	23.27 [6.23, 86.88]
Daikos 2009	9	29	22	133	0.4%	2.27 [0.91, 5.64]
Falagas 2006	11	22	4	18	0.3%	3.50 [0.87, 14.06]
Kim 2014	7	38	45	296	0.4%	1.26 [0.52, 3.03]
Kuo 2012	13	131	12	135	0.4%	1.13 [0.50, 2.57]
Kuo SH 2018	100	275	37	153	0.6%	1.79 [1.15, 2.79]
Lee NY 2012	63	177	12	91	0.5%	3.64 [1.84, 7.19]
Lin 2004	7	16	15	70	0.3%	2.85 [0.91, 8.92]
Lin 2010	17	23	6	17	0.3%	5.19 [1.33, 20.28]
Metan 2009	53	76	10	24	0.4%	3.23 [1.25, 8.32]
Park 2012	16	35	14	65	0.4%	3.07 [1.26, 7.47]
Park 2013	27	76	17	73	0.5%	1.82 [0.89, 3.72]
Park 2018	76	104	20	42	0.5%	2.99 [1.42, 6.29]
Retamar 2012	49	199	99	602	0.7%	1.66 [1.13, 2.45]
Retamar 2013	16	57	53	284	0.5%	1.70 [0.89, 3.26]
Rodriguez-Bano J 2010	17	77	14	205	0.5%	3.87 [1.80, 8.30]
Seo 2020	16	79	16	54	0.5%	0.60 [0.27, 1.34]
Shih 2005	20	41	9	30	0.4%	2.22 [0.82, 5.99]
Wang 2011	15	63	12	50	0.4%	0.99 [0.41, 2.36]
**Subtotal (95% CI)**		**1603**		**2515**	**8.7%**	**2.12 [1.63, 2.77]**
Total events	561		437			
Heterogeneity: $Tau^2 = 0.18$; $Chi^2 = 41.68$, $df = 19$ ($P = 0.002$); $I^2 = 54\%$						
Test for overall effect: $Z = 5.58$ ($P < 0.00001$)						
**1.12.4 21 days**						
Bassetti 2007	25	31	40	87	0.4%	4.90 [1.83, 13.12]
Falcone 2014	15	52	20	42	0.4%	0.45 [0.19, 1.05]
Rosa 2011	6	17	11	74	0.3%	3.12 [0.96, 10.20]
Trecarichi 2016	79	152	22	126	0.6%	5.12 [2.92, 8.95]
Tumbarello 2008	29	56	9	73	0.4%	7.64 [3.19, 18.28]
Tumbarello 2010	11	27	6	107	0.3%	11.57 [3.75, 35.68]
Tumbarello 2011	20	37	16	69	0.4%	3.90 [1.66, 9.16]
Tumbarello 2013	16	21	14	78	0.3%	14.63 [4.59, 46.61]
**Subtotal (95% CI)**		**393**		**656**	**3.2%**	**4.41 [2.11, 9.19]**
Total events	201		138			
Heterogeneity: $Tau^2 = 0.89$; $Chi^2 = 36.92$, $df = 7$ ($P < 0.00001$); $I^2 = 81\%$						
Test for overall effect: $Z = 3.96$ ($P < 0.0001$)						

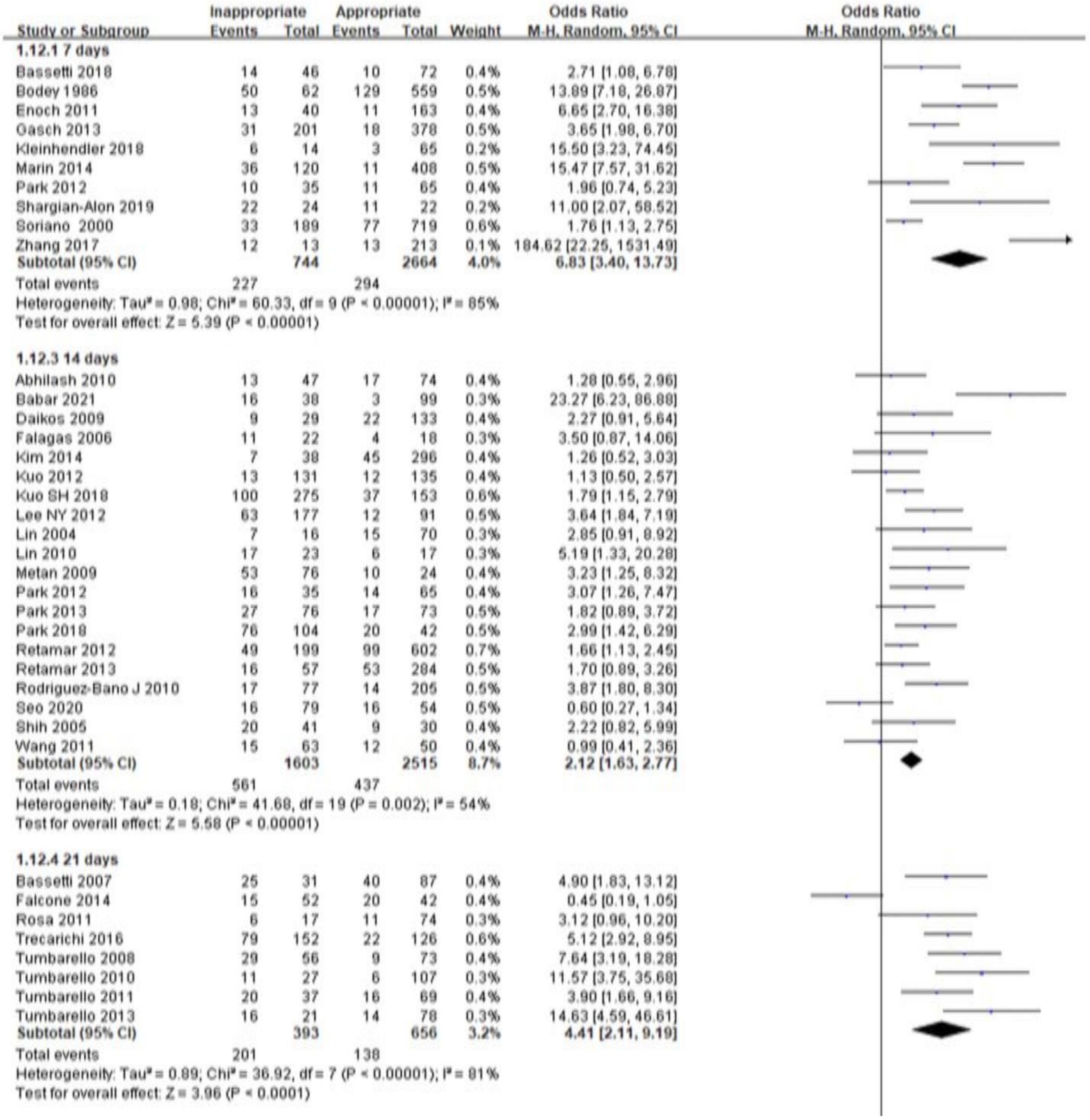

(2K) Varied mortality deadline (continued)

**1.12.5 28-30days**						
Adrie 2017	77	153	127	418	0.7%	2.32 [1.59, 3.39]
Alvarez-Marin 2020	21	117	34	168	0.6%	0.86 [0.47, 1.58]
Ammerlaan 2009	20	94	60	240	0.6%	0.81 [0.46, 1.44]
Asgeirsson 2011	9	63	36	262	0.5%	1.05 [0.48, 2.30]
Bang 2013	10	43	2	18	0.2%	2.42 [0.47, 12.39]
Bartoletti 2014	48	122	31	190	0.6%	3.33 [1.96, 5.65]
Bartoletti 2018	23	62	24	100	0.5%	1.87 [0.94, 3.72]
Bassetti 2012	21	56	14	109	0.5%	4.07 [1.87, 8.88]
Bassetti 2018	21	45	20	70	0.5%	2.19 [1.00, 4.78]
Bodro 2014	66	268	187	880	0.7%	1.21 [0.88, 1.67]
Boel 2015	79	306	313	1702	0.7%	1.54 [1.16, 2.05]
Cain 2015	9	31	44	359	0.4%	2.93 [1.27, 6.77]
Calle 2016	14	38	32	60	0.4%	0.51 [0.22, 1.17]
Carratala 1998	3	5	22	35	0.2%	0.89 [0.13, 6.02]
Chamot 2003	3	17	13	98	0.3%	1.40 [0.35, 5.55]
Chen 2013	97	255	62	682	0.7%	6.14 [4.27, 8.83]
Cheng 2016	34	60	11	51	0.4%	4.76 [2.05, 11.02]
Cheong 2008	29	106	68	508	0.6%	2.44 [1.48, 4.01]
Chusri 2019	7	34	18	26	0.3%	0.12 [0.04, 0.37]
Davis 2014	0	0	4	41		Not estimable
Dimopoulos 2015	75	203	21	85	0.6%	1.79 [1.01, 3.16]
Enoch 2011	15	40	30	163	0.5%	2.66 [1.25, 5.65]
Erbay 2009	39	60	17	43	0.5%	2.84 [1.26, 6.38]
Evans 2009	8	88	8	147	0.4%	1.74 [0.63, 4.81]
Falcone 2020	30	47	16	55	0.4%	4.30 [1.87, 9.89]
Fang 2006	36	119	23	43	0.5%	0.38 [0.18, 0.77]
Feodoroff 2011	1	46	1	30	0.1%	0.64 [0.04, 10.71]
Frakking 2013	30	147	16	85	0.5%	1.11 [0.56, 2.17]
Gasch 2013	72	197	107	382	0.7%	1.48 [1.03, 2.13]
Girometti 2014	40	124	18	93	0.5%	1.98 [1.05, 3.75]
Gradel 2017	224	1290	571	3778	0.7%	1.18 [1.00, 1.40]
Gudiol 2011	20	59	62	290	0.5%	1.89 [1.03, 3.46]
Gudiol 2013	7	33	19	59	0.4%	0.57 [0.21, 1.54]
Gudiol 2016	6	9	19	46	0.2%	2.84 [0.63, 12.80]
Haruki 2018	7	14	14	87	0.3%	5.21 [1.58, 17.20]
Hernandez 2014	28	189	72	556	0.6%	1.17 [0.73, 1.87]
Hernandez 2015	67	404	231	2201	0.7%	1.70 [1.26, 2.28]
Horcajada 2012	15	127	53	540	0.5%	1.23 [0.67, 2.26]
Horino 2011	14	63	14	71	0.4%	1.16 [0.51, 2.68]
Hsieh 2015	15	34	43	212	0.5%	3.10 [1.46, 6.60]
Hung 2005	2	9	4	26	0.2%	1.57 [0.24, 10.49]
Jung 2012	13	40	84	513	0.5%	2.46 [1.22, 4.96]
Kang 2003	40	89	13	47	0.5%	2.14 [0.99, 4.58]
Kang 2005	58	151	37	135	0.6%	1.65 [1.00, 2.72]
Kang 2011	10	64	32	301	0.5%	1.56 [0.72, 3.35]
Kang 2013	7	64	3	28	0.2%	1.02 [0.24, 4.28]
Kang Wi 2013	27	257	22	299	0.6%	1.48 [0.82, 2.66]
Kleinhendler 2018	9	14	9	65	0.3%	11.20 [3.05, 41.10]
Klevay 2009	21	78	75	244	0.6%	0.83 [0.47, 1.47]
Ku 2011	6	8	5	19	0.2%	8.40 [1.26, 56.07]
Lee 2013	32	63	21	67	0.5%	2.26 [1.11, 4.62]
Lee 2014	75	121	25	177	0.6%	9.91 [5.66, 17.36]
Lee 2015	24	41	31	148	0.5%	5.33 [2.55, 11.13]
Lee 2017	100	432	239	1917	0.7%	2.11 [1.63, 2.75]
Lee 2019	136	603	336	2591	0.7%	1.95 [1.56, 2.44]
Lee CC 2012	19	124	23	330	0.5%	2.42 [1.26, 4.61]
Lewis 2011	27	112	11	63	0.5%	1.50 [0.69, 3.28]
Li 2017	7	25	19	79	0.4%	1.23 [0.45, 3.39]
Lim 2019	10	57	12	53	0.4%	0.73 [0.28, 1.86]
Lin 2008	50	540	79	983	0.7%	1.17 [0.81, 1.69]
Liu 2018	2	11	4	25	0.2%	1.17 [0.18, 7.56]
Lodise 2007	21	48	10	52	0.4%	3.27 [1.33, 7.99]
Lye 2012	77	294	73	381	0.7%	1.50 [1.04, 2.16]
Ma 2017	14	39	20	129	0.5%	3.05 [1.36, 6.86]
Man 2017	100	364	76	489	0.7%	2.06 [1.47, 2.88]
Marcos 2008	10	82	27	232	0.5%	1.05 [0.49, 2.29]
Marin 2014	123	414	40	114	0.6%	0.78 [0.50, 1.21]
Martinez 2010	79	454	388	4409	0.7%	2.18 [1.68, 2.84]
Martinez-Nadal 2020	99	394	240	1221	0.7%	1.37 [1.05, 1.79]
Metan 2005	5	41	9	12	0.2%	0.05 [0.01, 0.23]
Migiyama 2016	23	49	17	77	0.5%	3.12 [1.43, 6.80]
Molina 2013	1	13	0	18	0.1%	4.44 [0.17, 118.00]
Morata 2012	65	246	76	463	0.7%	1.83 [1.26, 2.66]
Ortega 2009	100	349	340	4409	0.7%	4.81 [3.72, 6.22]
Ortega 2011	14	86	82	824	0.5%	1.76 [0.95, 3.26]
Ortega 2012	20	131	106	1242	0.6%	1.93 [1.15, 3.24]
Ortega 2013	16	155	46	643	0.6%	1.49 [0.82, 2.72]
Park 2011	7	42	40	108	0.4%	0.34 [0.14, 0.84]
Park 2012	24	35	27	65	0.4%	3.07 [1.29, 7.31]
Park 2018	80	104	23	42	0.5%	2.75 [1.29, 5.89]
Park H 2015	21	48	10	54	0.4%	3.42 [1.40, 8.35]
Park SY 2015	17	77	9	32	0.4%	0.72 [0.28, 1.85]
Paul 2010	168	342	56	168	0.7%	1.93 [1.31, 2.84]
Pena 2013	86	261	90	332	0.7%	1.32 [0.93, 1.88]
Picot-Gueraud 2015	21	91	30	137	0.5%	1.07 [0.57, 2.02]
Reisfeld 2011	70	151	76	227	0.6%	1.72 [1.13, 2.62]
Retamar 2012	49	199	99	602	0.7%	1.66 [1.13, 2.45]
Retamar 2013	17	57	53	284	0.5%	1.85 [0.98, 3.52]
Rodriguez-Bano 2010	13	43	16	53	0.4%	1.00 [0.42, 2.41]
Rodriguez-Pardo 2014	22	134	15	157	0.5%	1.86 [0.92, 3.75]
Royo-Cebrecos 2017	9	38	36	135	0.4%	0.85 [0.37, 1.98]
Ruiz-Giardin 2013	14	71	24	226	0.5%	2.07 [1.00, 4.25]
Saliba 2017	27	215	49	331	0.6%	0.83 [0.50, 1.37]
Satlin 2017	31	66	28	55	0.5%	0.85 [0.42, 1.75]
Schweizer 2010	77	277	109	537	0.7%	1.51 [1.08, 2.12]
Son 2020	77	214	116	930	0.7%	3.94 [2.81, 5.54]
Song 2011	11	15	4	13	0.2%	6.19 [1.20, 31.97]
Soriano 2008	73	246	43	168	0.6%	1.23 [0.79, 1.91]
Tagashira 2017	17	133	21	440	0.5%	2.92 [1.49, 5.72]
Takesue 2011	41	106	3	22	0.3%	3.99 [1.11, 14.35]
Tam 2010	6	16	14	93	0.3%	3.39 [1.06, 10.81]
Trecarichi 2009	7	11	5	51	0.2%	16.10 [3.46, 74.84]
Tumbarello 2012	39	75	13	50	0.5%	3.08 [1.42, 6.71]
Tuon 2011	20	49	28	55	0.5%	0.67 [0.31, 1.45]
Vitkauskiene 2010	18	31	29	49	0.4%	0.95 [0.38, 2.38]
Wang X 2017	10	20	3	20	0.2%	5.67 [1.25, 25.61]
Wareham 2008	10	74	7	88	0.4%	1.81 [0.65, 5.01]
Wu 2012	9	38	4	24	0.3%	1.55 [0.42, 5.74]
Wu 2015	70	325	11	157	0.5%	3.64 [1.87, 7.10]
Xie 2017	81	240	111	612	0.7%	2.30 [1.64, 3.22]
Yamaga & Shime 2017	10	16	14	46	0.3%	3.81 [1.16, 12.54]
Ye 2006	33	68	17	58	0.5%	2.27 [1.09, 4.76]
Yilmaz 2016	12	72	27	183	0.5%	1.16 [0.55, 2.43]
Zhang 2017	13	13	41	213	0.1%	112.23 [6.54, 1926.54]
**Subtotal (95% CI)**		15243		44625	54.0%	1.79 [1.59, 2.00]
Total events	4092		6412			

Heterogeneity: $Tau^2 = 0.24$; $Chi^2 = 480.97$, $df = 112$ ($P < 0.00001$); $I^2 = 77\%$
Test for overall effect: $Z = 9.89$ ($P < 0.00001$)

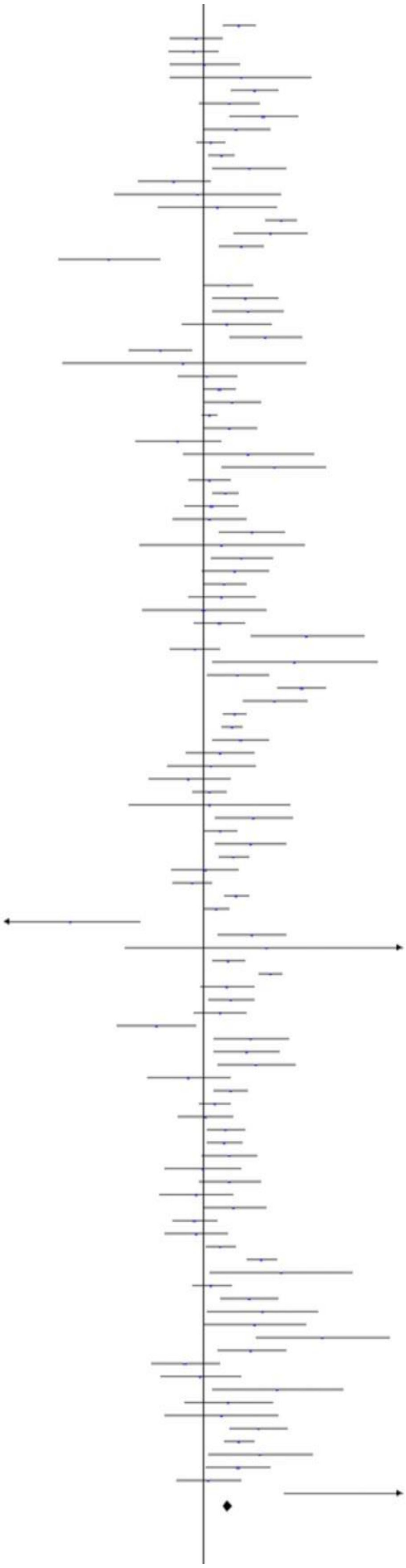

(2K) Varied mortality deadline (continued)

**1.12.6 In-hospital**						
Abraham 2016	3	12	8	43	0.2%	1.46 [0.32, 6.64]
Al-Dorzi 2015	21	24	33	36	0.2%	0.64 [0.12, 3.45]
Anatoliotaki 2004	18	46	14	111	0.5%	4.45 [1.97, 10.06]
Bouza 2004	37	120	33	170	0.6%	1.85 [1.08, 3.18]
Bouza 2005	10	12	25	65	0.2%	8.00 [1.62, 39.56]
Byl 1999	37	159	48	269	0.6%	1.40 [0.86, 2.26]
Chang 2009	6	33	3	55	0.2%	3.85 [0.89, 16.62]
Corona 2010	415	1013	244	689	0.7%	1.27 [1.04, 1.55]
Elhanan 1997	0	10	17	124	0.1%	0.29 [0.02, 5.22]
Endimiani 2003	48	207	11	314	0.5%	8.32 [4.20, 16.46]
Evans 2009	26	88	10	147	0.5%	5.75 [2.61, 12.64]
Falagas 2006	13	22	6	18	0.3%	2.89 [0.79, 10.57]
Feldman 1990	2	6	24	41	0.2%	0.35 [0.06, 2.16]
Frakking 2013	30	147	24	85	0.5%	0.65 [0.35, 1.21]
Garey 2006	24	62	23	130	0.5%	2.94 [1.49, 5.81]
Garnacho-Montero 2013	44	122	23	66	0.5%	1.05 [0.56, 1.97]
Guilarde 2006	22	44	17	67	0.5%	2.94 [1.31, 6.59]
Guillamet 2016	100	187	282	844	0.7%	2.29 [1.66, 3.16]
Ibrahim 2000	91	147	98	345	0.6%	4.10 [2.73, 6.15]
Johnson 2011	125	235	185	519	0.7%	2.05 [1.50, 2.81]
Khan 2010	55	65	47	387	0.5%	39.79 [18.99, 83.36]
Khatib 2006	21	60	60	282	0.6%	1.99 [1.09, 3.64]
Labelle A 2012	321	470	224	436	0.7%	2.04 [1.56, 2.67]
Labelle AJ 2012	60	208	12	37	0.5%	0.84 [0.40, 1.79]
Lee 2011	3	29	2	135	0.2%	7.67 [1.22, 48.21]
Leibovici 1998	432	1255	436	2158	0.7%	2.07 [1.77, 2.43]
Lin 2006	12	41	10	67	0.4%	2.36 [0.91, 6.10]
Lodise 2003	16	48	23	119	0.5%	2.09 [0.98, 4.43]
Mackenzie 2003	6	21	7	54	0.3%	2.69 [0.78, 9.24]
Marra 2006	6	27	7	64	0.3%	2.33 [0.70, 7.72]
Marschall 2008	11	79	24	171	0.5%	0.99 [0.46, 2.14]
Meyers 1989	8	16	32	84	0.4%	1.63 [0.55, 4.76]
Micek & Lloyd 2010	23	75	41	230	0.6%	2.04 [1.12, 3.70]
Micek 2010	270	522	87	238	0.7%	1.86 [1.36, 2.55]
Ortega 2007	14	38	12	162	0.4%	7.29 [3.01, 17.64]
Osih 2007	26	68	35	99	0.5%	1.13 [0.60, 2.15]
Peralta 2007	12	106	24	557	0.5%	2.84 [1.37, 5.86]
Peralta 2012	47	189	24	198	0.6%	2.40 [1.40, 4.12]
Phillips 1990	67	159	215	1346	0.7%	3.83 [2.71, 5.42]
Rebelo 2011	24	89	6	46	0.4%	2.46 [0.93, 6.54]
Rong 2012	21	46	12	72	0.4%	4.20 [1.80, 9.82]
Salonen 1997	9	29	5	28	0.3%	2.07 [0.60, 7.20]
Sancho 2012	106	155	114	216	0.6%	1.94 [1.26, 2.98]
Savage 2016	135	266	341	924	0.7%	1.76 [1.34, 2.32]
Schechner 2011	15	32	12	44	0.4%	2.35 [0.90, 6.15]
Schramm 2006	99	390	28	169	0.6%	1.71 [1.08, 2.73]
Shime 2011	6	40	16	270	0.4%	2.80 [1.03, 7.65]
Shorr 2014	54	93	11	38	0.5%	3.40 [1.51, 7.66]
Su 2007	23	57	27	66	0.5%	0.98 [0.47, 2.01]
Valles 2003	34	49	107	290	0.5%	3.88 [2.02, 7.45]
Wang 2005	50	68	43	303	0.5%	16.80 [8.96, 31.47]
Wang W 2017	19	47	16	91	0.5%	3.18 [1.44, 7.04]
Yamaga & Shime 2017	13	16	15	46	0.3%	8.96 [2.21, 36.26]
Yang 2013	72	131	32	91	0.6%	2.25 [1.30, 3.90]
Yoon 2016	63	191	51	154	0.6%	0.99 [0.63, 1.56]
Yu 2017	16	25	11	24	0.3%	2.10 [0.67, 6.60]
Zaragoza 2003	22	39	54	127	0.5%	1.75 [0.85, 3.61]
Zarkotou 2011	13	39	5	14	0.3%	0.90 [0.25, 3.24]
Ziberberg 2014	106	245	205	819	0.7%	2.28 [1.70, 3.08]
**Subtotal (95% CI)**		**8219**		**14794**	**27.8%**	**2.36 [2.01, 2.78]**
Total events	3382		3561			
Heterogeneity: Tau² = 0.25; Chi² = 265.96, df = 58 (P < 0.00001); I² = 78%						
Test for overall effect: Z = 10.44 (P < 0.00001)						
**1.12.7 long-term**						
Gradel 2017	317	1290	716	3778	0.7%	1.39 [1.20, 1.62]
Hanon 2002	279	768	351	1290	0.7%	1.53 [1.26, 1.85]
Kim 2006	45	117	34	121	0.6%	1.60 [0.93, 2.76]
Yamaga & Shime 2017	14	16	16	46	0.2%	13.13 [2.65, 65.07]
**Subtotal (95% CI)**		**2191**		**5235**	**2.2%**	**1.56 [1.21, 2.00]**
Total events	655		1117			
Heterogeneity: Tau² = 0.03; Chi² = 7.92, df = 3 (P = 0.05); I² = 62%						
Test for overall effect: Z = 3.43 (P = 0.0006)						
**Total (95% CI)**		**28393**		**70489**	**100.0%**	**2.12 [1.94, 2.31]**
Total events	9118		11959			
Heterogeneity: Tau² = 0.27; Chi² = 1001.86, df = 213 (P < 0.00001); I² = 79%						
Test for overall effect: Z = 16.81 (P < 0.00001)						
Test for subgroup differences: Chi² = 28.09, df = 5 (P < 0.0001), I² = 82.2%						

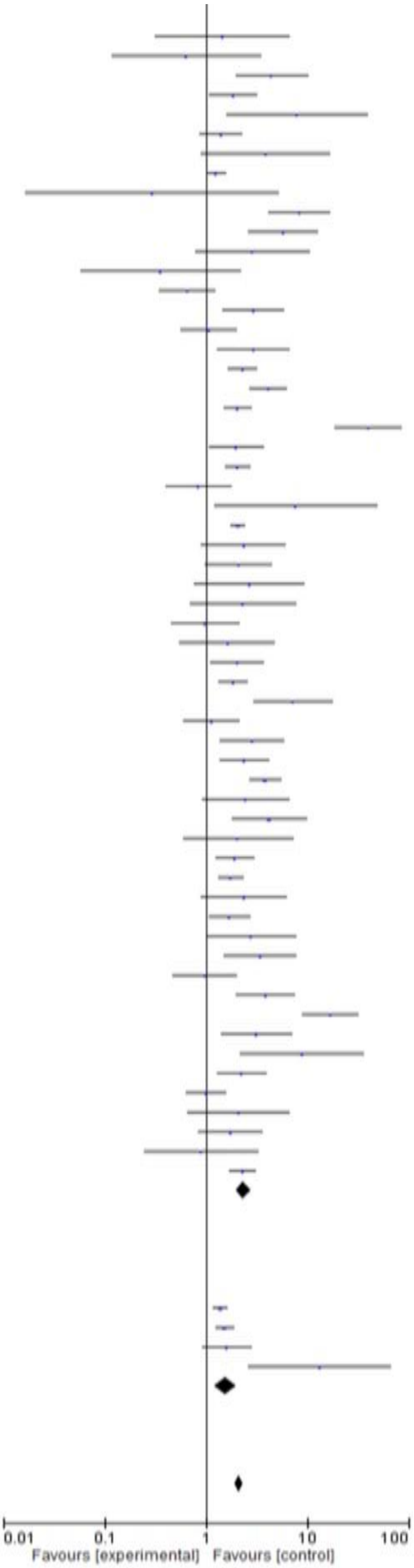

Supplemental Figure 3. Adjusted analyses in overall patients: the forest and funnel plots.

(3A) Forest plots

Study or Subgroup	log[Odds Ratio]	SE	Weight	Odds Ratio IV, Random, 95% CI
Adrie 2017	0.8329	0.1251	1.3%	2.30 [1.80, 2.94]
Al-Dorzi 2015	1.8961	0.9474	0.2%	6.66 [1.04, 42.65]
Anatoliotaki 2004	1.3863	0.3537	0.7%	4.00 [2.00, 8.00]
Bartoletti 2014	1.0716	0.3399	0.8%	2.92 [1.50, 5.68]
Bartoletti 2018	0.7608	0.0527	1.4%	2.14 [1.93, 2.37]
Bassetti 2007	1.5581	0.5213	0.5%	4.75 [1.71, 13.19]
Bassetti 2012	1.026	0.4347	0.6%	2.79 [1.19, 6.54]
Bassetti 2018	1.1939	0.5559	0.4%	3.30 [1.11, 9.81]
Boel 2015	0.239	0.1271	1.3%	1.27 [0.99, 1.63]
Bouza 2004	1.2528	0.4323	0.6%	3.50 [1.50, 8.17]
Bouza 2005	2.3609	1.1115	0.1%	10.60 [1.20, 93.63]
Byl 1999	0.7561	0.3149	0.8%	2.13 [1.15, 3.95]
Cain 2015	1.2641	0.3833	0.7%	3.54 [1.67, 7.50]
Chamot 2003	0.1823	0.7246	0.3%	1.20 [0.29, 4.97]
Chang 2009	0	0.0595	1.4%	1.00 [0.89, 1.12]
Chen 2013	0.571	0.1614	1.2%	1.77 [1.29, 2.43]
Cheng 2016	1.6601	0.542	0.4%	5.26 [1.82, 15.22]
Cheong 2008	1.2556	0.5605	0.4%	3.51 [1.17, 10.53]
Chusri 2019	-2.9957	0.8212	0.2%	0.05 [0.01, 0.25]
Corona 2010	0.1133	0.1918	1.2%	1.12 [0.77, 1.63]
Daikos 2009	0.4886	0.4461	0.6%	1.63 [0.68, 3.91]
Dimopoulos 2015	0.7324	0.3488	0.8%	2.08 [1.05, 4.12]
Elhanan 1997	0	0.2365	1.0%	1.00 [0.63, 1.59]
Endimiani 2003	1.454	0.9386	0.2%	4.28 [0.68, 26.94]
Erbay 2009	0.8755	0.3128	0.8%	2.40 [1.30, 4.43]
Evans 2009	0.5539	0.5348	0.5%	1.74 [0.61, 4.96]
Falagas 2006	0	0.0711	1.4%	1.00 [0.87, 1.15]
Falcone 2020	0.3235	0.1018	1.4%	1.38 [1.13, 1.69]
Fang 2006	-0.1863	0.1169	1.3%	0.83 [0.66, 1.04]
Garey 2006	0.4055	0.1629	1.2%	1.50 [1.09, 2.06]
Garnacho-Montero 2013	0.9163	0.3745	0.7%	2.50 [1.20, 5.21]
Gasch 2013	1.2782	0.4029	0.7%	3.59 [1.63, 7.91]
Girometti 2014	0.6419	0.2789	0.9%	1.90 [1.10, 3.28]
Gradel 2017	0.1621	0.093	1.4%	1.18 [0.98, 1.41]
Gudiol 2011	0.4511	0.5838	0.4%	1.57 [0.50, 4.93]
Guilarde 2006	0.8198	0.4082	0.6%	2.27 [1.02, 5.05]
Guillamet 2016	0.8329	0.4149	0.6%	2.30 [1.02, 5.19]
Hanon 2002	0.4447	0.1381	1.3%	1.56 [1.19, 2.04]
Hernandez 2015	0.3988	0.1884	1.2%	1.49 [1.03, 2.16]
Hsieh 2015	1.4563	0.4875	0.5%	4.29 [1.65, 11.15]
Ibrahim 2000	1.9257	0.1523	1.3%	6.86 [5.09, 9.25]
Johnson 2011	0.708	0.1027	1.4%	2.03 [1.66, 2.48]
Jung 2012	0.8879	0.4185	0.6%	2.43 [1.07, 5.52]
Kang 2003	1.5282	0.6953	0.3%	4.61 [1.18, 18.01]
Kang 2005	0	0.3144	0.8%	1.00 [0.54, 1.85]
Kang 2013	0.8109	0.3514	0.8%	2.25 [1.13, 4.48]
Khan 2010	2.1223	0.4799	0.5%	8.35 [3.26, 21.39]
Khatib 2006	0	0.2198	1.1%	1.00 [0.65, 1.54]
Kim 2006	0.3293	0.4119	0.6%	1.39 [0.62, 3.12]
Ku 2011	3.1747	1.4808	0.1%	23.92 [1.31, 435.73]
Kuo SH 2018	0.6307	0.3162	0.8%	1.88 [1.01, 3.49]
Labelle AJ 2012	0	0.6316	0.4%	1.00 [0.29, 3.45]
Lee 2011	0	0.3537	0.7%	1.00 [0.50, 2.00]
Lee 2013	1.1709	0.4298	0.6%	3.22 [1.39, 7.49]
Lee 2014	1.9544	0.3451	0.8%	7.06 [3.59, 13.88]
Lee 2015	1.914	0.4871	0.5%	6.78 [2.61, 17.61]
Lee 2017	0.9555	0.1654	1.2%	2.60 [1.88, 3.60]
Lee 2019	0.4762	0.1226	1.3%	1.61 [1.27, 2.05]
Lee CC 2012	0.8154	0.4109	0.6%	2.26 [1.01, 5.06]
Lee NY 2012	1.5129	0.4183	0.6%	4.54 [2.00, 10.31]
Leibovici 1998	0.47	0.1059	1.4%	1.60 [1.30, 1.97]
Lin 2010	1.4341	0.6831	0.3%	4.20 [1.10, 16.01]
Lodise 2003	1.335	0.6811	0.3%	3.80 [1.00, 14.44]
Lodise 2007	1.411	0.6269	0.4%	4.10 [1.20, 14.01]
Lye 2012	0.1389	0.2356	1.0%	1.15 [0.72, 1.82]
[illegible]	[illegible]	[illegible]	[illegible]	[illegible]

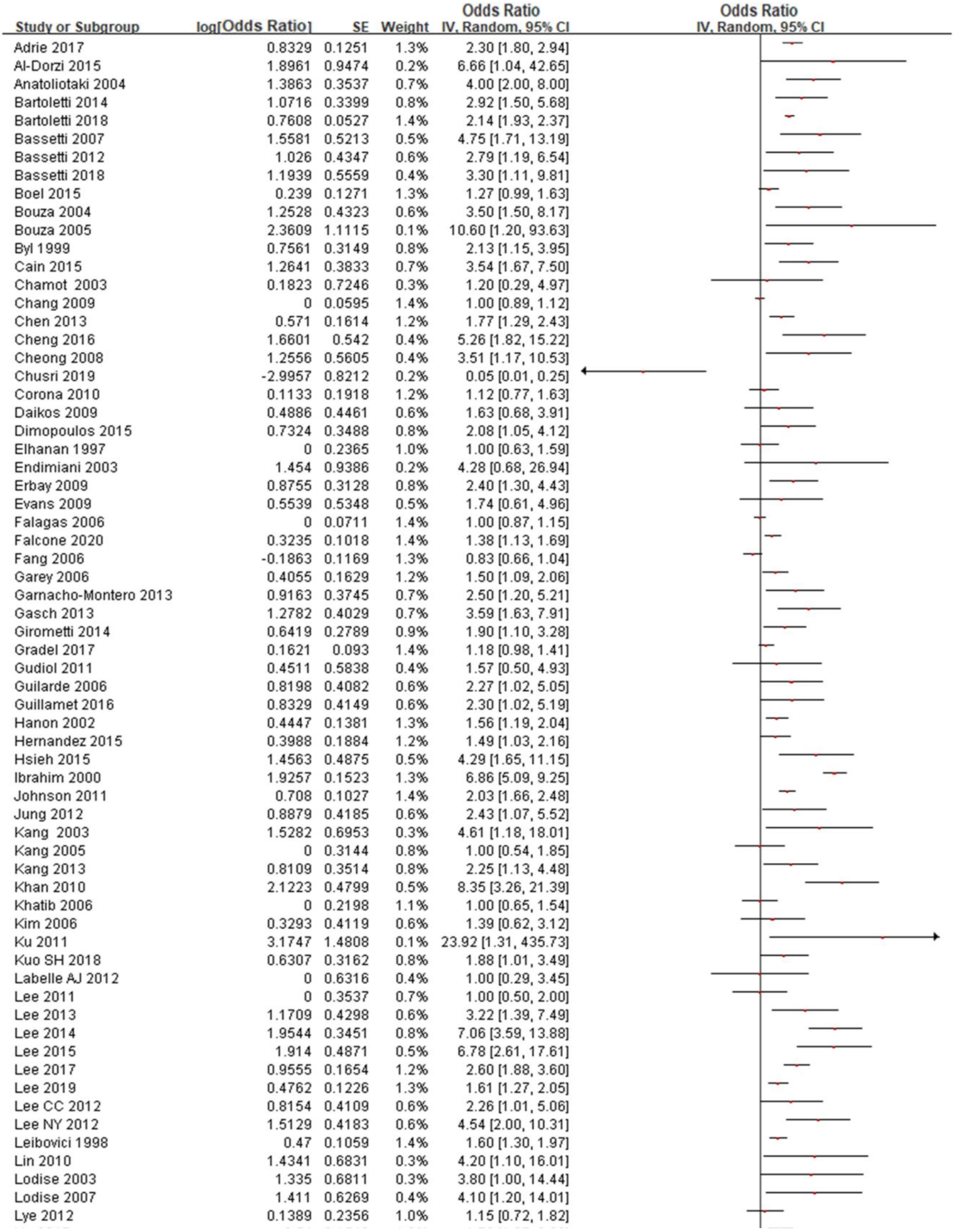

(3A) Forest plots (continued)

Ma 2017	0.54	0.1548	1.2%	1.72 [1.27, 2.32]
Martinez 2010	0.5878	0.166	1.2%	1.80 [1.30, 2.49]
Martinez-Nadal 2020	1.1053	0.434	0.6%	3.02 [1.29, 7.07]
Metan 2005	0	0		Not estimable
Metan 2009	0	0.5504	0.4%	1.00 [0.34, 2.94]
Micek & Lioyd 2010	0.7129	0.1848	1.2%	2.04 [1.42, 2.93]
Micek 2010	0.8329	0.1002	1.4%	2.30 [1.89, 2.80]
Migiyama 2016	1.5602	0.4527	0.6%	4.76 [1.96, 11.56]
Molina 2013	0	0.0829	1.4%	1.00 [0.85, 1.18]
Morata 2012	0.7793	0.2983	0.9%	2.18 [1.21, 3.91]
Ortega 2007	0.6931	0.2522	1.0%	2.00 [1.22, 3.28]
Ortega 2009	1.5748	0.1673	1.2%	4.83 [3.48, 6.70]
Ortega 2011	0	0.0262	1.5%	1.00 [0.95, 1.05]
Ortega 2012	0.3365	0.123	1.3%	1.40 [1.10, 1.78]
Ortega 2013	0.6206	0.1166	1.3%	1.86 [1.48, 2.34]
Park 2012	1.0043	0.4592	0.6%	2.73 [1.11, 6.71]
Park 2013	1.4695	0.6238	0.4%	4.35 [1.28, 14.76]
Park 2018	1.1756	0.2617	1.0%	3.24 [1.94, 5.41]
Park H 2015	1.1756	0.3929	0.7%	3.24 [1.50, 7.00]
Paul 2010	0.7655	0.2412	1.0%	2.15 [1.34, 3.45]
Pena 2013	0.5306	0.2759	0.9%	1.70 [0.99, 2.92]
Peralta 2007	1.0919	0.4433	0.6%	2.98 [1.25, 7.10]
Peralta 2012	0.94	0.4645	0.6%	2.56 [1.03, 6.36]
Rebelo 2011	0	0.0051	1.5%	1.00 [0.99, 1.01]
Reisfeld 2011	0.3436	0.2523	1.0%	1.41 [0.86, 2.31]
Retamar 2012	0.7514	0.2341	1.0%	2.12 [1.34, 3.35]
Retamar 2013	0.6931	0.3798	0.7%	2.00 [0.95, 4.21]
Rodriguez-Bano 2010	0	0.3537	0.7%	1.00 [0.50, 2.00]
Rodriguez-Bano J 2010	1.0986	0.4267	0.6%	3.00 [1.30, 6.92]
Rodriguez-Pardo 2014	1.0188	0.3868	0.7%	2.77 [1.30, 5.91]
Rong 2012	1.3863	0.5181	0.5%	4.00 [1.45, 11.04]
Sancho 2012	0.6575	0.3563	0.7%	1.93 [0.96, 3.88]
Savage 2016	0.0198	0.1921	1.1%	1.02 [0.70, 1.49]
Schechner 2011	0	0.0538	1.4%	1.00 [0.90, 1.11]
Schramm 2006	0.6523	0.1328	1.3%	1.92 [1.48, 2.49]
Schweizer 2010	0.4055	0.2494	1.0%	1.50 [0.92, 2.45]
Shargian-Alon 2019	2.9565	1.2407	0.1%	19.23 [1.69, 218.82]
Shorr 2014	0.3492	0.13	1.3%	1.42 [1.10, 1.83]
Son 2020	1.8148	0.533	0.5%	6.14 [2.16, 17.45]
Song 2011	0.7419	1.1997	0.1%	2.10 [0.20, 22.05]
Soriano 2000	0.7561	0.2885	0.9%	2.13 [1.21, 3.75]
Tagashira 2017	1.0225	0.3997	0.7%	2.78 [1.27, 6.09]
Takesue 2011	1.514	0.7248	0.3%	4.54 [1.10, 18.81]
Tam 2010	0	0.0316	1.5%	1.00 [0.94, 1.06]
Trecarichi 2009	2.7054	1.0396	0.2%	14.96 [1.95, 114.78]
Trecarichi 2016	0.6259	0.2801	0.9%	1.87 [1.08, 3.24]
Tumbarello 2008	1.8278	0.501	0.5%	6.22 [2.33, 16.61]
Tumbarello 2011	1.0043	0.4731	0.5%	2.73 [1.08, 6.90]
Tumbarello 2012	1.4279	0.4856	0.5%	4.17 [1.61, 10.80]
Tumbarello 2013	2.2875	0.9535	0.2%	9.85 [1.52, 63.84]
Valles 2003	1.1725	0.3846	0.7%	3.23 [1.52, 6.86]
Wang X 2017	0	0.3537	0.7%	1.00 [0.50, 2.00]
Wu 2012	1.4697	0.4542	0.6%	4.35 [1.79, 10.59]
Xie 2017	2.1136	0.2123	1.1%	8.28 [5.46, 12.55]
Yamaga & Shime 2017	3.1465	0.8556	0.2%	23.25 [4.35, 124.39]
Yang 2013	1.3124	0.3882	0.7%	3.72 [1.74, 7.95]
Ye 2006	0	0.5976	0.4%	1.00 [0.31, 3.23]
Yu 2017	0.1275	0.5849	0.4%	1.14 [0.36, 3.57]
Zaragoza 2003	0	0.2439	1.0%	1.00 [0.62, 1.61]
Zhang 2017	1.6094	0.3537	0.7%	5.00 [2.50, 10.00]
Ziberberg 2014	1.3538	0.1709	1.2%	3.87 [2.77, 5.41]
**Total (95% CI)**			**100.0%**	**2.02 [1.86, 2.20]**

Heterogeneity: $Tau^2 = 0.13$; $Chi^2 = 1523.69$, df = 124 ($P < 0.00001$); $I^2 = 92\%$
Test for overall effect: Z = 16.17 ($P < 0.00001$)

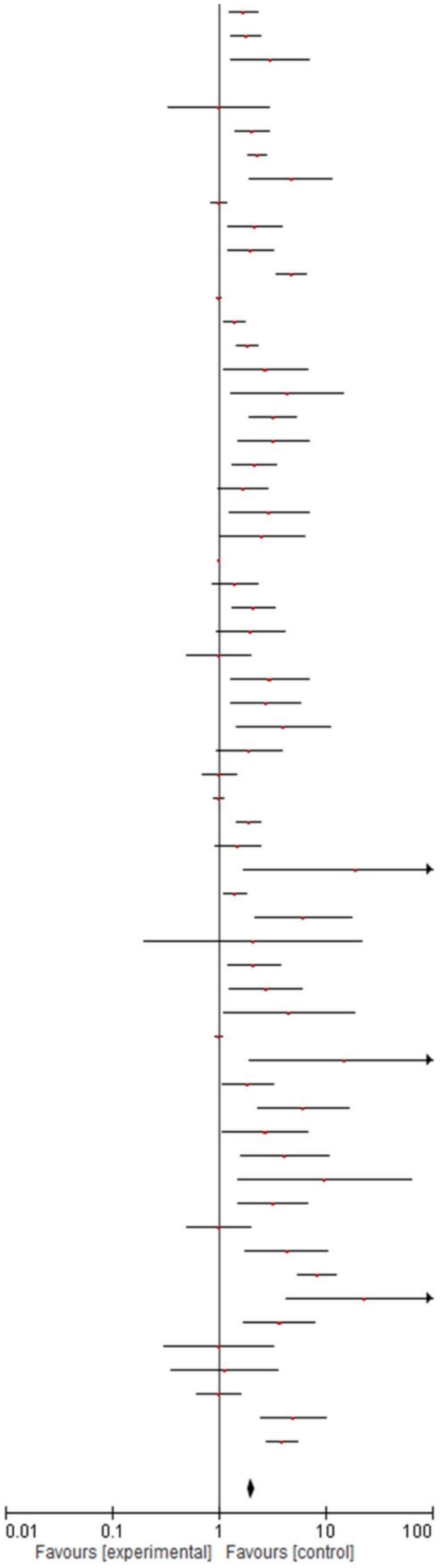

(3B) Funnel plots

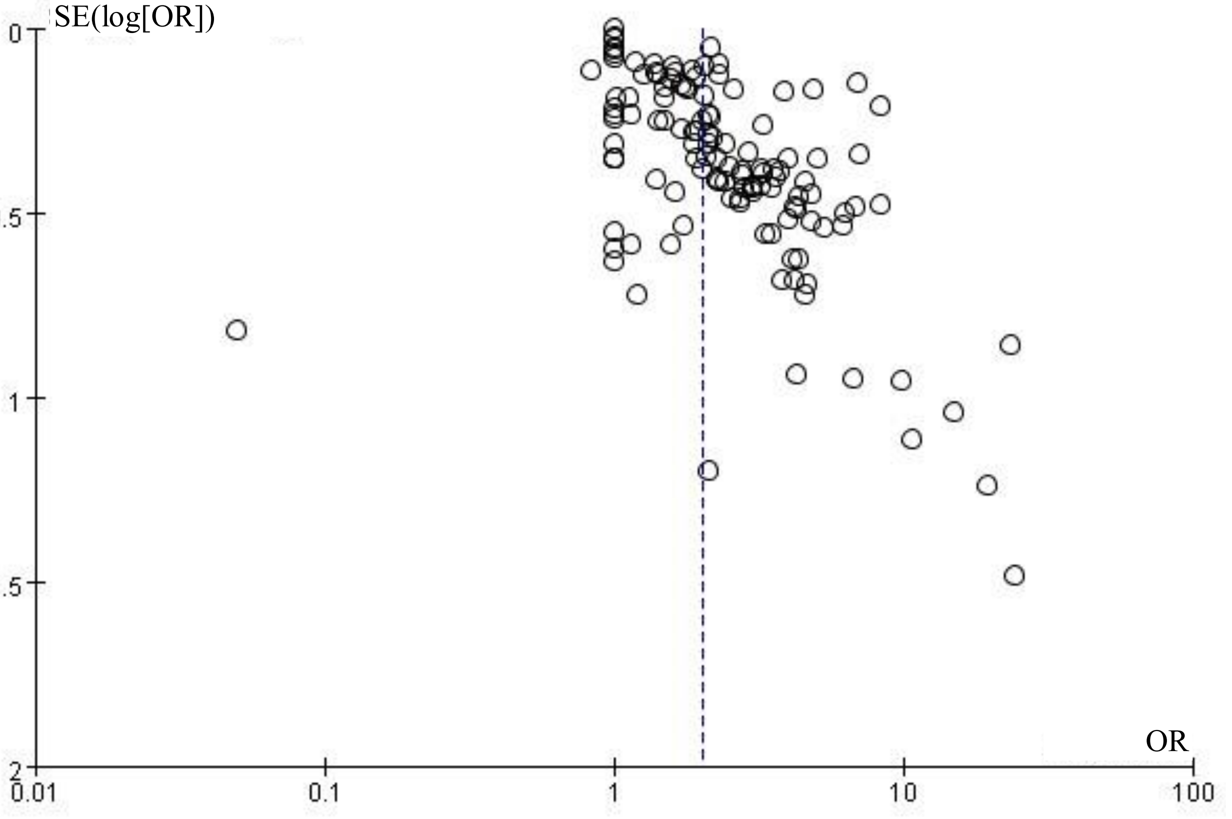

Supplemental Figure 4. Adjusted analyses in subgroup patients: (4A) acquisition places; (4B) bacteraemia severity; (4C) specific comorbidities or conditions; (4D) bacteraemia sources; (4E) Gram-positive cocci; (4F) Enterobacteriaceae; (4G) glucose non-fermentative rods; (4H) antibiotic-resistant microorganisms; (4I) varied timeliness of empirical antimicrobial therapy (EAT); and (4J) varied mortality timeliness

(4A) Acquisition places

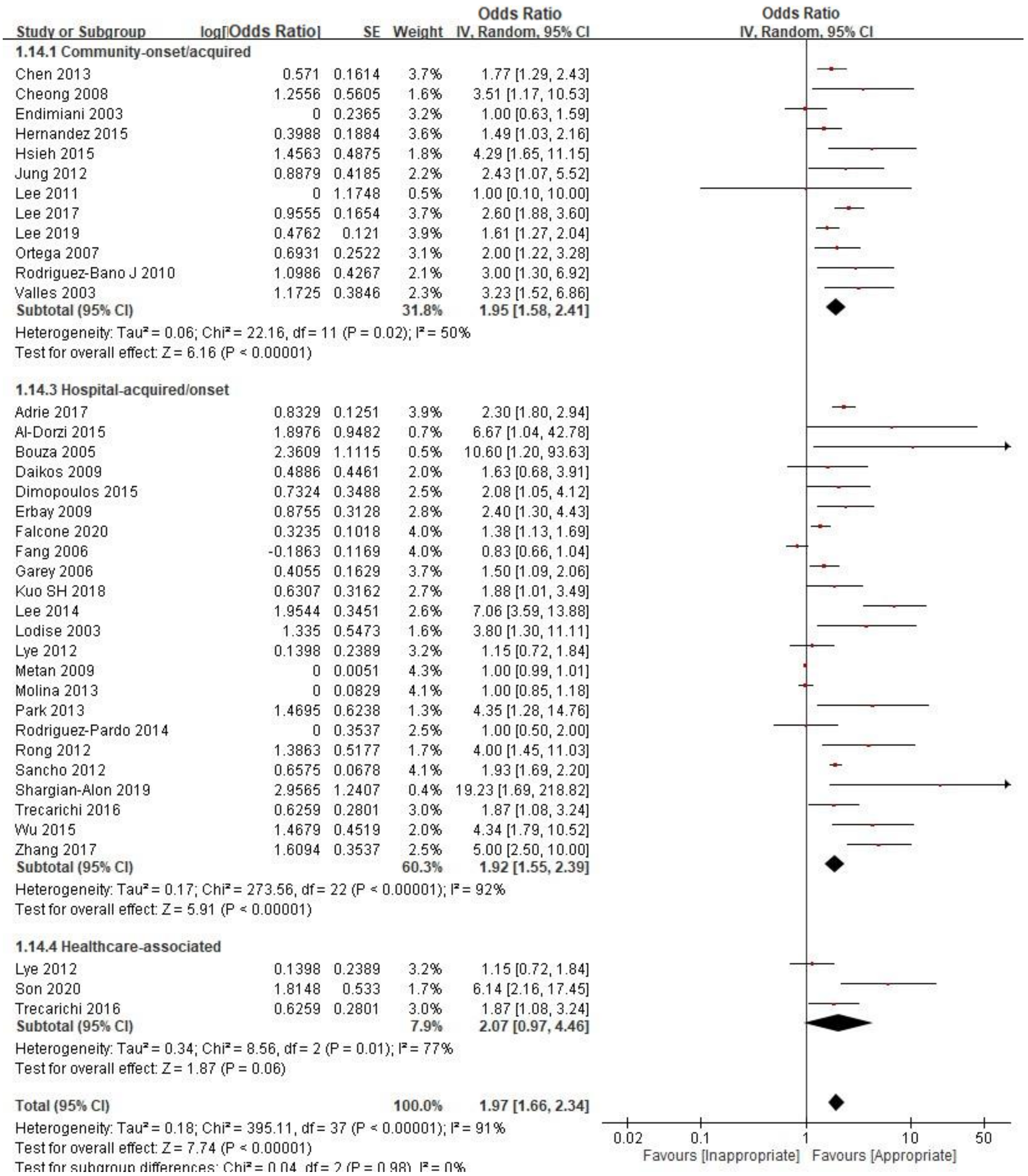

(4B) Bacteraemia severity

Study or Subgroup	log[Odds Ratio]	SE	Weight	Odds Ratio IV, Random, 95% CI
**1.15.1 ICU patients**				
Adrie 2017	0.8329	0.1251	6.9%	2.30 [1.80, 2.94]
Al-Dorzi 2015	1.8976	0.9482	1.7%	6.67 [1.04, 42.78]
Corona 2010	0.1133	0.1918	6.4%	1.12 [0.77, 1.63]
Dimopoulos 2015	0.7324	0.3488	5.1%	2.08 [1.05, 4.12]
Falcone 2020	0.3235	0.1018	7.0%	1.38 [1.13, 1.69]
Ibrahim 2000	1.9257	0.1523	6.7%	6.86 [5.09, 9.25]
Kuo SH 2018	0.6307	0.3162	5.3%	1.88 [1.01, 3.49]
Lee 2014	1.9544	0.3451	5.1%	7.06 [3.59, 13.88]
Sancho 2012	0.6575	0.3563	5.0%	1.93 [0.96, 3.88]
Savage 2016	0.0198	0.1921	6.4%	1.02 [0.70, 1.49]
Shorr 2014	0.3492	0.13	6.8%	1.42 [1.10, 1.83]
Valles 2003	1.1725	0.3846	4.7%	3.23 [1.52, 6.86]
Yamaga & Shime 2017	3.1485	0.8565	2.0%	23.30 [4.35, 124.86]
Zaragoza 2003	0	0.2439	6.0%	1.00 [0.62, 1.61]
**Subtotal (95% CI)**			**75.2%**	**2.26 [1.56, 3.28]**
Heterogeneity: Tau² = 0.39; Chi² = 139.99, df = 13 (P < 0.00001); I² = 91%				
Test for overall effect: Z = 4.31 (P < 0.0001)				
**1.15.3 Severe sepsis and spetic shock**				
Johnson 2011	0.708	0.1027	7.0%	2.03 [1.66, 2.48]
Ziberberg 2014	1.3538	0.1709	6.6%	3.87 [2.77, 5.41]
**Subtotal (95% CI)**			**13.6%**	**2.76 [1.47, 5.20]**
Heterogeneity: Tau² = 0.19; Chi² = 10.49, df = 1 (P = 0.001); I² = 90%				
Test for overall effect: Z = 3.15 (P = 0.002)				
**1.15.4 Pitt bacteraemia score ≥ 4**				
Lee 2013	1.1709	0.4301	4.4%	3.22 [1.39, 7.49]
Lee 2019	0.4781	0.1237	6.9%	1.61 [1.27, 2.06]
**Subtotal (95% CI)**			**11.2%**	**2.02 [1.07, 3.81]**
Heterogeneity: Tau² = 0.14; Chi² = 2.40, df = 1 (P = 0.12); I² = 58%				
Test for overall effect: Z = 2.17 (P = 0.03)				
**Total (95% CI)**			**100.0%**	**2.27 [1.72, 2.99]**
Heterogeneity: Tau² = 0.28; Chi² = 160.31, df = 17 (P < 0.00001); I² = 89%				
Test for overall effect: Z = 5.76 (P < 0.00001)				
Test for subgroup differences: Chi² = 0.49, df = 2 (P = 0.78), I² = 0%				

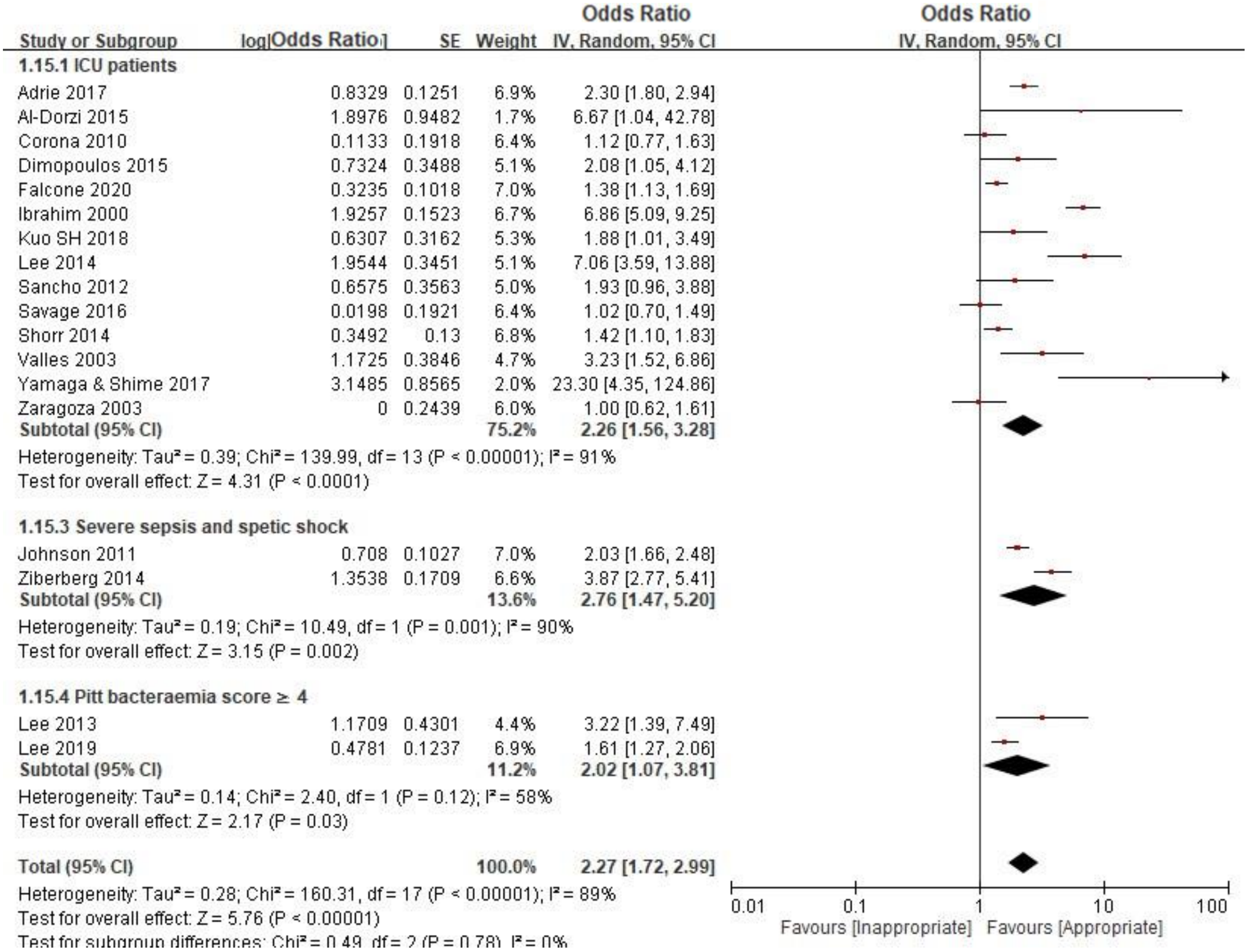

(4C) Specific comorbidities or condition

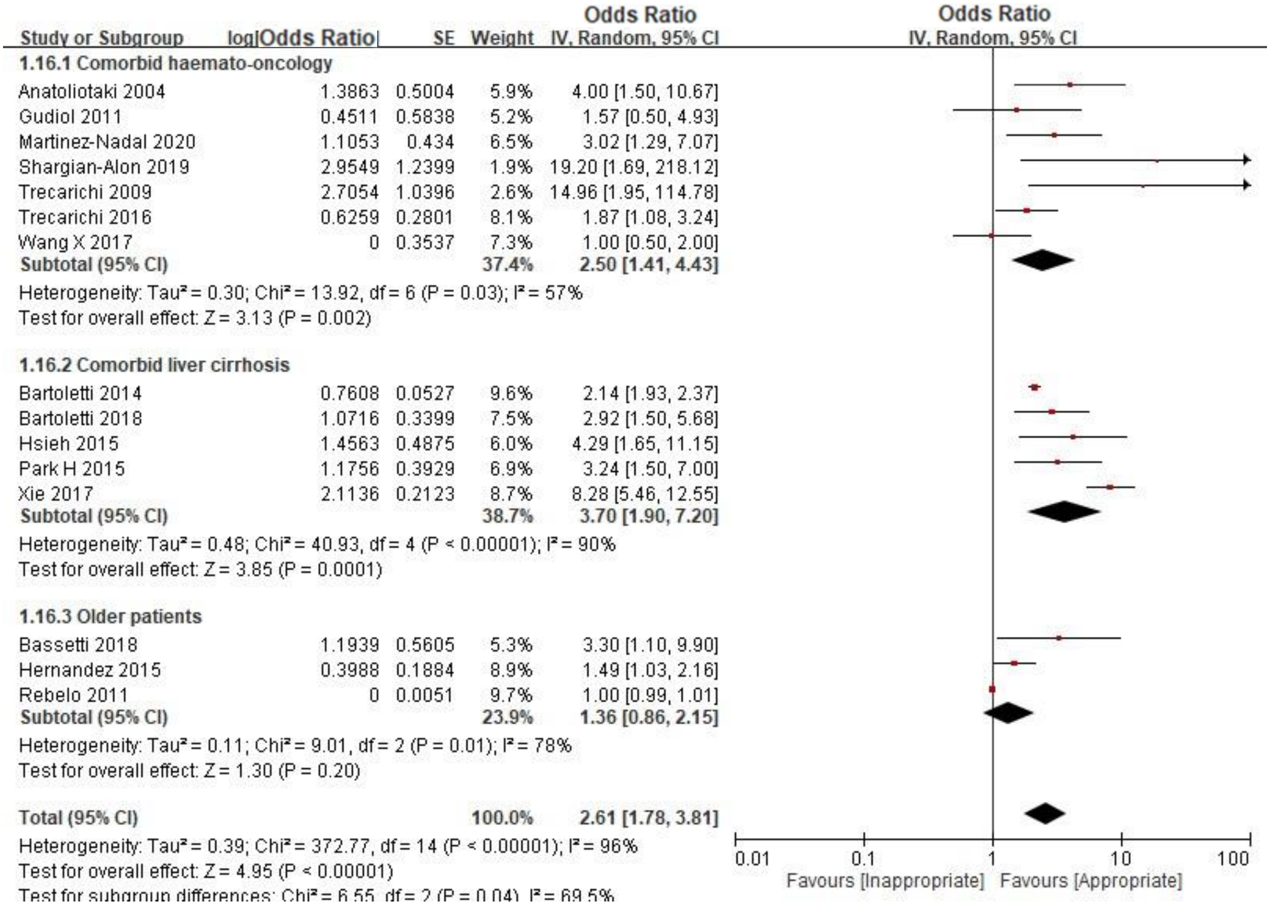

(4D) Bacteraemia source

Study or Subgroup	log[Odds Ratio]	SE	Weight	Odds Ratio IV, Random, 95% CI
**1.17.1 Vascular catheter**				
Garnacho-Montero 2013	0.9163	0.3745	5.8%	2.50 [1.20, 5.21]
Kuo SH 2018	0.6307	0.3162	7.2%	1.88 [1.01, 3.49]
Lin 2010	1.4341	0.6831	2.3%	4.20 [1.10, 16.01]
Rodriguez-Pardo 2014	1.0225	0.3883	5.5%	2.78 [1.30, 5.95]
**Subtotal (95% CI)**			**20.7%**	**2.40 [1.63, 3.53]**
Heterogeneity: Tau² = 0.00; Chi² = 1.42, df = 3 (P = 0.70); I² = 0%				
Test for overall effect: Z = 4.46 (P < 0.00001)				
**1.17.2 Pneumonia**				
Cheng 2016	1.6677	0.5459	3.3%	5.30 [1.82, 15.45]
Guillamet 2016	0.8329	0.1852	11.7%	2.30 [1.60, 3.31]
Park 2012	1.0043	0.4592	4.3%	2.73 [1.11, 6.71]
Park 2018	1.1756	0.2617	8.8%	3.24 [1.94, 5.41]
**Subtotal (95% CI)**			**28.1%**	**2.72 [2.07, 3.57]**
Heterogeneity: Tau² = 0.00; Chi² = 2.76, df = 3 (P = 0.43); I² = 0%				
Test for overall effect: Z = 7.19 (P < 0.00001)				
**1.17.3 Biliary tract**				
Kang 2013	0.8109	0.3514	6.3%	2.25 [1.13, 4.48]
Ortega 2012	0.3365	0.123	14.4%	1.40 [1.10, 1.78]
Tagashira 2017	1.0225	0.3997	5.3%	2.78 [1.27, 6.09]
**Subtotal (95% CI)**			**26.0%**	**1.82 [1.17, 2.83]**
Heterogeneity: Tau² = 0.08; Chi² = 3.95, df = 2 (P = 0.14); I² = 49%				
Test for overall effect: Z = 2.67 (P = 0.007)				
**1.17.5 Urinary tract**				
Elhanan 1997	0	0.2365	9.7%	1.00 [0.63, 1.59]
Lee 2011	0	1.1748	0.8%	1.00 [0.10, 10.00]
Park 2013	0.6206	0.1166	14.7%	1.86 [1.48, 2.34]
**Subtotal (95% CI)**			**25.2%**	**1.40 [0.82, 2.38]**
Heterogeneity: Tau² = 0.12; Chi² = 5.72, df = 2 (P = 0.06); I² = 65%				
Test for overall effect: Z = 1.23 (P = 0.22)				
**Total (95% CI)**			**100.0%**	**2.08 [1.67, 2.58]**
Heterogeneity: Tau² = 0.07; Chi² = 27.11, df = 13 (P = 0.01); I² = 52%				
Test for overall effect: Z = 6.64 (P < 0.00001)				
Test for subgroup differences: Chi² = 5.89, df = 3 (P = 0.12), I² = 49.0%				

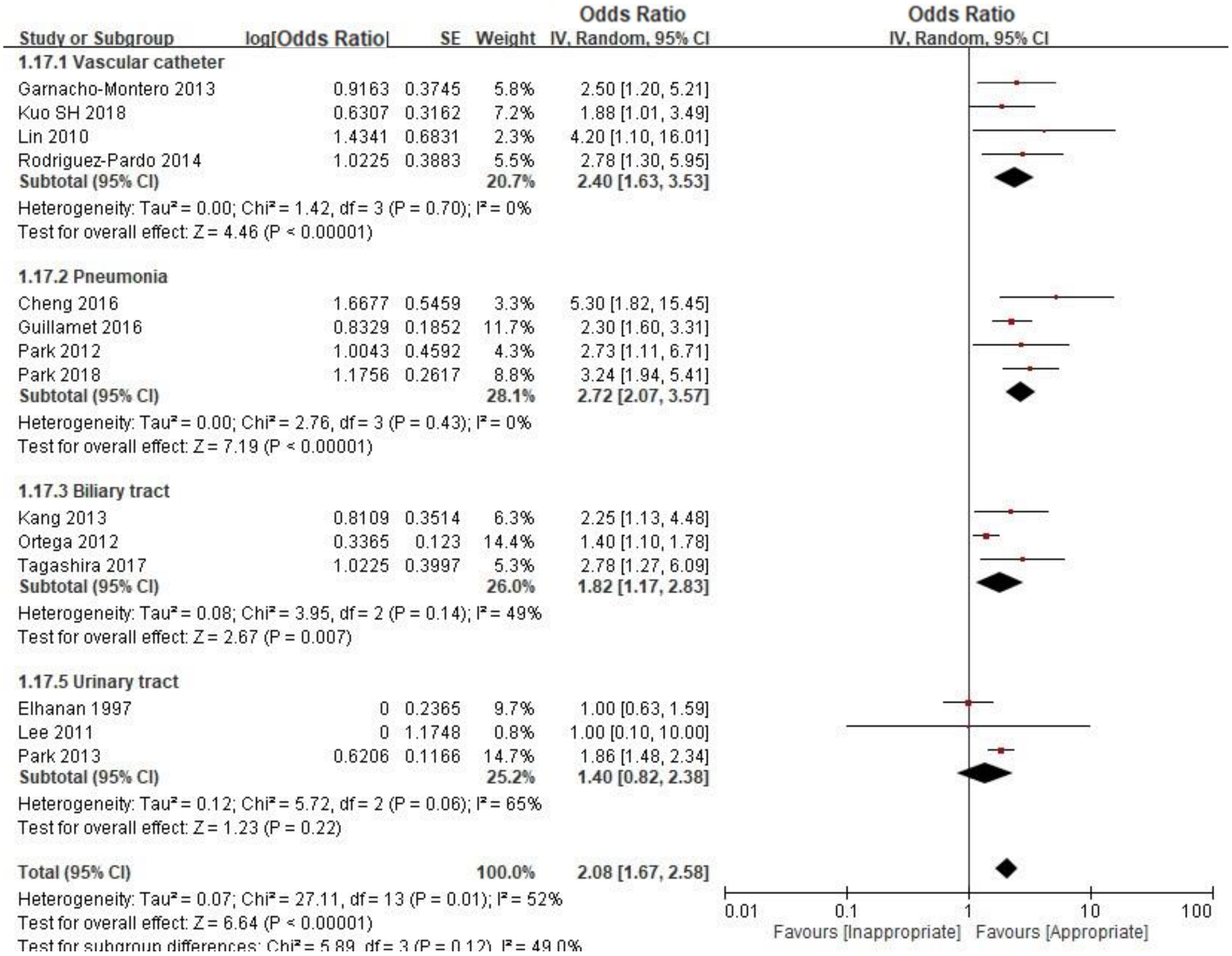

(4E) Gram-positive cocci

Study or Subgroup	log[Odds Ratio]	SE	Weight	Odds Ratio IV, Random, 95% CI
**1.18.1 Staphylococcus aureus**				
Bassetti 2012	1.026	0.4347	5.5%	2.79 [1.19, 6.54]
Bassetti 2018	1.203	0.5605	4.4%	3.33 [1.11, 9.99]
Fang 2006	-0.1863	0.1169	8.6%	0.83 [0.66, 1.04]
Gasch 2013	1.2782	0.4029	5.8%	3.59 [1.63, 7.91]
Guilarde 2006	0.8198	0.4082	5.8%	2.27 [1.02, 5.05]
Khatib 2006	0	0.2198	7.7%	1.00 [0.65, 1.54]
Kim 2006	0.3293	0.4119	5.7%	1.39 [0.62, 3.12]
Lee 2015	1.914	0.4871	5.0%	6.78 [2.61, 17.61]
Lodise 2003	1.335	0.5473	4.5%	3.80 [1.30, 11.11]
Paul 2010	0.7655	0.2412	7.5%	2.15 [1.34, 3.45]
Rong 2012	1.3863	0.5181	4.8%	4.00 [1.45, 11.04]
Schramm 2006	0.6523	0.1328	8.5%	1.92 [1.48, 2.49]
Schweizer 2010	0.4055	0.2277	7.7%	1.50 [0.96, 2.34]
Soriano 2000	0.7561	0.2885	7.0%	2.13 [1.21, 3.75]
Takesue 2011	1.5129	0.7242	3.3%	4.54 [1.10, 18.77]
**Subtotal (95% CI)**			**91.9%**	**2.12 [1.55, 2.92]**
Heterogeneity: Tau² = 0.26; Chi² = 64.40, df = 14 (P < 0.00001); I² = 78%				
Test for overall effect: Z = 4.65 (P < 0.00001)				
**1.18.2 Entercoccus spp.**				
Zhang 2017	1.6094	0.3537	6.3%	5.00 [2.50, 10.00]
**Subtotal (95% CI)**			**6.3%**	**5.00 [2.50, 10.00]**
Heterogeneity: Not applicable				
Test for overall effect: Z = 4.55 (P < 0.00001)				
**1.18.3 Streptococcus spp.**				
Bouza 2005	2.3609	1.1115	1.8%	10.60 [1.20, 93.63]
**Subtotal (95% CI)**			**1.8%**	**10.60 [1.20, 93.63]**
Heterogeneity: Not applicable				
Test for overall effect: Z = 2.12 (P = 0.03)				
**Total (95% CI)**			**100.0%**	**2.33 [1.69, 3.22]**
Heterogeneity: Tau² = 0.30; Chi² = 78.47, df = 16 (P < 0.00001); I² = 80%				
Test for overall effect: Z = 5.15 (P < 0.00001)				
Test for subgroup differences: Chi² = 6.54, df = 2 (P = 0.04), I² = 69.4%				

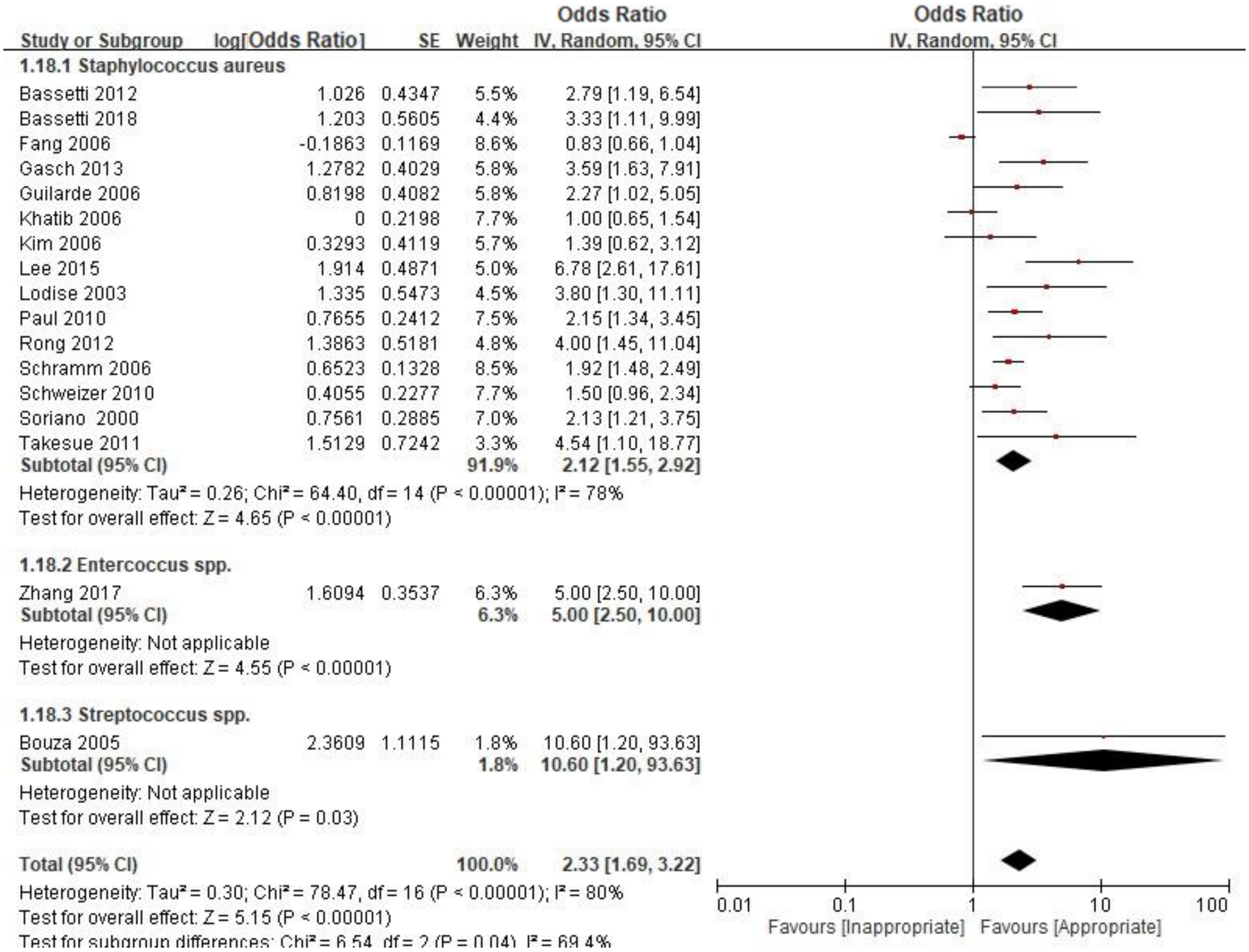

(4F) Enterobacteriaceae

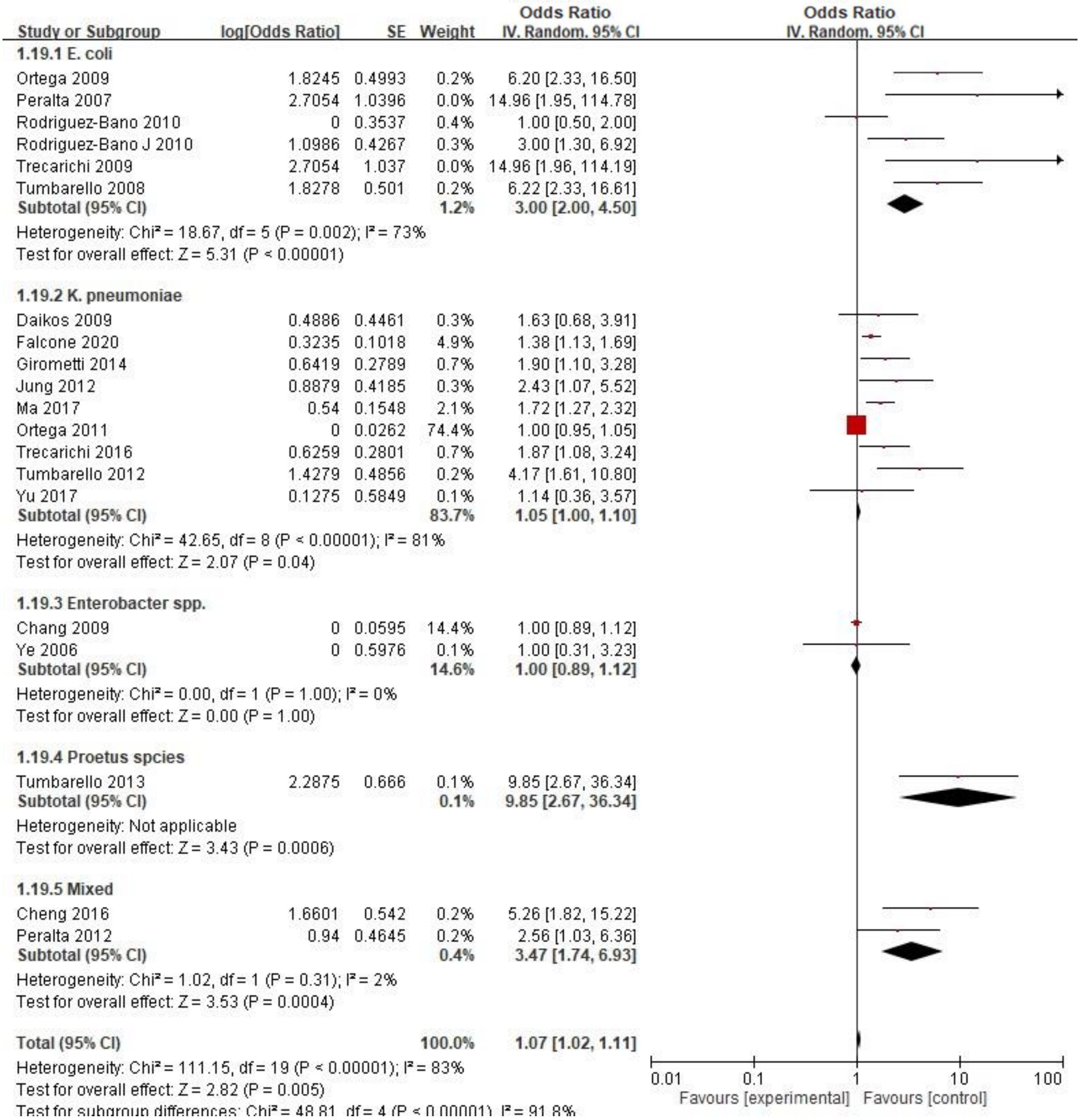

(4G) Glucose non-fermentative rods

Study or Subgroup	log[Odds Ratio]	SE	Weight	Odds Ratio IV, Random, 95% CI
**1.20.1 Pseudomonas spp.**				
Chamot 2003	0.1823	0.7246	0.1%	1.20 [0.29, 4.97]
Kang 2003	1.5282	0.6953	0.1%	4.61 [1.18, 18.01]
Lodise 2007	1.411	0.6269	0.1%	4.10 [1.20, 14.01]
Martinez-Nadal 2020	1.1053	0.434	0.3%	3.02 [1.29, 7.07]
Micek & Lioyd 2010	0.7129	0.1848	1.7%	2.04 [1.42, 2.93]
Migiyama 2016	1.5602	0.4527	0.3%	4.76 [1.96, 11.56]
Morata 2012	0.7793	0.2983	0.6%	2.18 [1.21, 3.91]
Park 2012	1.0043	0.4592	0.3%	2.73 [1.11, 6.71]
Pena 2013	0.5306	0.2759	0.8%	1.70 [0.99, 2.92]
Schechner 2011	0	0.0538	19.8%	1.00 [0.90, 1.11]
Tam 2010	0	0.0316	57.4%	1.00 [0.94, 1.06]
Tumbarello 2011	1.0043	0.4731	0.3%	2.73 [1.08, 6.90]
**Subtotal (95% CI)**			**81.7%**	**1.05 [0.99, 1.10]**
Heterogeneity: Chi² = 59.92, df = 11 (P < 0.00001); I² = 82%				
Test for overall effect: Z = 1.76 (P = 0.08)				
**1.20.2 Acinetobacter spp**				
Al-Dorzi 2015	1.8976	0.9482	0.1%	6.67 [1.04, 42.78]
Chusri 2019	-2.9957	0.8212	0.1%	0.05 [0.01, 0.25]
Erbay 2009	0.8755	0.3128	0.6%	2.40 [1.30, 4.43]
Falagas 2006	0	0.0711	11.3%	1.00 [0.87, 1.15]
Lee 2013	1.1709	0.4301	0.3%	3.22 [1.39, 7.49]
Lee 2014	1.9544	0.3451	0.5%	7.06 [3.59, 13.88]
Lee NY 2012	1.5129	0.4183	0.3%	4.54 [2.00, 10.31]
Metan 2009	0	0.5504	0.2%	1.00 [0.34, 2.94]
Park 2018	1.1756	0.2617	0.8%	3.24 [1.94, 5.41]
Shargian-Alon 2019	2.9549	1.2384	0.0%	19.20 [1.69, 217.48]
Shorr 2014	0.3492	0.13	3.4%	1.42 [1.10, 1.83]
Song 2011	0.7419	1.1997	0.0%	2.10 [0.20, 22.05]
Wang X 2017	0	0.3537	0.5%	1.00 [0.50, 2.00]
**Subtotal (95% CI)**			**18.1%**	**1.28 [1.15, 1.43]**
Heterogeneity: Chi² = 91.83, df = 12 (P < 0.00001); I² = 87%				
Test for overall effect: Z = 4.40 (P < 0.0001)				
**1.20.3 Burkholderia spp.**				
Ku 2011	3.1748	1.4809	0.0%	23.92 [1.31, 435.86]
**Subtotal (95% CI)**			**0.0%**	**23.92 [1.31, 435.86]**
Heterogeneity: Not applicable				
Test for overall effect: Z = 2.14 (P = 0.03)				
**1.20.4 Mixed**				
Park 2013	1.4695	0.6238	0.1%	4.35 [1.28, 14.76]
**Subtotal (95% CI)**			**0.1%**	**4.35 [1.28, 14.76]**
Heterogeneity: Not applicable				
Test for overall effect: Z = 2.36 (P = 0.02)				
**Total (95% CI)**			**100.0%**	**1.09 [1.04, 1.14]**
Heterogeneity: Chi² = 171.47, df = 26 (P < 0.00001); I² = 85%				
Test for overall effect: Z = 3.59 (P = 0.0003)				
Test for subgroup differences: Chi² = 19.72, df = 3 (P = 0.0002), I² = 84.8%				

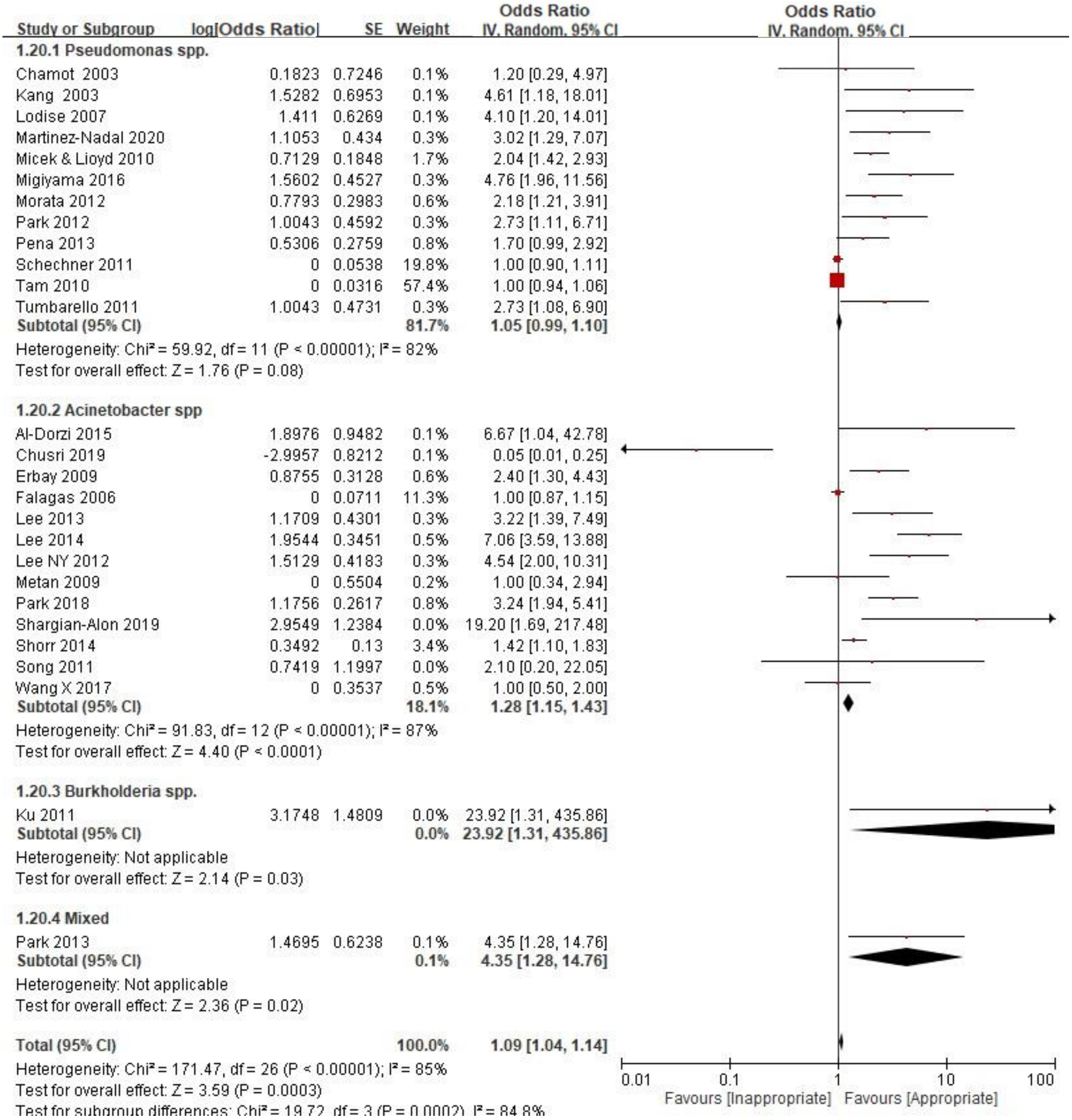

(4H) Antibiotic-resistant microorganism

Study or Subgroup	log[Odds Ratio]	SE	Weight	Odds Ratio IV, Random, 95% CI
**1.21.1 MRSA**				
Fang 2006	-0.1863	0.1169	9.5%	0.83 [0.66, 1.04]
Gasch 2013	1.2782	0.4029	6.2%	3.59 [1.63, 7.91]
Lee 2015	1.914	0.4871	5.3%	6.78 [2.61, 17.61]
Paul 2010	0.7655	0.2412	8.2%	2.15 [1.34, 3.45]
Schramm 2006	0.6523	0.1328	9.4%	1.92 [1.48, 2.49]
Takesue 2011	1.5129	0.7238	3.3%	4.54 [1.10, 18.76]
**Subtotal (95% CI)**			**41.9%**	**2.34 [1.30, 4.21]**
Heterogeneity: Tau² = 0.41; Chi² = 47.39, df = 5 (P < 0.00001); I² = 89%				
Test for overall effect: Z = 2.85 (P = 0.004)				
**1.21.2 ESBL-producing Enterobactericeae**				
Cheng 2016	1.6601	0.542	4.7%	5.26 [1.82, 15.22]
Kang 2005	0	0.3144	7.3%	1.00 [0.54, 1.85]
Peralta 2012	0.9416	0.4649	5.5%	2.56 [1.03, 6.38]
Rodriguez-Bano 2010	0	0.3537	6.8%	1.00 [0.50, 2.00]
Tumbarello 2008	1.8278	0.501	5.1%	6.22 [2.33, 16.61]
Yu 2017	0.1275	0.5863	4.3%	1.14 [0.36, 3.58]
**Subtotal (95% CI)**			**33.7%**	**2.03 [1.05, 3.93]**
Heterogeneity: Tau² = 0.47; Chi² = 17.40, df = 5 (P = 0.004); I² = 71%				
Test for overall effect: Z = 2.10 (P = 0.04)				
**1.21.4 Carbapenem-resistant Enterobactericeae**				
Falcone 2020	0.3235	0.1018	9.6%	1.38 [1.13, 1.69]
Park 2018	1.1756	0.2617	7.9%	3.24 [1.94, 5.41]
Song 2011	0.7419	1.1997	1.5%	2.10 [0.20, 22.05]
Tumbarello 2012	1.4279	0.4856	5.3%	4.17 [1.61, 10.80]
**Subtotal (95% CI)**			**24.4%**	**2.40 [1.21, 4.74]**
Heterogeneity: Tau² = 0.30; Chi² = 13.28, df = 3 (P = 0.004); I² = 77%				
Test for overall effect: Z = 2.52 (P = 0.01)				
**Total (95% CI)**			**100.0%**	**2.17 [1.58, 2.99]**
Heterogeneity: Tau² = 0.26; Chi² = 79.68, df = 15 (P < 0.00001); I² = 81%				
Test for overall effect: Z = 4.80 (P < 0.00001)				
Test for subgroup differences: Chi² = 0.15, df = 2 (P = 0.93), I² = 0%				

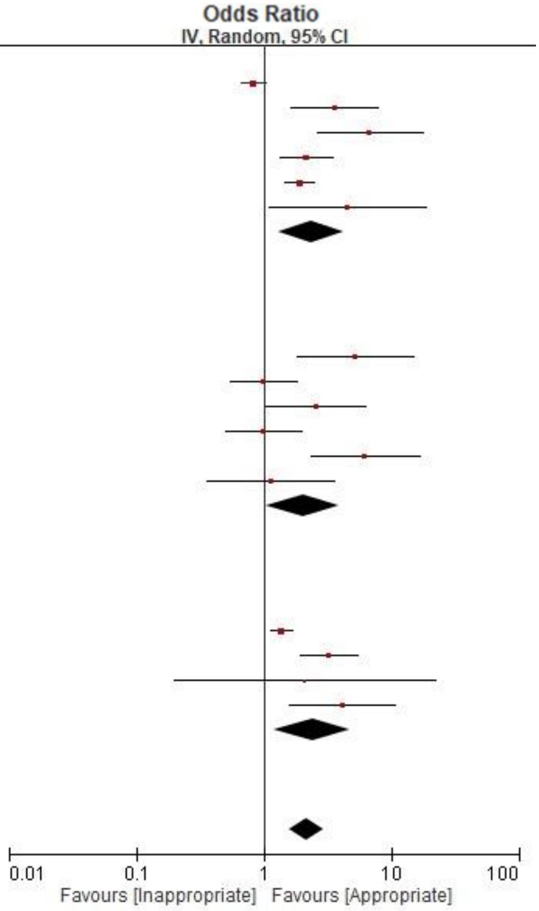

(4I) Varied EAT timeliness

Study or Subgroup	log[Odds Ratio]	SE	Weight	Odds Ratio IV, Random, 95% CI
**1.22.1 0 hour**				
Corona 2010	0.1133	0.1918	1.1%	1.12 [0.77, 1.63]
Elhanan 1997	0	0.2365	1.0%	1.00 [0.63, 1.59]
Guillamet 2016	0.8329	0.4149	0.6%	2.30 [1.02, 5.19]
Hanon 2002	0.4447	0.1381	1.3%	1.56 [1.19, 2.04]
Khatib 2006	0	0.6143	0.4%	1.00 [0.30, 3.33]
Lee 2013	1.1709	0.4298	0.6%	3.22 [1.39, 7.49]
Lee CC 2012	0.8154	0.4109	0.6%	2.26 [1.01, 5.06]
Martinez-Nadal 2020	1.1053	0.434	0.6%	3.02 [1.29, 7.07]
Micek & Lloyd 2010	0.7129	0.1848	1.1%	2.04 [1.42, 2.93]
Molina 2013	0	0.0829	1.4%	1.00 [0.85, 1.18]
Park H 2015	1.1756	0.3929	0.7%	3.24 [1.50, 7.00]
Trecarichi 2016	0.6259	0.2801	0.9%	1.87 [1.08, 3.24]
Tumbarello 2012	1.4279	0.4856	0.5%	4.17 [1.61, 10.80]
Yu 2017	7.0353	4.1093	0.0%	1136.04 [0.36, 3574657.77]
**Subtotal (95% CI)**			**10.8%**	**1.76 [1.35, 2.30]**

Heterogeneity: Tau² = 0.14; Chi² = 47.07, df = 13 (P < 0.00001); I² = 72%
Test for overall effect: Z = 4.16 (P < 0.0001)

Study or Subgroup	log[Odds Ratio]	SE	Weight	Odds Ratio IV, Random, 95% CI
**1.22.2 24 hours**				
Adrie 2017	0.8329	0.1251	1.3%	2.30 [1.80, 2.94]
Anatoliotaki 2004	1.3863	0.3537	0.7%	4.00 [2.00, 8.00]
Bartoletti 2014	1.0716	0.3399	0.8%	2.92 [1.50, 5.68]
Bartoletti 2018	0.7608	0.0527	1.4%	2.14 [1.93, 2.37]
Boel 2015	0.239	0.1271	1.3%	1.27 [0.99, 1.63]
Bouza 2004	1.2528	0.4323	0.6%	3.50 [1.50, 8.17]
Bouza 2005	2.3609	1.1115	0.1%	10.60 [1.20, 93.63]
Byl 1999	0.7561	0.3149	0.8%	2.13 [1.15, 3.95]
Chamot 2003	0.1823	0.7246	0.3%	1.20 [0.29, 4.97]
Chen 2013	0.571	0.1614	1.2%	1.77 [1.29, 2.43]
Cheong 2008	1.2556	0.5605	0.4%	3.51 [1.17, 10.53]
Falcone 2020	0.3235	0.1018	1.3%	1.38 [1.13, 1.69]
Garey 2006	0.4055	0.1629	1.2%	1.50 [1.09, 2.06]
Hernandez 2015	0.3988	0.1884	1.1%	1.49 [1.03, 2.16]
Johnson 2011	0.708	0.1027	1.3%	2.03 [1.66, 2.48]
Kang 2003	1.5282	0.6953	0.3%	4.61 [1.18, 18.01]
Kang 2005	0	0.3144	0.8%	1.00 [0.54, 1.85]
Kang 2013	0.8109	0.3514	0.7%	2.25 [1.13, 4.48]
Khan 2010	2.1223	0.4799	0.5%	8.35 [3.26, 21.39]
Ku 2011	3.1747	1.4808	0.1%	23.92 [1.31, 435.73]
Labelle AJ 2012	0	0.6316	0.4%	1.00 [0.29, 3.45]
Lee 2019	0.4824	0.1258	1.3%	1.62 [1.27, 2.07]
Lye 2012	0.1389	0.2356	1.0%	1.15 [0.72, 1.82]
Ma 2017	0.54	0.1548	1.2%	1.72 [1.27, 2.32]
Metan 2009	0	0.5504	0.4%	1.00 [0.34, 2.94]
Micek 2010	0.8329	0.1002	1.3%	2.30 [1.89, 2.80]
Morata 2012	0.7793	0.2983	0.9%	2.18 [1.21, 3.91]
Pena 2013	0.5306	0.2759	0.9%	1.70 [0.99, 2.92]
Peralta 2007	1.0919	0.4433	0.6%	2.98 [1.25, 7.10]
Peralta 2012	0.94	0.4645	0.5%	2.56 [1.03, 6.36]
Retamar 2013	0.6931	0.3798	0.7%	2.00 [0.95, 4.21]
Rodriguez-Bano 2010	0	0.3537	0.7%	1.00 [0.50, 2.00]
Rodriguez-Bano J 2010	1.0986	0.4267	0.6%	3.00 [1.30, 6.92]
Savage 2016	0.0198	0.1921	1.1%	1.02 [0.70, 1.49]
Schechner 2011	0	0.0538	1.4%	1.00 [0.90, 1.11]
Schramm 2006	0.6523	0.1328	1.3%	1.92 [1.48, 2.49]
Schweizer 2010	0.4055	0.2494	1.0%	1.50 [0.92, 2.45]
Shorr 2014	0.3492	0.13	1.3%	1.42 [1.10, 1.83]
Son 2020	1.8148	0.533	0.4%	6.14 [2.16, 17.45]
Tam 2010	0	0.0316	1.4%	1.00 [0.94, 1.06]
Valles 2003	1.1725	0.3846	0.7%	3.23 [1.52, 6.86]
Xie 2017	2.1136	0.2123	1.1%	8.28 [5.46, 12.55]
Yamaga & Shime 2017	3.1495	0.8572	0.2%	23.32 [4.35, 125.16]
Yang 2013	1.3124	0.3882	0.7%	3.72 [1.74, 7.95]
Zhang 2017	1.6094	0.3537	0.7%	5.00 [2.50, 10.00]
Ziberberg 2014	1.3538	0.1709	1.2%	3.87 [2.77, 5.41]
**Subtotal (95% CI)**			**39.5%**	**2.08 [1.77, 2.44]**

Heterogeneity: Tau² = 0.20; Chi² = 496.04, df = 45 (P < 0.00001); I² = 91%
Test for overall effect: Z = 9.03 (P < 0.00001)

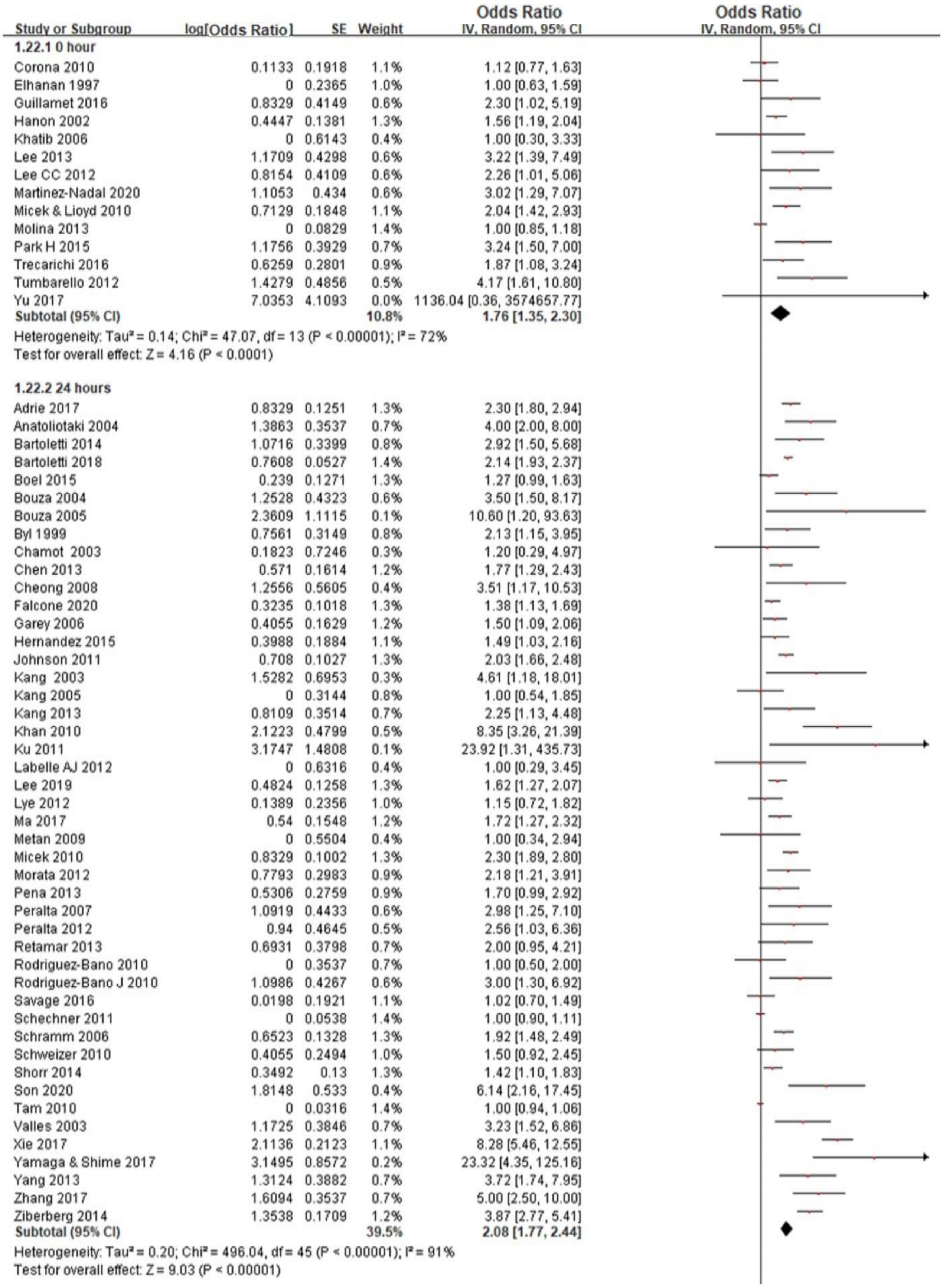

(4I) Varied EAT timeliness (continued)

**1.22.3 48 hours**				
Cain 2015	1.2641	0.3833	0.7%	3.54 [1.67, 7.50]
Daikos 2009	0.4886	0.4461	0.6%	1.63 [0.68, 3.91]
Erbay 2009	0.8755	0.3128	0.8%	2.40 [1.30, 4.43]
Evans 2009	0.5539	0.5348	0.4%	1.74 [0.61, 4.96]
Fang 2006	-0.1863	0.1169	1.3%	0.83 [0.66, 1.04]
Garnacho-Montero 2013	0.9163	0.3745	0.7%	2.50 [1.20, 5.21]
Gasch 2013	1.2782	0.4029	0.6%	3.59 [1.63, 7.91]
Guilarde 2006	0.8198	0.4082	0.6%	2.27 [1.02, 5.05]
Kim 2006	0.3293	0.4119	0.6%	1.39 [0.62, 3.12]
Kuo SH 2018	0.6307	0.3162	0.8%	1.88 [1.01, 3.49]
Labelle AJ 2012	0	0.6316	0.4%	1.00 [0.29, 3.45]
Lee 2014	1.9459	0.3407	0.8%	7.00 [3.59, 13.65]
Lee 2015	1.914	0.4871	0.5%	6.78 [2.61, 17.61]
Lee 2017	0.9555	0.1654	1.2%	2.60 [1.88, 3.60]
Lee 2019	0.6152	0.1393	1.3%	1.85 [1.41, 2.43]
Lee NY 2012	1.5129	0.4183	0.6%	4.54 [2.00, 10.31]
Leibovici 1998	0.47	0.1059	1.3%	1.60 [1.30, 1.97]
Lodise 2003	1.335	0.5473	0.4%	3.80 [1.30, 11.11]
Lodise 2007	1.411	0.6269	0.4%	4.10 [1.20, 14.01]
Migiyama 2016	1.5602	0.4527	0.6%	4.76 [1.96, 11.56]
Park 2012	1.0043	0.4592	0.5%	2.73 [1.11, 6.71]
Paul 2010	0.7655	0.2412	1.0%	2.15 [1.34, 3.45]
Reisfeld 2011	0.3436	0.2523	1.0%	1.41 [0.86, 2.31]
Rodriguez-Pardo 2014	1.0188	0.3868	0.7%	2.77 [1.30, 5.91]
Rong 2012	1.3863	0.5181	0.5%	4.00 [1.45, 11.04]
Shargian-Alon 2019	2.9565	1.2407	0.1%	19.23 [1.69, 218.82]
Song 2011	0.7419	1.1997	0.1%	2.10 [0.20, 22.05]
Trecarichi 2009	2.7054	1.0396	0.2%	14.96 [1.95, 114.78]
Wang X 2017	0	0.3537	0.7%	1.00 [0.50, 2.00]
Wu 2015	1.4697	0.4542	0.6%	4.35 [1.79, 10.59]
**Subtotal (95% CI)**			**19.9%**	**2.45 [1.95, 3.08]**
Heterogeneity: Tau² = 0.24; Chi² = 116.03, df = 29 (P < 0.00001); I² = 75%				
Test for overall effect: Z = 7.64 (P < 0.00001)				
**1.22.4 72 hours**				
Bassetti 2007	1.5581	0.5213	0.5%	4.75 [1.71, 13.19]
Falagas 2006	0	0.0711	1.4%	1.00 [0.87, 1.15]
Hsieh 2015	1.4563	0.4875	0.5%	4.29 [1.65, 11.15]
Lin 2010	1.4341	0.6831	0.3%	4.20 [1.10, 16.01]
Molina 2013	0	0.0829	1.4%	1.00 [0.85, 1.18]
Park 2013	1.4695	0.6238	0.4%	4.35 [1.28, 14.76]
**Subtotal (95% CI)**			**4.4%**	**1.70 [1.15, 2.51]**
Heterogeneity: Tau² = 0.12; Chi² = 26.83, df = 5 (P < 0.0001); I² = 81%				
Test for overall effect: Z = 2.68 (P = 0.007)				
**1.22.5 5 days**				
Cheng 2016	1.6601	0.542	0.4%	5.26 [1.82, 15.22]
Park 2018	1.1756	0.2617	0.9%	3.24 [1.94, 5.41]
Ye 2006	0	0.5976	0.4%	1.00 [0.31, 3.23]
**Subtotal (95% CI)**			**1.8%**	**2.76 [1.27, 5.99]**
Heterogeneity: Tau² = 0.26; Chi² = 4.54, df = 2 (P = 0.10); I² = 56%				
Test for overall effect: Z = 2.57 (P = 0.01)				
**1.22.6 Before BC**				
Al-Dorzi 2015	1.8976	0.9482	0.2%	6.67 [1.04, 42.78]
Bassetti 2012	1.026	0.4347	0.6%	2.79 [1.19, 6.54]
Bassetti 2018	1.1939	0.5559	0.4%	3.30 [1.11, 9.81]
Chang 2009	0	0.0595	1.4%	1.00 [0.89, 1.12]
Chusri 2019	-2.9957	0.8212	0.2%	0.05 [0.01, 0.25]
Dimopoulos 2015	0.7324	0.3488	0.7%	2.08 [1.05, 4.12]
Endimiani 2003	1.454	0.9386	0.2%	4.28 [0.68, 26.94]
Girometti 2014	0.6419	0.2789	0.9%	1.90 [1.10, 3.28]
Gradel 2017	0.1621	0.093	1.4%	1.18 [0.98, 1.41]
Gudiol 2011	0.4511	0.5838	0.4%	1.57 [0.50, 4.93]
Ibrahim 2000	1.9257	0.1523	1.2%	6.86 [5.09, 9.25]
Jung 2012	0.8879	0.4185	0.6%	2.43 [1.07, 5.52]
Lee 2011	0	0.3537	0.7%	1.00 [0.50, 2.00]
Martinez 2010	0.5878	0.166	1.2%	1.80 [1.30, 2.49]
Ortega 2007	0.6931	0.2522	1.0%	2.00 [1.22, 3.28]
Ortega 2009	1.5748	0.1673	1.2%	4.83 [3.48, 6.70]
Ortega 2011	0	0.0262	1.4%	1.00 [0.95, 1.05]
Ortega 2012	0.3365	0.123	1.3%	1.40 [1.10, 1.78]
Ortega 2013	0.6206	0.1166	1.3%	1.86 [1.48, 2.34]
Rebelo 2011	0	0.0051	1.5%	1.00 [0.99, 1.01]
Retamar 2012	0.7514	0.2341	1.0%	2.12 [1.34, 3.35]
Sancho 2012	0.6575	0.3563	0.7%	1.93 [0.96, 3.88]
Soriano 2000	0.7561	0.2885	0.9%	2.13 [1.21, 3.75]
Tagashira 2017	1.0225	0.3997	0.6%	2.78 [1.27, 6.09]
Takesue 2011	1.514	0.7248	0.3%	4.54 [1.10, 18.81]
Tumbarello 2008	1.8278	0.501	0.5%	6.22 [2.33, 16.61]
Tumbarello 2011	1.0043	0.4731	0.5%	2.73 [1.08, 6.90]
Tumbarello 2013	2.2875	0.666	0.3%	9.85 [2.67, 36.34]
Zaragoza 2003	0	0.2439	1.0%	1.00 [0.62, 1.61]
**Subtotal (95% CI)**			**23.7%**	**1.85 [1.59, 2.16]**
Heterogeneity: Tau² = 0.09; Chi² = 410.35, df = 28 (P < 0.00001); I² = 93%				
Test for overall effect: Z = 7.91 (P < 0.00001)				
**Total (95% CI)**			**100.0%**	**2.02 [1.85, 2.19]**
Heterogeneity: Tau² = 0.13; Chi² = 1552.04, df = 127 (P < 0.00001); I² = 92%				
Test for overall effect: Z = 16.34 (P < 0.00001)				
Test for subgroup differences: Chi² = 6.41, df = 5 (P = 0.27), I² = 22.0%				

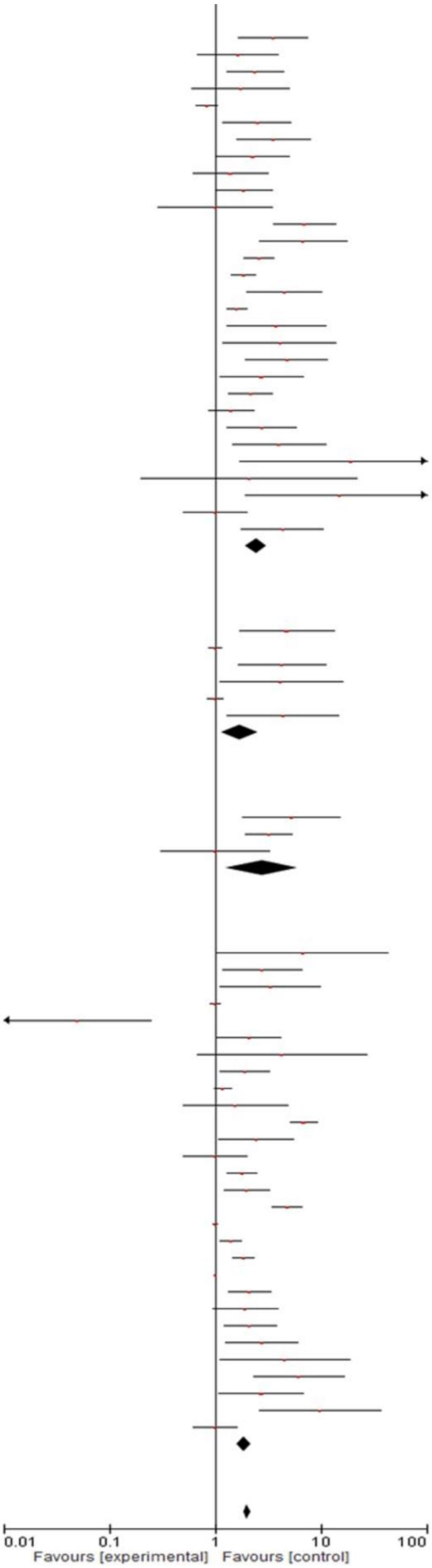

(4J) Varied morality deadline

Study or Subgroup	log[Odds Ratio]	SE	Weight	Odds Ratio IV, Random, 95% CI
**1.23.1 7 days**				
Bassetti 2018	1.1939	0.5559	0.4%	3.30 [1.11, 9.81]
Gasch 2013	1.2782	0.4029	0.6%	3.59 [1.63, 7.91]
Shargian-Alon 2019	2.9565	1.2407	0.1%	19.23 [1.69, 218.82]
Soriano 2000	0.7561	0.2885	0.8%	2.13 [1.21, 3.75]
Zhang 2017	1.6094	0.6422	0.3%	5.00 [1.42, 17.60]
**Subtotal (95% CI)**			**2.3%**	**3.08 [1.98, 4.79]**
Heterogeneity: Tau² = 0.03; Chi² = 4.51, df = 4 (P = 0.34); I² = 11%				
Test for overall effect: Z = 5.00 (P < 0.00001)				
**1.23.2 14 days**				
Daikos 2009	0.4886	0.4461	0.5%	1.63 [0.68, 3.91]
Falagas 2006	0	0.5626	0.4%	1.00 [0.33, 3.01]
Kuo SH 2018	0.6307	0.3162	0.8%	1.88 [1.01, 3.49]
Lee NY 2012	1.5129	0.4183	0.6%	4.54 [2.00, 10.31]
Lin 2010	1.4341	0.6831	0.3%	4.20 [1.10, 16.01]
Metan 2009	0	0.5504	0.4%	1.00 [0.34, 2.94]
Park 2013	1.4695	0.6238	0.3%	4.35 [1.28, 14.76]
Retamar 2012	0.7514	0.2341	1.0%	2.12 [1.34, 3.35]
Retamar 2013	1.203	0.4349	0.6%	3.33 [1.42, 7.81]
Rodriguez-Bano J 2010	1.0986	0.4267	0.6%	3.00 [1.30, 6.92]
**Subtotal (95% CI)**			**5.4%**	**2.31 [1.72, 3.09]**
Heterogeneity: Tau² = 0.04; Chi² = 11.18, df = 9 (P = 0.26); I² = 19%				
Test for overall effect: Z = 5.60 (P < 0.00001)				
**1.23.3 21 days**				
Bassetti 2007	1.5581	0.5213	0.4%	4.75 [1.71, 13.19]
Trecarichi 2016	0.6259	0.2801	0.9%	1.87 [1.08, 3.24]
Tumbarello 2008	1.8278	0.501	0.5%	6.22 [2.33, 16.61]
Tumbarello 2011	1.0043	0.4731	0.5%	2.73 [1.08, 6.90]
Tumbarello 2013	2.2875	0.666	0.3%	9.85 [2.67, 36.34]
**Subtotal (95% CI)**			**2.6%**	**3.78 [2.06, 6.96]**
Heterogeneity: Tau² = 0.26; Chi² = 8.94, df = 4 (P = 0.06); I² = 55%				
Test for overall effect: Z = 4.27 (P < 0.0001)				

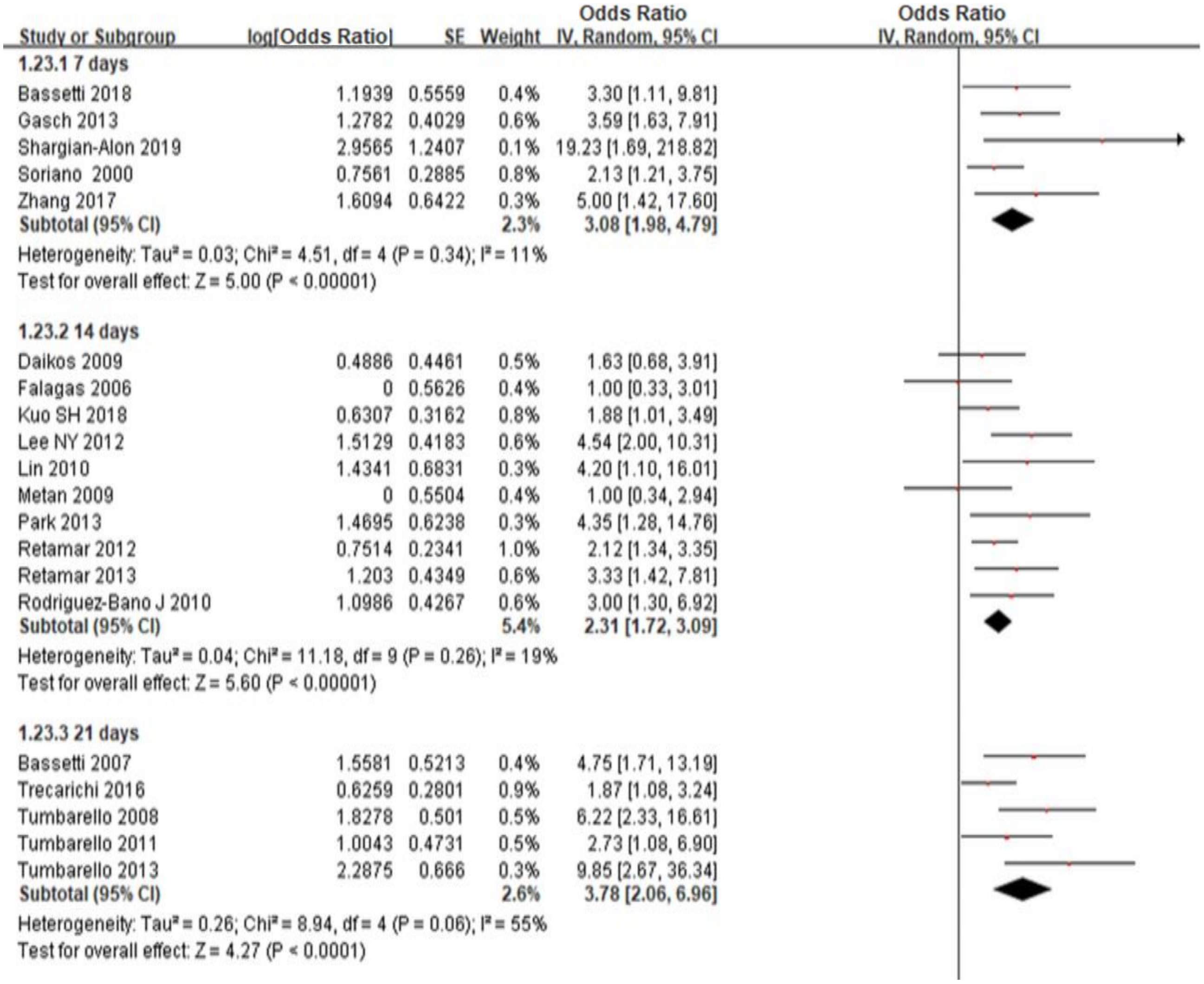

(4J) Varied morality deadline (continued)

Study	log[OR]	SE	Weight	Odds Ratio [95% CI]
**1.23.4 28 or 30 days**				
Adrie 2017	0.8329	0.1251	1.2%	2.30 [1.80, 2.94]
Bartoletti 2014	1.0716	0.3399	0.7%	2.92 [1.50, 5.68]
Bartoletti 2018	0.7608	0.0527	1.4%	2.14 [1.93, 2.37]
Bassetti 2012	1.026	0.4347	0.6%	2.79 [1.19, 6.54]
Bassetti 2018	1.1939	0.5559	0.4%	3.30 [1.11, 9.81]
Boel 2015	0.239	0.1271	1.2%	1.27 [0.99, 1.63]
Cain 2015	1.2641	0.3833	0.6%	3.54 [1.67, 7.50]
Chamot 2003	0.1823	0.7246	0.3%	1.20 [0.29, 4.97]
Chen 2013	0.571	0.1614	1.2%	1.77 [1.29, 2.43]
Cheng 2016	1.6601	0.542	0.4%	5.26 [1.82, 15.22]
Cheong 2008	1.2556	0.5605	0.4%	3.51 [1.17, 10.53]
Chusri 2019	-2.9957	0.8212	0.2%	0.05 [0.01, 0.25]
Dimopoulos 2015	0.7324	0.3488	0.7%	2.08 [1.05, 4.12]
Erbay 2009	0.8755	0.3128	0.8%	2.40 [1.30, 4.43]
Evans 2009	0.5539	0.5348	0.4%	1.74 [0.61, 4.96]
Falcone 2020	0.3235	0.1018	1.3%	1.38 [1.13, 1.69]
Fang 2006	-0.1863	0.1169	1.3%	0.83 [0.66, 1.04]
Gasch 2013	0.3148	0.1505	1.2%	1.37 [1.02, 1.84]
Girometti 2014	0.6419	0.2789	0.9%	1.90 [1.10, 3.28]
Gradel 2017	0.1621	0.093	1.3%	1.18 [0.98, 1.41]
Gudiol 2011	0.4511	0.5838	0.4%	1.57 [0.50, 4.93]
Hernandez 2015	0.3988	0.1884	1.1%	1.49 [1.03, 2.16]
Hsieh 2015	1.4563	0.4875	0.5%	4.29 [1.65, 11.15]
Jung 2012	0.8879	0.4185	0.6%	2.43 [1.07, 5.52]
Kang 2003	1.5282	0.6953	0.3%	4.61 [1.18, 18.01]
Kang 2005	0	0.3144	0.8%	1.00 [0.54, 1.85]
Kang 2013	1.1709	0.4298	0.6%	3.22 [1.39, 7.49]
Ku 2011	3.1747	1.4808	0.1%	23.92 [1.31, 435.73]
Lee 2013	1.1709	0.4298	0.6%	3.22 [1.39, 7.49]
Lee 2014	1.9544	0.3451	0.7%	7.06 [3.59, 13.88]
Lee 2015	1.914	0.4871	0.5%	6.78 [2.61, 17.61]
Lee 2017	0.9555	0.1654	1.1%	2.60 [1.88, 3.60]
Lee 2019	0.4762	0.1226	1.2%	1.61 [1.27, 2.05]
Lee CC 2012	0.8154	0.4109	0.6%	2.26 [1.01, 5.06]
Lodise 2007	1.411	0.6269	0.3%	4.10 [1.20, 14.01]
Lye 2012	0.1389	0.2356	1.0%	1.15 [0.72, 1.82]
Ma 2017	0.54	0.1548	1.2%	1.72 [1.27, 2.32]
Martinez 2010	0.5878	0.166	1.1%	1.80 [1.30, 2.49]
Martinez-Nadal 2020	1.1053	0.434	0.6%	3.02 [1.29, 7.07]
Migiyama 2016	1.5602	0.4527	0.5%	4.76 [1.96, 11.56]
Molina 2013	0	0.0829	1.3%	1.00 [0.85, 1.18]
Morata 2012	0.7793	0.2983	0.8%	2.18 [1.21, 3.91]
Ortega 2009	1.5748	0.1673	1.1%	4.83 [3.48, 6.70]
Ortega 2011	0	0.0262	1.4%	1.00 [0.95, 1.05]
Ortega 2012	0.3365	0.123	1.2%	1.40 [1.10, 1.78]
Ortega 2013	0.6206	0.1166	1.3%	1.86 [1.48, 2.34]
Park 2012	1.0043	0.4592	0.5%	2.73 [1.11, 6.71]
Park 2018	1.1756	0.2617	0.9%	3.24 [1.94, 5.41]
Park H 2015	1.1756	0.3929	0.6%	3.24 [1.50, 7.00]
Paul 2010	0.7655	0.2412	1.0%	2.15 [1.34, 3.45]
Pena 2013	0.5306	0.2759	0.9%	1.70 [0.99, 2.92]
Reisfeld 2011	0.3436	0.2523	0.9%	1.41 [0.86, 2.31]
Retamar 2012	0.4447	0.2218	1.0%	1.56 [1.01, 2.41]
Retamar 2013	0.6931	0.3798	0.6%	2.00 [0.95, 4.21]
Rodriguez-Bano 2010	0	0.3537	0.7%	1.00 [0.50, 2.00]
Rodriguez-Pardo 2014	1.0188	0.3868	0.6%	2.77 [1.30, 5.91]
Schweizer 2010	0.4055	0.2494	0.9%	1.50 [0.92, 2.45]
Son 2020	1.8148	0.533	0.4%	6.14 [2.16, 17.45]
Song 2011	0.7419	1.1997	0.1%	2.10 [0.20, 22.05]
Tagashira 2017	1.0225	0.3997	0.6%	2.78 [1.27, 6.09]
Takesue 2011	1.514	0.7248	0.3%	4.54 [1.10, 18.81]
Tam 2010	0	0.0316	1.4%	1.00 [0.94, 1.06]
Trecarichi 2009	2.7054	1.0396	0.1%	14.96 [1.95, 114.78]
Tumbarello 2012	1.4279	0.4856	0.5%	4.17 [1.61, 10.80]
Wang X 2017	0	0.3537	0.7%	1.00 [0.50, 2.00]
Wu 2015	1.4697	0.4542	0.5%	4.35 [1.79, 10.59]
Xie 2017	2.1136	0.2123	1.0%	8.28 [5.46, 12.55]
Ye 2006	0	0.5976	0.4%	1.00 [0.31, 3.23]
Zhang 2017	1.6094	0.3537	0.7%	5.00 [2.50, 10.00]
**Subtotal (95% CI)**			**52.7%**	**2.07 [1.82, 2.35]**

Heterogeneity: $Tau^2 = 0.18$; $Chi^2 = 695.48$, $df = 68$ ($P < 0.00001$); $I^2 = 90\%$

Test for overall effect: $Z = 11.05$ ($P < 0.00001$)

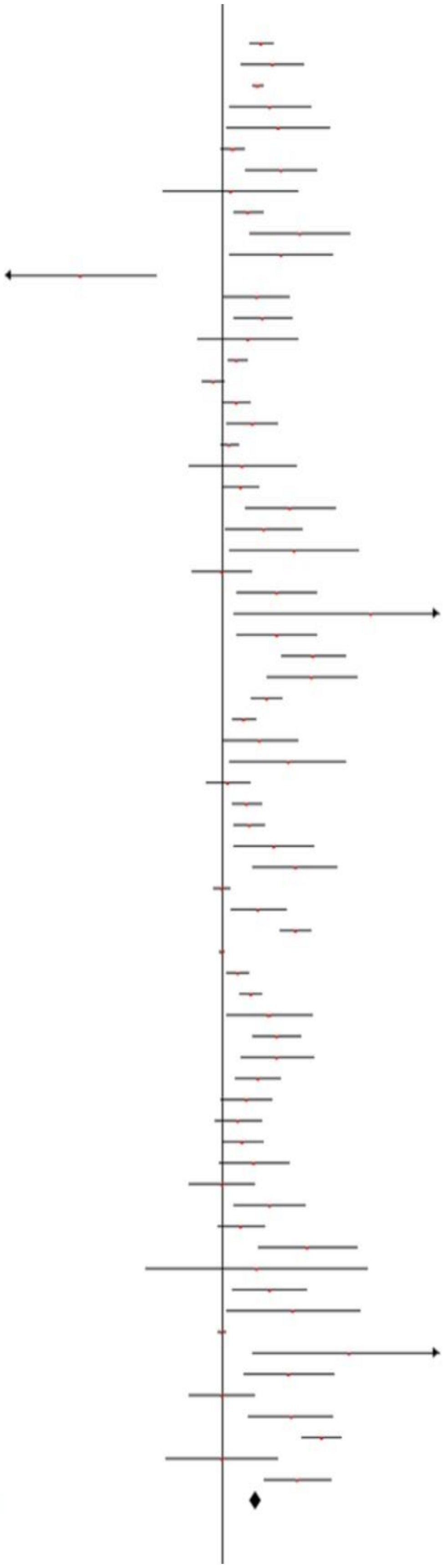

(4J) Varied morality deadline (continued)

**1.23.5 In-hospital**				
Al-Dorzi 2015	1.8976	0.9472	0.2%	6.67 [1.04, 42.69]
Anatoliotaki 2004	1.3863	0.3537	0.7%	4.00 [2.00, 8.00]
Bouza 2004	1.2528	0.4323	0.6%	3.50 [1.50, 8.17]
Bouza 2005	2.3609	1.1115	0.1%	10.60 [1.20, 93.63]
Byl 1999	0.7561	0.3149	0.8%	2.13 [1.15, 3.95]
Chang 2009	0	0.0595	1.4%	1.00 [0.89, 1.12]
Corona 2010	0.1133	0.1918	1.1%	1.12 [0.77, 1.63]
Elhanan 1997	0	0.2365	1.0%	1.00 [0.63, 1.59]
Endimiani 2003	1.454	0.9386	0.2%	4.28 [0.68, 26.94]
Evans 2009	0.0488	0.0674	1.4%	1.05 [0.92, 1.20]
Falagas 2006	0	0.0711	1.3%	1.00 [0.87, 1.15]
Garey 2006	0.4055	0.1629	1.2%	1.50 [1.09, 2.06]
Garnacho-Montero 2013	0.9163	0.3745	0.7%	2.50 [1.20, 5.21]
Guilarde 2006	0.8198	0.4082	0.6%	2.27 [1.02, 5.05]
Guillamet 2016	0.8329	0.4149	0.6%	2.30 [1.02, 5.19]
Ibrahim 2000	1.9257	0.1523	1.2%	6.86 [5.09, 9.25]
Johnson 2011	0.708	0.1027	1.3%	2.03 [1.66, 2.48]
Khan 2010	2.1223	0.4799	0.5%	8.35 [3.26, 21.39]
Khatib 2006	0	0.2198	1.0%	1.00 [0.65, 1.54]
Labelle AJ 2012	0	0.6316	0.3%	1.00 [0.29, 3.45]
Lee 2011	0	0.3537	0.7%	1.00 [0.50, 2.00]
Leibovici 1998	0.47	0.1059	1.3%	1.60 [1.30, 1.97]
Lodise 2003	1.335	0.5473	0.4%	3.80 [1.30, 11.11]
Micek & Lloyd 2010	0.7129	0.1848	1.1%	2.04 [1.42, 2.93]
Micek 2010	0.8329	0.1002	1.3%	2.30 [1.89, 2.80]
Ortega 2007	0.6931	0.2522	0.9%	2.00 [1.22, 3.28]
Peralta 2007	1.0919	0.4433	0.5%	2.98 [1.25, 7.10]
Peralta 2012	0.94	0.4645	0.5%	2.56 [1.03, 6.36]
Rebelo 2011	0	0.0051	1.4%	1.00 [0.99, 1.01]
Rong 2012	1.3863	0.5181	0.4%	4.00 [1.45, 11.04]
Sancho 2012	0.6575	0.3563	0.7%	1.93 [0.96, 3.88]
Savage 2016	0.0198	0.1921	1.1%	1.02 [0.70, 1.49]
Schechner 2011	0	0.0538	1.4%	1.00 [0.90, 1.11]
Schramm 2006	0.6523	0.1328	1.2%	1.92 [1.48, 2.49]
Shorr 2014	0.3492	0.13	1.2%	1.42 [1.10, 1.83]
Valles 2003	1.1725	0.3846	0.6%	3.23 [1.52, 6.86]
Yang 2013	1.3124	0.3882	0.6%	3.72 [1.74, 7.95]
Yu 2017	0.1275	0.5849	0.4%	1.14 [0.36, 3.57]
Zaragoza 2003	0	0.2439	0.9%	1.00 [0.62, 1.61]
Ziberberg 2014	1.3538	0.1709	1.1%	3.87 [2.77, 5.41]
**Subtotal (95% CI)**			**33.8%**	**1.81 [1.56, 2.10]**
Heterogeneity: $Tau^2 = 0.14$; $Chi^2 = 534.10$, df = 39 ($P < 0.00001$); $I^2 = 93\%$				
Test for overall effect: Z = 7.92 ($P < 0.00001$)				
**1.23.6 Long-term**				
Gradel 2017	0.3001	0.0908	1.3%	1.35 [1.13, 1.61]
Hanon 2002	0.4447	0.1381	1.2%	1.56 [1.19, 2.04]
Kim 2006	0.3293	0.4119	0.6%	1.39 [0.62, 3.12]
Yamaga & Shime 2017	3.1465	0.8452	0.2%	23.25 [4.44, 121.88]
**Subtotal (95% CI)**			**3.3%**	**1.68 [1.10, 2.54]**
Heterogeneity: $Tau^2 = 0.10$; $Chi^2 = 11.68$, df = 3 ($P = 0.009$); $I^2 = 74\%$				
Test for overall effect: Z = 2.43 ($P = 0.02$)				
**Total (95% CI)**			**100.0%**	**1.99 [1.84, 2.16]**
Heterogeneity: $Tau^2 = 0.12$; $Chi^2 = 1566.71$, df = 132 ($P < 0.00001$); $I^2 = 92\%$				
Test for overall effect: Z = 16.62 ($P < 0.00001$)				
Test for subgroup differences: $Chi^2 = 11.45$, df = 5 ($P = 0.04$), $I^2 = 56.3\%$				

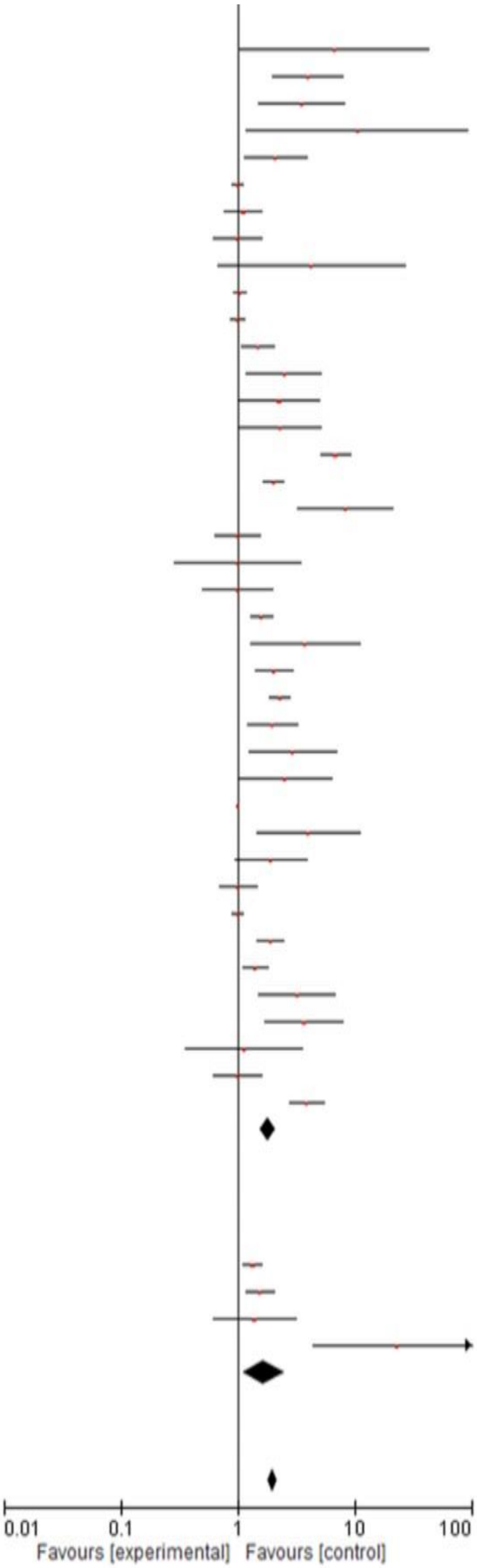

Supplement: Supplementary file 1 [file Data_Sheet_1.PDF]
